# Engineering transkingdom signalling in plants to control gene expression in rhizosphere bacteria

**DOI:** 10.1038/s41467-019-10882-x

**Published:** 2019-07-31

**Authors:** Barney A. Geddes, Ponraj Paramasivan, Amelie Joffrin, Amber L. Thompson, Kirsten Christensen, Beatriz Jorrin, Paul Brett, Stuart J. Conway, Giles E. D. Oldroyd, Philip S. Poole

**Affiliations:** 1 Department of Plant Sciences, University of Oxford, South Parks Road, Oxford, OX1 3RB UK; 2 Sainsbury Laboratory, University of Cambridge, Bateman Street, Cambridge, CB2 1LR UK; 3 Chemistry Research Laboratory, Department of Chemistry, University of Oxford, Mansfield Road, Oxford, OX1 3TA UK; 4 Department of Metabolic Biology, John Innes Centre, Norwich Research Park, Norwich, NR4 7UH UK

**Keywords:** Molecular engineering in plants, Applied microbiology, Secondary metabolism

## Abstract

The root microbiota is critical for agricultural yield, with growth-promoting bacteria able to solubilise phosphate, produce plant growth hormones, antagonise pathogens and fix N_2_. Plants control the microorganisms in their immediate environment and this is at least in part through direct selection, the immune system, and interactions with other microorganisms. Considering the importance of the root microbiota for crop yields it is attractive to artificially regulate this environment to optimise agricultural productivity. Towards this aim we express a synthetic pathway for the production of the rhizopine *scyllo*-inosamine in plants. We demonstrate the production of this bacterial derived signal in both *Medicago truncatula* and barley and show its perception by rhizosphere bacteria, containing bioluminescent and fluorescent biosensors. This study lays the groundwork for synthetic signalling networks between plants and bacteria, allowing the targeted regulation of bacterial gene expression in the rhizosphere for delivery of useful functions to plants.

## Abstract (web summary)

The root microbiota is critical for promoting crop yield. Here, the authors create a synthetic pathway for the production of the rhizopine *scyllo*-inosamine in *Medicago truncatula* and barley, and show its perception by rhizosphere bacteria for targeted regulation of bacterial gene expression.

## Introduction

The root microbiota, like the gut microbiota in human health, is critical for plant health and agricultural yield^1,2^. It is shaped by plant selection^3,4^ and its immune system^5^, as well as complex interactions between microorganisms. Plant growth-promoting bacteria can alter nutrient availability and antagonise pathogens. Perhaps, the most important plant–microbe interaction is between legume plants and symbiotic bacteria called rhizobia. Natural transkingdom signalling between them is essential for the establishment of rhizobia in plant root organs called nodules. In root nodules rhizobia reduce atmospheric N_2_ to ammonia, producing a substantial proportion of the biosphere’s available nitrogen.

**Fig. 1:**
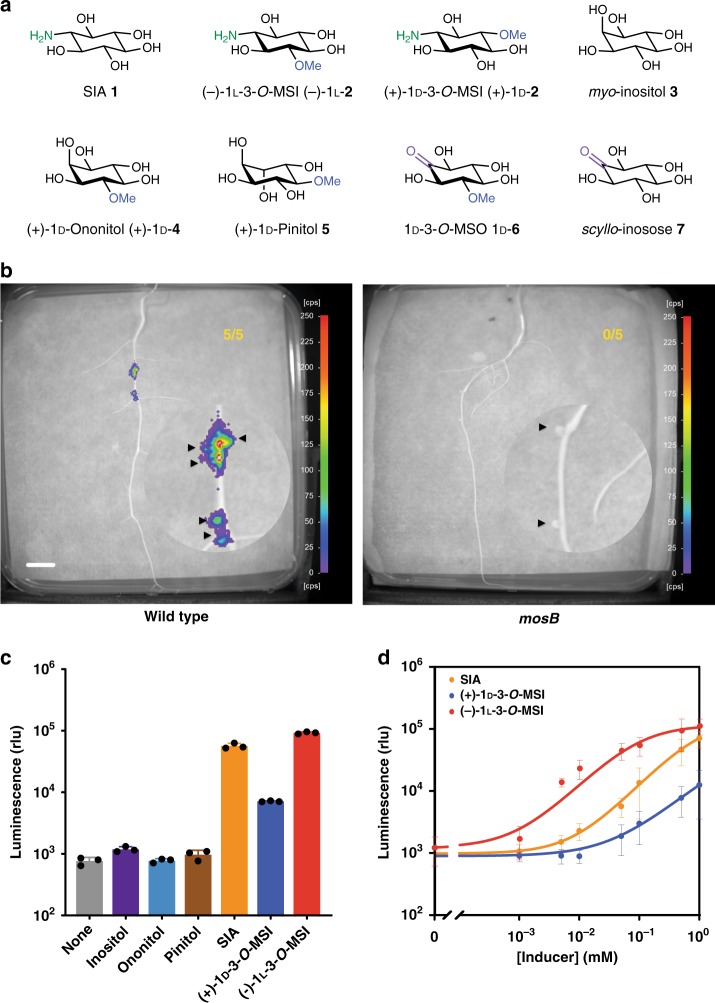
Detection of rhizopine exudation with a rhizopine biosensor. **a** Chemical structures of rhizopines and related cyclitols. **b** NightOwl images of bioluminescence of *R. leguminosarum* Rlv3841/pOPS0046 rhizopine *lux* biosensor on the surface of *M. sativa* roots nodulated by *S. meliloti* L5-30 wild-type (rhizopine+) and *S. meliloti* L5-30 *mosB*:pK19 (rhizopine –, see Fig. 2a). Numbers in top right corners indicate number of plants tested that showed significant levels of bioluminescence (*n* = 5). Circle in bottom right corner contains magnified image of a root section enriched with nodules. The positions of nodules are indicated with arrowheads (scale bar, 1 cm). Colours represent luminescence intensity from 0 counts per second (cool/purple) to 250 counts per second (warm/red). **c** Specificity of induction of rhizopine *lux* biosensor with rhizopines compared to other chemically similar plant polyols, grown in UMS minimal medium with pyruvate and ammonia as carbon and nitrogen sources, supplemented with 1 mM of inducing molecule. Data are presented as relative luminescence units (rlu). Black dots indicate individual data points for each condition. **d** Induction curves showing the dynamic range and sensitivity of the rhizopine *lux* biosensor with chemically synthesised SIA **1**, (−)-1l-3-*O*-MSI (−)-1l-**2** and (+)-1d-3-*O*-MSI (+)-1d-**2** grown in UMS minimal medium with pyruvate and ammonia as carbon and nitrogen sources. Error bars represent standard deviation of the mean of *n* = 3 independent replicates. All experiments were repeated at least three independent times

The root microbiota represents an enormous potential for improving crop yields by engineering the plant microbiome. Enhancing crop productivity in a sustainable manner may be achieved in several ways, including transfer of bacterial N_2_ fixation to cereals^6–9^, harnessing bacterial phosphate solubilisation, promoting root growth by bacterial synthesis of plant hormones, or pathogen antagonism by antibiotic production. These plant growth-promoting services by bacteria could be regulated by engineering plants to produce a synthetic transkingdom signal to control bacteria on roots. Such a signal must be: (1) amenable to engineering in plants, (2) able to elicit a bacterial response, (3) exuded into the rhizosphere (zone of soil surrounding the roots) and (4) not normally be made by plants. Transgenic plants that produced opine molecules, repurposed from *Agrobacterium*, have been shown to enrich their rhizosphere with bacteria able to catabolize opines; however, utilizing a nutritional mediator of pathogenic organisms as a signal is undesirable^10–12^. A group of compounds, called rhizopines, were recognised as ideal chemical signals, although efforts to engineer rhizopine-producing plants in the past were unsuccessful^13^. Rhizopines (*scyllo*-inosamine **1** (SIA) and 3-*O*-methyl-*scyllo*-inosamine **2** (3*-O*-MSI)) (Fig. 1a) are synthesised by a few species of rhizobia in legume nodules, under the control of the NifA regulator, during N_2_-fixing symbiosis^14^. These molecules are rare in nature^15^ and absent from most plant rhizospheres. Rhizobia are able to synthesise rhizopines and utilise them as carbon and nitrogen sources. Genes involved in the biosynthesis (*mosABC*) and catabolism of rhizopines (*mocCABRDEF*) have been identified in the wild-type rhizobium *Sinorhizobium meliloti* L5-30^16,17^. Although the natural role of rhizopines remains to be elucidated, previous studies suggested that they may be exuded into the rhizosphere^14,18^.

In this study, we demonstrate the exudation of rhizopines into the rhizosphere by a rhizobium–legume symbiosis and elucidate the biochemical pathway for 3-*O*-MSI **2** biosynthesis by rhizobia in legume nodules. We show that while the 3-*O*-MSI **2** biosynthetic pathway is not readily amenable to transfer to plants, the biosynthesis of the rhizopine SIA **1** is. Finally, we establish synthetic SIA-mediated transkingdom signalling from transgenic *Medicago truncatula* and barley plants to bacteria in their rhizospheres.

## Results

### Rhizopine exudation during rhizobial symbiosis

To validate rhizopines as targets for transkingdom signalling, we first wished to confirm they are exuded into the plant rhizosphere in their natural context (synthesis by rhizobia in N_2_-fixing root nodules). To investigate exudation into the rhizosphere, a rhizopine biosensor (pOPS0046) was made by cloning the *mocB* promoter (encodes a putative ATP transporter substrate-binding protein in the rhizopine catabolism locus) and its divergent regulator *mocR*^19^ into a *lux* reporter vector^20^. Exudation of rhizopine into the rhizosphere was measured by bacterial luminescence on roots^20^ of the natural host of *S. meliloti* L5-30, *Medicago sativa*. Plants were co-inoculated with *S. meliloti* L5-30 (which nodulates and produces rhizopine) and *Rhizobium leguminosarum* (Rlv3841) carrying pOPS0046 (contains the biosensor but cannot nodulate). *Medicago sativa* plants nodulated by *S. meliloti* L5-30 caused bioreporter Rlv3841 to bioluminesce on the root surface adjacent to nodules (Fig. 1b, Supplementary Fig. 1), confirming rhizopines traverse plant tissues to reach the root surface.

To confirm that the biosensor responds specifically to rhizopines, SIA **1** was obtained via a revised synthetic route, and both enantiomers of 3-*O-*MSI, (−)-1l-**2** and (+)-1d-**2**, were synthesised using a protection-resolution strategy (Supplementary Figs. 2 and 3). The absolute configuration of key synthetic intermediates was confirmed by nuclear magnetic resonance analysis (Supplementary Table 5) and single-crystal X-ray diffraction. Induction of the biosensor was tested in free-living culture in response to SIA **1**, (−)-1l-3-*O*-MSI (−)-1l-**2** and (+)-1d-3-*O*-MSI (+)-1d-**2**, and the naturally abundant cyclitols *myo*-inositol **3**, (+)-1d-ononitol (+)-1d-**4** and (+)-1d-pinitol (+)-1d-**5** (Fig. 1a). The biosensor was activated by SIA **1** and (−)-1l-3-*O*-MSI (−)-1l-**2** (Fig. 1c, d). (−)-1l-3-*O*-MSI (−)-1l-**2** has previously been proposed to be the naturally occurring enantiomer^21^, and the preference for this enantiomer observed in Fig. 1c, d supports this. Furthermore, (−)-1l-3-*O*-MSI (−)-1l-**2** promoted the growth of *S. meliloti* L5-30, whereas (+)-1d-3-*O*-MSI (+)-1d-**2** did not (Supplementary Fig. 4), supporting results from a recent study^21^. The confirmation of the chemical structure of rhizopines, and their secretion into the rhizosphere, provides a unique opportunity to use them as target molecules for engineering plant control of root bacteria.

### Elucidation of the natural 3-*O*-MSI biosynthetic pathway

In *S. meliloti* L5-30 the genes *mosABC* were proposed to encode the pathway for 3-*O*-MSI biosynthesis^13,17^. MosA was thought to be a methyltransferase^22^, while MosB and MosC are homologous to aminotransferase and transmembrane transport proteins, respectively^13,16^. However, rhizopines were not produced when *mosABC* was expressed in *Arabidopsis thaliana*^13^. Furthermore, the role of MosA in 3-*O*-MSI synthesis has been questioned^23^, suggesting *mosABC* may not encode the complete rhizopine pathway. Bioinformatic analysis of rhizobial genomes indicated that *mosA* is not widely distributed among putative rhizopine loci (Supplementary Fig. 5). In addition, three genes, which we name *mosDEF*, were found downstream of *mosC* in many putative rhizopine loci. Comparative analytical chemistry by gas chromatography-mass spectrometry (GC-MS), using nodule extracts from several of these rhizobia and chemically synthesised (±)-3-*O*-MSI (±)-**2** as a standard, revealed that *mosBCDEF* are present in all rhizobia that synthesised 3-*O*-MSI (Supplementary Fig. 5).

**Fig. 2:**
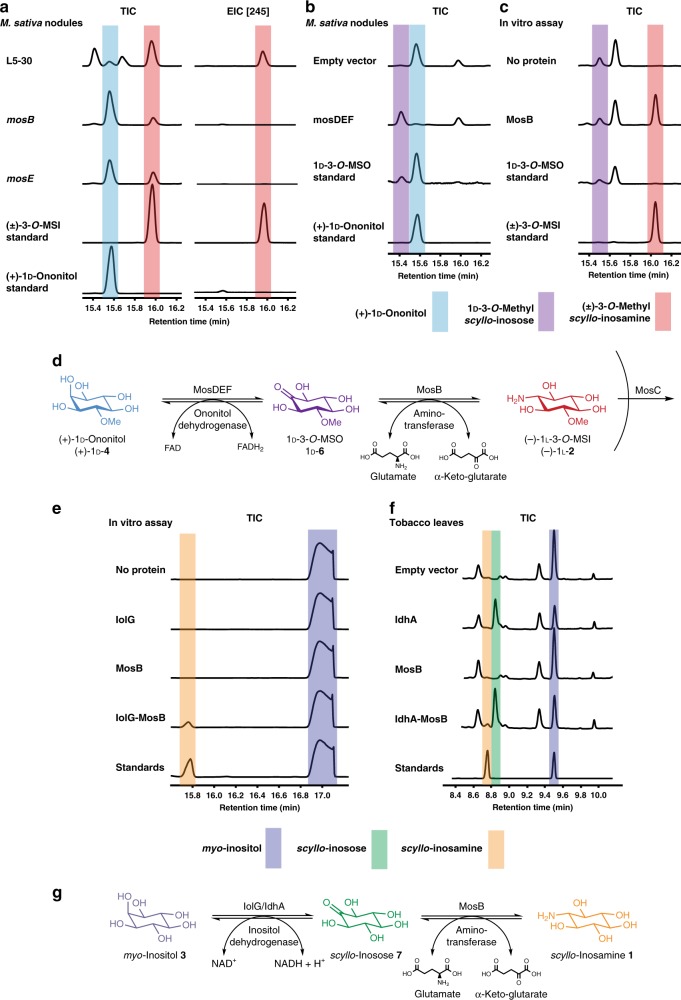
Discovery of a natural and synthetic pathway for rhizopine synthesis. **a** Gas chromatography-mass spectrometry (GC-MS) total ion chromatograms (TICs) (left) and extracted ion chromatograms (*m/z* 245 = 3-*O*-MSI-TMS) (EIC [245]) (right) of metabolites from nodules formed by wild-type *S. meliloti* L5-30 compared to *mosB*:pK19 and *mosE*:pK19 mutants. Chemically synthesised (±)-3-*O*-MSI (±)-**2** and purchased (+)-1d-ononitol (+)-1d-**4** as standards are shown below. **b** GC-MS chromatograms of metabolites from nodules formed by wild-type *S. meliloti* Rm1021 maintaining an empty vector (EV) and expressing *mosDEF*. Chemically synthesised (±)-3-*O-*methyl-*scyllo*-inosose (±)-**6** and purchased (+)-1d-ononitol (+)-1d-**4** as standards are shown below. **c** GC-MS chromatograms (TIC) of MosB in vitro assay in the absence of protein or with MosB added. Chemically synthesised (±)-3-*O-*methyl-*scyllo*-inosose (±)-**6** and (±)-3-*O*-MSI (±)-2 as standards are shown below. **d** Proposed natural pathway of 3-*O-*MSI biosynthesis in *S. meliloti* L5-30. **e** Linked in vitro assay of inositol dehydrogenase IolG and MosB. GC-MS TICs of assay mix in the absence of added protein, with IolG added, with MosB added and with IolG and MosB added together. Standards represent a mix of SIA **1** and *myo*-inositol **3**. **f** GC-MS TICs of extracts prepared from tobacco leaves agro-infiltrated with either empty vector (control) or IdhA or MosB or IdhA-MosB together. Standards represent a mix of SIA **1** and *myo*-inositol **3**. **g** Proposed synthetic pathway of SIA **1** synthesis. Metabolites were identified by comparison with authentic standards. Highlighted peaks indicate *scyllo*-inosamine **1** (orange), *scyllo*-inosose **7** (green), *myo*-inositol **3** (dark blue), (+)-1d-ononitol **4** (light blue), 1d-3-*O*-methyl-*scyllo*-inosose **6** (purple) and (−)-1l-3-*O*-methyl-*scyllo*-inosamine **2** (3-*O*-MSI) (red). All chromatograms are representative of experiments repeated at least three independent times. Source data underlying  **a**–**c**, **e** and **f** are provided as a Source Data file

Supporting the role of *mosDEF* in 3-*O*-MSI synthesis, a *S. meliloti* L5-30 strain disrupted in *mosE* showed no detectable rhizopine synthesis in nodules (Fig. 2a). Based on homology, MosDEF is likely to be a membrane-bound flavin adenine dinucleotide-dependent dehydrogenase complex^24^. We hypothesized that the dehydrogenase complex, MosDEF, oxidises (+)-1d-ononitol (+)-1d-**4** at the axial C-2 hydroxyl group to form 3-*O*-methyl-*scyllo*-inosose 1d-**6**. This keto-inositol intermediate could undergo reductive amination catalysed by MosB to generate (−)-1l-3-*O*-MSI (−)-1l-**2**.

To test this hypothesis, *mosDEF* was expressed in *S. meliloti* Rm1021 nodules (strain that does not produce rhizopines) and a compound accumulated that had an identical retention time and mass spectrum to a peak that eluted immediately before (+)-1d-ononitol (+)-1d-**4** in nodules produced by wild-type *S. meliloti* L5-30, but not in *mosE* or *mosB* mutants (Fig. 2a, b). We hypothesized that this compound was 3-*O*-methyl-*scyllo-*inosose **6**. To identify this compound, (±)-3-*O*-methyl-*scyllo-*inosose (±)-**6** was synthesised to serve as a chemical standard in GC-MS experiments. Although chemically synthesised (±)-3-*O*-methyl-*scyllo-*inosose (±)-**6** was unstable in solution, following derivatisation and GC-MS analysis, a unique peak was observed with an identical retention time and mass spectrum to the compound produced in nodules formed by Rm1021 expressing *mosDEF* (Fig. 2b). This result is consistent with MosDEF being an ononitol dehydrogenase. Neither *mosE* nor *mosB* mutants appeared to accumulate (±)-3-*O*-methyl-*scyllo-*inosose (±)-**6**, but both accumulated substantial amounts of (+)-1d-ononitol (+)-1d-**4** (Fig. 2a). The absence of accumulation of (±)-3-*O*-methyl-*scyllo-*inosose (±)-**6** in *mosE* is predictable. However, its absence in *mosB* may be caused by spontaneous conversion to (+)-1d-ononitol (+)-1d-**4**, as observed in our chemical standard, or due to polarity of the *mosB* insertion mutation on *mosDEF* transcription.

Next, MosB was purified and when incubated with chemically synthesised 3-*O*-methyl-*scyllo-*inosose (±)-**6**, the cofactor pyridoxal 5′-phosphate (PLP), and l-glutamate, 3-*O*-MSI **2** was produced (Fig. 2c, Supplementary Fig. 6a). Therefore, MosB functions as a 3-*O*-methyl-*scyllo-*inosose: l-glutamate aminotransferase generating 3-*O*-MSI **2**.

Initial tests of 3-*O*-MSI synthesis in plants using *Agrobacterium*-mediated transient expression of *McIMT* (encodes *myo*-inositol *O*-methyltransferase to produce (+)-1d-ononitol (+)-1d-**4** from *myo-*inositol **3**), *mosDEF* and *mosB* together in *Nicotiana benthamiana* leaves did not yield 3-*O*-MSI **2** (Supplementary Fig. 7). MosDEF may need to interact with the bacterial membrane and/or the symbiotic bacterial electron transport chain to oxidise (+)-1d-ononitol (+)-1d-**4** to 1d-3-*O*-methyl-*scyllo-*inosose 1d-**6** (the substrate for MosB).

### Engineering of SI biosynthesis in plants

To date, MosDEF is the only (+)-1d-ononitol dehydrogenase identified, making transfer of the natural pathway of 3-*O*-MSI synthesis to plants difficult; therefore, an alternative was sought. Since MosB uses 3-*O*-methyl-*scyllo-*inosose **6** as the substrate backbone for transamination by l-glutamate, we considered that *scyllo*-inosose **7** (produced from *myo*-inositol **3** by bacterial inositol dehydrogenase^25,26^) may also act as a transamination substrate for MosB to generate SIA **1**. IolG, the *myo*-inositol dehydrogenase from the Gram-positive bacterium *Bacillus subtilis* has been crystallized and the subject of extensive biochemical characterization^27–30^, and we previously identified a *myo-*inositol dehydrogenase in the Gram-negative *R. leguminosarum* (IdhA)^26^. We tested both *myo-*inositol dehydrogenases for activity in plants and similar activity was observed for both *iolG* and *idhA* in *N. benthamiana* after transient infection (Supplementary Fig. 8). Following protein purification *B. subtilis* inositol dehydrogenase (IolG) showed higher activity in vitro (Supplementary Fig. 9), and was used in an in vitro assay of MosB *scyllo*-inosose: l-glutamate aminotransferase activity. While IolG showed robust enzyme activity in vitro, we were unable to observe *scyllo*-inosose **7** accumulation by GC-MS. We expect this was a result of instability of *scyllo*-inosose **7** in aqueous solutions, which we observed when we attempted its chemical synthesis. Nevertheless, when IolG was combined with MosB, *myo-*inositol **3** and NAD^+^ (substrate/cofactor for IolG), as well as l-glutamate and PLP, SIA **1** was only produced in the presence of both IolG and MosB (Fig. 2e, Supplementary Fig. 6b). Therefore, MosB also has *scyllo*-inosose: l-glutamate aminotransferase activity to generate SIA **1**. In this synthetic pathway, bacterial inositol dehydrogenase oxidises *myo-*inositol **3** to *scyllo*-inosose **7** and MosB catalyses the glutamate transamination of **7** to generate SIA **1** (Fig. 2g). We expected this synthetic pathway to be amenable to transfer of SIA synthesis into plants.

To test whether this synthetic pathway is transferable to plants, inositol dehydrogenase from *R. leguminosaurum* (IdhA) and *scyllo*-inosose: l-glutamate aminotransferase (MosB) from *S. meliloti* were expressed in *N. benthamiana* leaves, and SIA **1** was produced (Fig. 2f, Supplementary Fig. 10). This represents the first successful transkingdom transfer of rhizopine (SIA) synthesis from bacteria to plants. Furthermore, it should enable control of members of the plant microbiota using the MocR regulator that responds to SIA **1** (Fig. 1c).

### Establishment of rhizopine transkingdom signalling

**Fig. 3:**
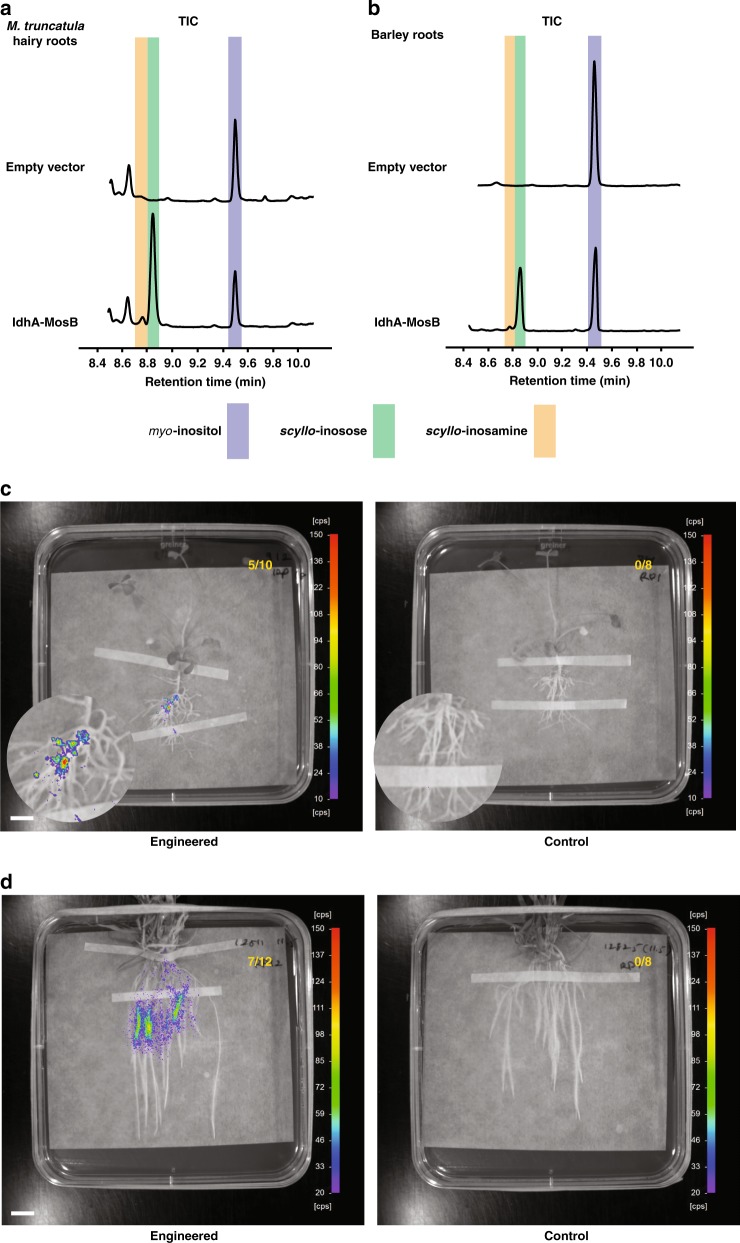
Rhizopine biosynthesis and signalling in *M. truncatula* and barley roots. **a**, **b** Gas chromatography-mass spectrometry (GC-MS) total ion chromatograms (TICs) of extracts prepared from *M. truncatula* transgenic roots (**a**) and transgenic T_0_ barley seedlings (**b**) transformed with empty vector (control) or IdhA-MosB together. Highlighted peaks indicate *scyllo*-inosamine **1** (orange), *scyllo*-inosose **7** (green) and *myo*-inositol 3 (dark blue). All chromatograms are representative of experiments repeated at least three independent times. Source data for GC-MS chromatograms are provided as a Source Data file in Supplementary Materials. **c**, **d** NightOwl images showing bioluminescence of Rlv3841/pOPS0046 rhizopine *lux* biosensor on the surface of *M. truncatula* transgenic roots (**c**) and T_0_ barley seedlings (**d**) transformed with empty vector (control) or IdhA-MosB together (engineered). Numbers in top right corners indicate number of plants tested that showed significant levels of bioluminescence (scale bar, 1 cm). Colours represent luminescence intensity from 10 or 20 counts per second (cool/purple) to 150 counts per second (warm/red). Source data underlying **a**, **b** are provided as a Source Data file

To demonstrate the potential of rhizopine as a transkingdom signal to bacteria in the rhizosphere, the synthetic pathway was transferred by *Agrobacterium rhizogenes*-mediated root transformation into *Medicago truncatula*. SIA was detected in transgenic hairy roots as well as in transgenic root organ cultures of *M. truncatula* expressing *idhA* and *mosB* under the control of constitutive promoters (Fig. 3a, Supplementary Fig. 11). *Medicago truncatula* hairy roots were found to produce 27.39 ng mg^−1^ dry weight SIA [SE = 9.38; *N* = 3] (Supplementary Data 1) and SIA **1** was exuded into the rhizosphere at sufficient levels for Rlv3841, carrying the rhizopine lux biosensor, to luminesce (Fig. 3c, Supplementary Fig. 11). No luminescence was observed when the rhizopine lux biosensor was inoculated on the transgenic roots constitutively expressing *idhA* alone that produced *scyllo*-inosose (Supplementary Fig. 12). This confirmed that rhizopine acts as a transkingdom signal to bacteria, rather than the *scyllo*-inosose intermediate, in the rhizopine synthetic pathway engineered into plants.

The most important targets for transfer of rhizopine-mediated transkingdom signalling are cereals, where there are large global efforts to engineer plant growth promotion, disease resistance and N_2_ fixation by bacteria. To assess if we could engineer rhizopine synthesis in cereals, we transferred the rhizopine biosynthesis genes into *Hordeum vulgare* (barley).

**Fig. 4:**
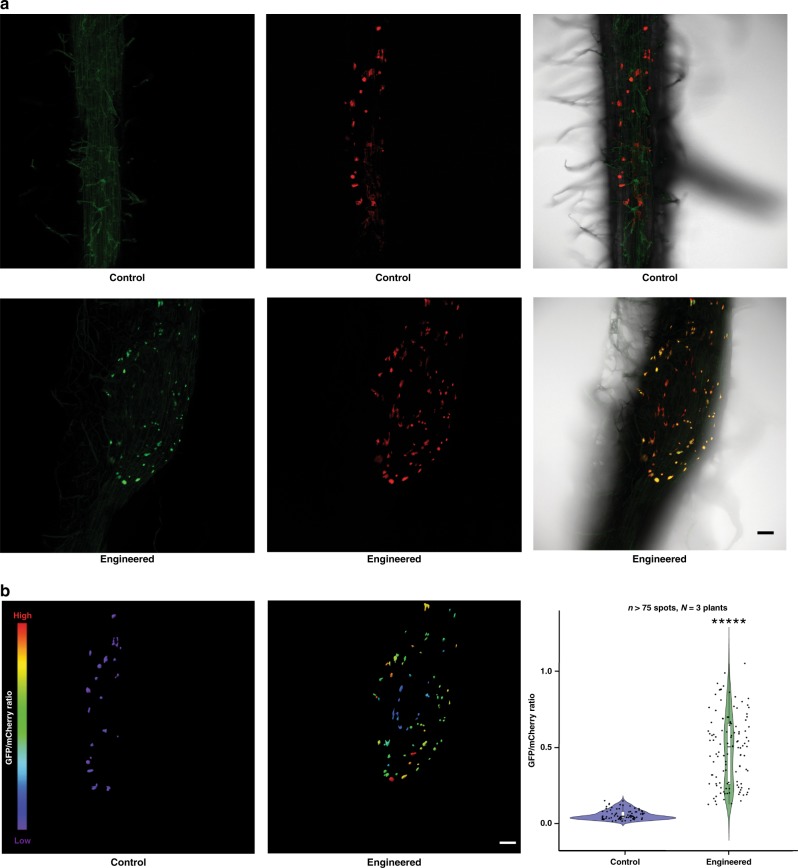
Fluorescent microscopy of rhizopine-mediated transkingdom signalling. **a** Confocal fluorescence microscopy images showing green fluorescent protein (GFP) fluorescence of Rlv3841::mTn7-mCherry/pOPS0761 biosensor [with constitutive mCherry fluorescence] on the surface of T_1_ barley seedlings transformed with empty vector (control) or IdhA-MosB together (engineered). Z-stack projections of green fluorescence channel (left, GFP), red fluorescence channel (middle, mCherry) and all channels merged (right, bright-field) are shown (scale bars, 100 µm). Images shown are representative of experiments repeated two independent times. **b** Three-dimensional images of GFP/mCherry mean intensity ratios in Rlv3841::mTn7-mCherry/pOPS0761 biosensor on the surface of T_1_ barley seedlings transformed with empty vector (left, control) or IdhA-MosB together (middle, engineered) (scale bars, 100 µm). Cool colours (purple) indicate low GFP/mCherry intensity ratio, and warm colours (red) indicate a high GFP/mCherry intensity ratio. Violin plot of GFP/mCherry ratios in the biosensor colonies (right; *n* > 75 spots; *N* = 3 plants). Asterisks signify statistical significance (Wilcoxon’s signed-rank test, *P* = 1.30 × 10^−14^). Images shown are representative of experiments repeated two independent times

SIA **1** production was observed in transgenic roots of T_0_ and T_1_ barley plants by GC-MS analysis (Fig. 3b). T_0_ and T_1_ barley plants were found to produce 6.55 ng mg^−1^ dry weight [SE = 0.43; *n* = 10] and 7.00 ng mg^−1^ dry weight [SE = 0.17; *n* = 10, *N* = 2] of rhizopine in their transgenic roots, respectively (Supplementary Data 2–4). SIA **1** produced in transgenic roots of T_0_ barley plants acted as a transkingdom signal to bacteria containing the rhizopine lux biosensor (Fig. 3d). As cereals are our intended final target plants and transgenic seed is available for rhizopine secreting barley, we measured the signalling between barley roots and bacteria in the rhizosphere using a green fluorescent protein (GFP) rhizopine biosensor. Confocal images revealed that rhizopine produced in transgenic roots of T_1_ barley plants induced GFP fluorescence at a significant level in most bacterial colonies carrying the rhizopine GFP biosensor on the root surface (Fig. 4a, b). Thus, a circuit of plant-dependent synthesis and signalling by SIA **1** to bacteria in the rhizosphere has been established.

## Discussion

The synthetic transkingdom signalling pathway established in this work opens up the microbial pan-genome to plant control. For example, it could activate specific members of the microbiota to fix N_2_, produce antibiotics or hormones, chelate iron or solubilise soil nutrients^31^. Control of these traits by the host plant would prevent off-target effects in species such as weeds that would be caused by constitutive expression. Furthermore, the inducible expression system we established here will help alleviate growth penalties incurred by constitutive overexpression of energetically intensive plant growth promotion traits in microbes. Finally, escape of genetically modified bacterial inoculants into the environment could be addressed by integrating plant signal recognition with biocontainment strategies^32^.

A key example requiring host control of a microbe is the aim of transferring N_2_ fixation to alleviate the demand for chemical fertilizers in agriculture. To achieve this aim, cereals must control N_2_ fixation by bacteria that grow on either the surface of roots (epiphytes), inside roots (endophytes) or by bacteria that reside in engineered nodules^7–9^. In analogy to natural transkingdom signalling used by legumes to control rhizobial symbioses, rhizopine transkingdom signalling could control synthetic symbioses to deliver nitrogen to cereal crops.

## Methods

### Bacterial strains and growth media

Bacterial strains and plasmids used are shown in Supplementary Tables 1 and 2, respectively. *Escherichia coli* strains were grown at 37 °C in Luria–Bertani (LB) medium^33^ with antibiotics: ampicillin (100 μg ml^−1^), tetracycline (10 μg ml^−1^), gentamicin (10 μg ml^−1^), or kanamycin 20 μg ml^−1^). *Rhizobium leguminosarum* and *Sinorhizobium meliloti* strains were grown in tryptone yeast (TY) medium^34^ or universal minimal salts (UMS)^20^ at 28 °C. Antibiotics added to TY included streptomycin (500 μg ml^−1^), tetracycline (5 μg ml^−1^) and gentamicin (20 μg ml^−1^) for *R. leguminosarum*, and streptomycin (200 μg ml^−1^) or neomycin (80 μg ml^−1^ for plasmids or 200 μg ml^−1^ for pK19 mutants) for *S. meliloti*. *Agrobacterium tumefaciens* and *A. rhizogenes* were cultured in LB medium at 28 °C with antibiotics: rifampicin (50 μg ml^−1^), gentamycin (40 μg ml^−1^), tetracycline (5 µg ml^−1^) or kanamycin (25 μg ml^−1^) for *A. tumefaciens*, and rifampicin (20 µg ml^−1^), kanamycin (20 μg ml^−1^) or carbenicillin (50 μg ml^−1^) for *A. rhizogenes*.

### Plant materials and sterilisation

*Medicago sativa* seeds were sterilised with 95% ethanol for 45 min followed by 6% sodium hypochlorite for 5 min, and germinated on distilled water agar in the dark for 2 days at room temperature. *Pisium sativum* seeds were sterilized with 70% ethanol for 1 min, washed with water, and then with 2% sodium hypochlorite for 5 min, rinsed thoroughly and germinated on distilled water agar in the dark for 2 days at room temperature. *Medicago truncatula* ecotype Jester was used for *A. rhizogenes* transformation. Seeds were scarified with sand paper, sterilised in 1% sodium hypochlorite for 3 min, incubated on agar (1.5%) in the dark for 3 days at 4 °C for stratification and then overnight at room temperature for germination. Seed sterilization and germination of spring barley genotype Golden Promise was performed using standard protocols^35^. Briefly, barley seeds were washed in 70% ethanol for 3 min, sterilised in 5% sodium hypochlorite for 4 min, incubated on agar (0.8%) in the dark for 3 days at 4 °C for stratification and then 3 days at room temperature for germination.

### Bacterial genetic manipulations and plasmid construction

PCR was performed with Phusion High-Fidelity DNA Polymerase (Thermo Fisher Scientific) using primers in Supplementary Table 4. The biosensor pOPS0046 was constructed by restriction/ligation of a *mocRB* amplicon from *S. meliloti* L5-30^36^ into pIJ11268^36^. Plasmids for pK19 mutant construction (pOPS0243, pOPS0244) were constructed by cloning internal fragments of *S. meliloti* L5-30 *mosB* and *mosD* into pK19mob^37^ by BD In-Fusion cloning (Clontech). The plasmid for IolG purification (pOPS0241) was constructed by BD cloning of *Bacillus subtilis iolG* into pOPINF^38^.

The plasmid for nodule expression of *mosDEF* (pOPS0362) was constructed using *Saccharomyces cerevisiae* homologous recombination^39^. A modified version of the Gram-negative expression vector pMQ131^40^ that contained the *par* locus for stability in the environment was used for recombineering with PmosB and *mosDEF* amplicons from *S. meliloti* L5-30.

The plasmid pOPS0363 used for MosB purification was assembled by golden gate cloning^41–43^. *Sinorhizobium meliloti* tauR/Ptau taurine promoter, HIS-tagged maltose-binding protein, *E. coli* codon optimised *mosB* and rrnBT1 terminator were assembled in linear order into pOGG024^44^.

The rhizopine GFP biosensor plasmid pOPS0761 was constructed by assembly of the *mocRB* amplicon from pOPS0046 with an amplicon containing the *GFPmut3* open reading frame into the modified pMQ131 vector described above by *S. cerevisiae* homologous recombination^39^. The resulting plasmid was conjugated into Rlv3841::mTn7-mCherry (Rlv3841 containing constitutively expressed mCherry introduced into the genome with pUC18T-miniTn7T-Gm^45,46^).

All plasmids were verified by restriction digest and DNA sequencing. Plasmids were transferred by conjugation into rhizobia by tri-parental mating with pRK2013^47^.

### Generation of constructs for plant engineering

Constructs for plant engineering were assembled using golden gate cloning^41–43^ (Supplementary Table 3). For transient expression, *R. leguminosarum idhA* and *S. meliloti* L5-30 *mosB* were expressed under the control of CaMV 35s promoter (35s) and *Lotus japonicus* Ubiquitin1 (LjUBI1) promoter, respectively. *Sinorhizobium meliloti mosDEF* and *M. crystallinum IMT* were expressed transiently under the control of 35s and LjUBI1 promoters, respectively. In barley transgenic lines, *idhA* and *mosB* were expressed under the control of *Zea mays* ubiquitin1 promoter and *Oryza sativa* ubiquitin1 promoter, respectively. All bacterial genes were codon optimised^48^ for expression in plants. DNA components (promoters, coding sequences and terminators) were synthesised by GeneArt (Life Technologies). Recombinant clones were verified by PCR and DNA sequence analysis. Primers used are listed in Supplementary Table 4.

### Bioinformatic identification of rhizopine loci

Putative rhizopine loci were identified by BLAST query of the Joint Genome Institute Integrated Microbial Genomes (IMG) database (https://img.jgi.doe.gov/) with *S. meliloti* L5-30 MosB and the IMG Ortholog Neighbourhood Viewer tool^49^. Putative rhizopine loci in other bacteria were selected that contained homologues to either *mosC* or *mosA* in addition to *mosB*. Genetic diagrams of the loci were copied from the IMG website and redrawn with Adobe Illustrator.

### Metabolite extraction and GC-MS analysis of root nodules

Growth of *M. sativa* or *P. sativum* plants for nodule extraction was carried out in 1 L pots filled with sterile vermiculite and nitrogen (N_2_)-free rooting solution^50^. Ten germinated *M. sativa* or two germinated *P. sativum* seedlings per pot were inoculated with 1 × 10^7^ colony-forming units (CFUs) of rhizobia in distilled water 3 days after planting. Plants were grown for 6 to 8 weeks before harvesting pink N_2_-fixing nodules by hand. Pooled nodules from one pot (<100 mg) were ground using a mortar and pestle in liquid nitrogen. Enzymes were inactivated by the addition of 1400 μl 100% methanol and incubated for 10 min at 70 °C. For metabolite extraction, 750 μl of CHCl_3_ and 1400 μl of dH_2_O was added, samples were vortexed and then centrifuged for 15 min at a relative centrifugal force (RCF) of 2200. Aliquots of the supernatant were then dried in a vacuum concentrator prior to derivitization^51^. Samples were derivatised by trimethylsilylation prior to GC-MS analysis by incubation with 25 μl of pyridine and 35 μl of trimethylsilylimidazole (TMSI) (Sigma 33068) for 60 min at 37 °C with shaking at 950 RPM. GC-MS analysis was performed using the LJS_TMSI protocol at the University of Oxford Department of Plant Sciences (Supplementary Methods). For data generated in Supplementary Fig. 5, GC-MS was performed with the LJS_Golm Stardard protocol (Supplementary Methods).

### Protein purification

For purification of IolG and IdhA, pOPS0141 and pOPS0142 were transformed into BL21-competent *E. coli* (New England Biolabs) and grown overnight in 5 ml LB^Amp^. The next day, the full volume was subcultured to 50 ml LB^Amp^. At OD_600_ (optical density at 600 nm) of 0.5, 1 mM isopropyl β-d-1-thiogalactopyranoside (IPTG) was added. After 3 h of induction cells were pelleted at 4 °C, frozen with liquid nitrogen and stored at −80 °C. Cell pellets were thawed on ice, resuspended in 1 ml of HIS-binding buffer and disrupted by ribolyzing. Lysate was clarified by centrifugation at 4 °C. Purification of HIS-tagged proteins from the lysate was performed using a His-Spin Protein Miniprep Kit (Zymo Research).

For purification of MosB, *R. leguminosarum* 3841 maintaining pOPS0363 was grown on a TY agar slope with antibiotics for 3 days, washed with UMS and used to inoculate six baffled flasks containing 500 ml of UMS (containing 20 mM succinate, 10 mM NH_4_Cl and 10 mM taurine) to OD_600_ of 0.01. Cultures were grown until they reached an OD_600_ of 0.5, pelleted at 4 °C, frozen in liquid nitrogen and stored at −80 °C. Cell pellets were thawed on ice and resuspended in 15 ml extraction buffer (MBPTrap binding buffer (20 mM Tris-HCl, 200 mM NaCl, 1 mM EDTA, pH 7.4) containing 1 mM dithiothreitol and PMSF Protease Inhibitor (Thermo Fisher)). Cells were disrupted by ribolysing and clarified by centrifugation at 4 °C. The clarified lysate was filtered (0.45 μm) and loaded onto a 1 ml MBPTrap HP column on an AKTA Basic 10 (GE Healthcare Life Sciences). The column was washed with running buffer and eluted with running buffer containing 10 mM maltose.

Purity of purified proteins was assessed by sodium dodecyl sulfate-polyacrylamide gel electrophoresis analysis and proteins were quantified using a Qubit 2.0 Fluorometer (Thermo Fisher) according to the manufacturer’s instructions.

### MosB in vitro assays

The MosB in vitro assay contained 100 mM Tris-HCl, pH 7.5, 0.9 mM PLP, 5 mM (±)-3-*O*-methyl-*scyllo*-inosose (±)-**6** and 5 mM of amino acid and water to a total volume of 100 μl (±)-3-*O*-methyl-*scyllo*-inosose (±)-**6** was used immediately following its synthesis due to instabilities observed. The linked assay with IolG used the reaction mixture described above, as well as 5 mM NAD^+^ and 5 mM *myo-*inositol **3** as substrate rather than 3-*O*-methyl-*scyllo*-inosose. Proteins were added to a concentration of 10 μg ml^−1^. Assays were incubated for 24 h at 28 °C. Enzyme activity was halted by boiling for 10 min and protein was removed by centrifugation. The supernatant was dried in a vacuum centrifuge and derivatised by resuspending in 25 μl pyridine and 35 μl TMSI at 37 °C with shaking for 60 min. GC-MS analysis was performed with the LJS_TMSI protocol at the University of Oxford Department of Plant Sciences (Supplementary Methods).

### Transient expression in *Nicotiana benthamiana*

Recombinant plasmids were mobilised into *A. tumefaciens* GV3101:pMP90 by electroporation. A transformed single colony was grown in LB with appropriate antibiotics for 24 h, and then subcultured in fresh LB and grown overnight. Cells were harvested by centrifugation and resuspended in infiltration buffer (10 mM MES buffer, pH 5.6, 10 mM MgCl_2_ and 150 µM acetosyringone) to an OD_600_ of 0.5. After incubating at room temperature in the dark for 3 h, *A. tumefaciens* strains were mixed with an equal volume of a P19 suppressor strain and infiltrated into the underside of *N. benthamiana* leaves using a needleless 1 ml syringe. Leaf discs were harvested 3 days after infiltration, frozen in liquid nitrogen and the extracted metabolites were analysed by GC-MS.

### Hairy-root transformation in *Medicago truncatula*

*Agrobacterium rhizogenes* AR1193 was transformed with recombinant constructs by electroporation. A transformed single colony was grown on LB agar plates with antibiotics for 48 h. Cells were removed using sterile toothpicks and suspended in 1 ml of sterile water. Germinated seedlings of *M. truncatula* cultivar Jester were transformed with *A. rhizogenes* AR1193 using standard protocols^52^. Briefly, under sterile conditions cut tip of radicle approximately 3 mm from the root tip of each germinated seedlings, dip the ends of each cut seedlings into *A. rhizogenes* AR1193 culture and grown them on ModFP agar plates. Four weeks after transformation, composite plants were screened on the basis of fluorescent enhanced GRP (eGFP) marker. eGFP-positive transgenic roots were harvested and frozen in liquid nitrogen before extracting metabolites for GC-MS analysis.

For cultivation of axenic hairy-root cultures, 4 weeks after transformation of *M. truncatula* seedlings by *A. rhizogenes* AR1193, transgenic roots of approximately 3 to 5 cm in length were excised and subcultured every 3 weeks on M agar plates containing 350 µg ml^−1^ of cefotaxime sodium. For GC-MS analysis, 3-week-old transgenic hairy-root organ cultures grown on Modified M agar plates (containing 0.4 mM NH_4_NO_3_) were harvested, frozen in liquid nitrogen and extracted metabolites were analysed.

### Stable transformation of *Hordeum vulgare*

Recombinant binary vectors were transferred by electroporation into *A. tumefaciens* AGL1 strain. Transformation of barley by recombinant *Agrobacterium* strains was performed using standard protocols^35^. For rapid testing in T_0_ barley plants, approximately 12 weeks after transformation, transgenic roots from barley plantlets were harvested, frozen in liquid nitrogen and the extracted metabolites were analysed by GC-MS. Approximately 2-week-old T_1_ barley plants were used for confocal microscopy and GC-MS analysis.

### GC-MS analysis of engineered plant tissue

Frozen plant materials were lyophilized to dryness, ground to a fine powder and extracted with 70% ethanol at 70 °C for 60 min. The resulting extract was centrifuged at an RCF of 15,871 or 10 min and the supernatant was evaporated to dryness. For GC-MS analysis, dried metabolic samples were dissolved in appropriate volume of 70% ethanol, 50 μl aliquots were dried down and trimethylsilylated using Tri-Sil Z reagent (Sigma, catalogue no. 92718) at 80 °C for 60 min. GC-MS analysis was performed using the Eng_Plant protocol at the John Innes Centre (Supplementary Methods). Rhizopine production in engineered plant samples were determined using a calibration curve (concentration vs. peak area) drawn by analysing a set of rhizopine standards of known concentrations in GC-MS. MassHunter qualitative analysis software (Agilent) was used to determine the peak area of rhizopine in EIC (*m*/*z* 245) chromatograms of the standards and plant samples.

### Bacterial luciferase biosensor screening assays

For imaging of rhizopine synthesis in *M. sativa* sterilised, germinated seedlings were placed onto square plates containing FP-agar overlaid with filter paper. Roots were inoculated at the time of planting with 5 × 10^7^ cells (*S. meliloti* L5-30 strain and *R. leguminosarum* Rlv3841 carrying the rhizopine lux biosensor mixed 1:1)^20^. Plants were imaged after the formation of N_2_-fixing nodules at 2 weeks post inoculation.

For imaging rhizopine secretion on transgenic *M. truncatula* hairy roots, 4-week-old composite plants with transgenic roots were transferred to new ModFP agar plates and inoculated with Rlv3841 carrying the rhizopine lux biosensor. For transgenic *M. truncatula* root organ cultures, 1-week-old hairy-root explants were transferred to new Modified M agar plates without antibiotics and inoculated with *R. leguminosarum* Rlv3841 carrying the rhizopine lux biosensor. Both transgenic *M. truncatula* hairy roots and root organ cultures were imaged every week post inoculation.

For imaging rhizopines secretion on transgenic T_0_ barley plant roots, approximately 12-week-old primary T_0_ barley transformants with strong root growth were transferred from regeneration medium to ModFP agar petridishes without antibiotics and *R. leguminosarum* Rlv3841 carrying the rhizopine lux biosensor was inoculated on the engineered roots. Plants were imaged each day post-inoculation.

Bioluminescence images were analysed for quantification using imaging software IndiGO (Berthold Technologies) and data were expressed as the ratio of luminescence to surface (cps mm^−2^).

### Confocal microscopy and image analysis

For imaging rhizopine-mediated signalling on transgenic T_1_ barley plant roots, 3-day-old germinated seedlings were grown in ModFP agar Petri dishes and Rlv3841 strain carrying the rhizopine GFP-mCherry biosensor was inoculated on the engineered roots. Plants were imaged from 10 days post inoculation.

Confocal images were taken using a Leica SP8-FLIMan with an HC PLAN APO ×10/0.40 dry objective. The 488 and 552 nm lasers were used to excite GFP and red fluorescent protein (mCherry), respectively. Fluorescence emissions were collected using HyD SMD detectors set to detect GFP (500–530 nm) and mCherry (600–630 nm). The 488 and 552 nm lasers were set at 4 and 2% with detector gain at 12 and 36 for GFP and mCherry protein fluorescence imaging, respectively. A line average of three was used and z-slices were acquired with a step size of 2.41 µm.

Confocal images were analysed using IMARIS 8.3.1 (Bitplane) and ImageJ software’s. Image processing and analysis were done using IMARIS 8.3.1 as previously described^53^. Briefly, mCherry (RFP) channel was used to segment surfaces (i.e. individual bacterial colonies) using the ‘surfaces wizard’ with background subtraction and thresholding set at default values 11 µm and 361, respectively. Surfaces were false-coloured based on the mean of the masked GFP and mCherry (RFP) channels. IMARIS Xtension ‘XT Mean Intensity Ratio’ was used to calculate the GFP/mCherry intensity ratio of individual surfaces^53^. The mean intensity ratios (GFP/mCherry) was presented in a violin plot drawn by using R-programming. Wilcoxon’s signed-rank test analysis was used to test the level of significance.

### Analysis of growth and bioreporter expression in culture

Analysis of expression of bioreporters in free-living culture was performed with either a FLUOstar OMEGA (Lite) or CLARIOstar plate reader (BMG Labtech). Strains were grown in UMS-defined medium with a starting OD_600_ of 0.1. UMS was supplemented with either 30 mM sodium pyruvate as a carbon source or 10 mM NH_4_Cl as a nitrogen source. Cultures were grown and monitored in black 24-well plates clear bottom plates covered with a gas-permeable moisture barrier seal (4titude) at 28 °C shaking at 500 RPM on the orbital setting. Measurements of luminescence were taken at 30-min intervals. Data are expressed as relative luminescence units calculated from total luminescence per well/ culture density measured by OD_595_.

### Reporting summary

Further information on research design is available in the Nature Research Reporting Summary linked to this article.

## Supplementary information

Supplementary Information

Peer Review

Reporting Summary

Description of Additional Supplementary Files

Supplementary Data 1

Supplementary Data 2

Supplementary Data 3

Supplementary Data 4

Source Data

## Data Availability

Data supporting the findings of this work are available within the paper and its Supplementary Information files. A reporting summary for this Article is available as a Supplementary Information file. The datasets generated and analysed during the current study are available from the corresponding author upon request. The source data underlying Figs. 2a–c, 2e, 2f, 3a and 3b as well as Supplementary Figs. 5b, 7, 8a, 10 and 11a are provided as a Source Data file. The x-ray crystallographic coordinates for 1d-19 and 1l-19 reported in this study have been deposited at the Cambridge Crystallographic Data Centre (CCDC) under deposition numbers CCDC 1919781 [https://www.ccdc.cam.ac.uk/structures/Search?Ccdcid=CCDC%201919781&DatabaseToSearch=Published, 10.5517/ccdc.csd.cc22fpfn] and CCDC 1919782 [https://www.ccdc.cam.ac.uk/structures/Search?Ccdcid=CCDC%201919782a&DatabaseToSearch=Published, 10.5517/ccdc.csd.cc22fpgp], respectively. These data can be obtained free of charge from The Cambridge Crystallographic Data Centre via www.ccdc.cam.ac.uk/data_request/cif.
