# Supplementary material for: Engineering transkingdom signalling in plants to control gene expression in rhizosphere bacteria
Source: Nat Commun. 2019 Jul 31;10:3430. doi: 10.1038/s41467-019-10882-x (PMC6668481; doi:10.1038/s41467-019-10882-x)
Supplement: Supplementary file 1 — Supplementary Information [file 41467_2019_10882_MOESM1_ESM.pdf]

Engineering transkingdom signalling in plants to control gene expression in rhizosphere bacteria

Geddes *et al.*

Supplementary Methods

General experimental

^{1}H NMR spectra were recorded on Bruker DPX 200 (200 MHz), Bruker AVIIIHD 400 nanobay (400 MHz), Bruker AVII 500 (500 MHz) with dual ^{13}C(^{1}H) cryoprobe, or Bruker AVIIIHD 500 (500 MHz) spectrometer in the stated solvents as a reference for the internal deuterium lock. The chemical shift data for each signal are given as δ_H in units of parts per million (ppm) relative to tetramethylsilane (TMS) where δ_H (TMS) = 0.00 ppm. The spectra are calibrated using the solvent peak with the data provided by Fulmer *et al*[1]. The multiplicity of each signal is indicated by s (singlet); br s (broad singlet); d (doublet); dd (doublet of doublets), ddd (doublet of doublet of doublets), t (triplet), q (quartet), dq (double of quartet) or m (multiplet). The number of protons (n) for a given resonance signal is indicated by nH. Where appropriate, coupling constants (J) are quoted in Hz and are recorded to the nearest 0.1 Hz. Identical proton coupling constants (*J*) are averaged in each spectrum and reported to the nearest 0.1 Hz. The coupling constants are determined by analysis using Bruker TopSpin version 3.2 software. ^{1}H spectra were assigned using 2D NMR experiments including COSY, HMBC, HSQC, ^{29}Si-^{1}H HMBC and ^{31}P-^{1}H HMBC. ^{13}C NMR spectra were recorded on a Bruker AVIIIHD 400 nanobay (101 MHz), or Bruker AVII 500 (126 MHz) spectrometer, with dual ^{13}C(^{1}H) cryoprobe, in the stated solvents, with broadband proton decoupling and an internal deuterium lock. The chemical shift data for each signal are given as δ_C in units of parts per million (ppm) relative to tetramethylsilane (TMS) where δ_C (TMS) = 0.00 ppm. The spectra are calibrated using the solvent peak with the data provided by *Fulmer et al*[1]. The shift values of resonances are quoted to 1 decimal place unless peaks have similar chemical shifts, in which case 2 decimal places are used. ^{13}C spectra were assigned using 2D NMR experiments including HMBC and HSQC. When two diastereoisomers are present in the sample, A and B denotes each of the two diastereoisomers without distinguishing between them. A is arbitrarily assigned to the diastereoisomer with the highest ppm shift and B to the diastereoisomer with the lowest ppm shift, in ^{1}H NMR and ^{13}C NMR spectra.

Low resolution electrospray ionisation mass spectra were acquired on a Waters LCT Premier spectrometer or Agilent 6120 Quadrupole spectrometer. High resolution mass spectra were recorded on a

Bruker MicroTOF spectrometer, operating in positive or negative mode, as indicated, from solutions of MeOH, MeCN or H_2O. *m/z* values are reported in Daltons and followed by their percentage abundance in parentheses. Electron ionisation/field ionisation (EI/FI) was carried out on a Waters GCT with a temperature programmed solids probe inlet. MALDI was carried out on a Waters MALDI Micro MX. When a compound was not observed by LRMS, only HRMS is quoted.

Specific optical rotations were measured using either a Perkin Elmer Model 241 polarimeter or Schmidt + Haensch UniPol L2000 polarimeter, in cells with a path length of 1 dm, using a sodium lamp at 589 nm. The concentration (*c*) is expressed in g/100 mL (equivalent to g/0.1 dm^3). Specific rotations are denoted $[\alpha]_D^T$ and are given in implied units of $10^{-1} deg cm^2 g^{-1}$ at the temperature stated. Melting points were determined using a Leica Galen III hot stage microscope and are uncorrected. The solvents of crystallisation are shown in parentheses. Infrared (IR) spectra were obtained from neat samples, either as liquids or solids using a diamond ATR module. The spectra were recorded on a Bruker Tensor 27 spectrometer. Absorption maxima are reported in wavenumbers (cm^{-1}) and reported as s (strong), m (medium), w (weak) or br (broad). Only the main, relevant peaks have been assigned.

Thin layer chromatography (TLC) was carried out on normal phase Merck silica gel 60 F_{254} aluminium-supported chromatography sheets. Visualisation was by absorption of UV light (λ_{max} 254 nm), exposure to iodine vapour or thermal development after dipping in either an ethanolic solution of ninhydrin or an aqueous solution of potassium permanganate.

Normal phase silica gel flash column chromatography was performed either manually using VWR Prolabo silica gel 60 (240–400 mesh) under a positive pressure of nitrogen or on a Biotage SP1 automated column chromatography system using KP-Sil® SNAP Flash Silica Cartridges.

Chemicals were purchased from Acros UK, Apollo Scientific, Enamine, Sigma Aldrich UK, Alfa Aesar UK, Fisher Scientific UK, Fluka UK, Fluorochem, Merck, Argo International Limited and TCI-Europe. All reagents were purified, when necessary, by standard techniques[2]. In particular, Et_3N and pyridine were dried by stirring over solid KOH pellets overnight followed by fractional distillation. DIPA was distilled from NaH. These were stored under Ar and over 3 Å molecular sieves. Anhydrous solvents were obtained under the following conditions: Et_2O, toluene and CH_2Cl_2 were dried by passing through a column of activated basic

alumina according to the Grubbs' procedure.[3]Anhydrous DMF, DMSO, MeOH and MeCN were purchased from Sigma Aldrich UK in SureSeal™ bottles and used without further purification. Anhydrous THF was distilled from sodium metal, using benzophenone as an indicator[2]. All other solvents were used as supplied (analytical or HPLC grade) without purification.

In vacuo refers to the removal of solvents under reduced pressure using a Büchi™ rotary evaporator in a water bath at 40 °C, unless otherwise stated. Where appropriate and if not otherwise stated, all non-aqueous reactions were performed in a flame dried flask under an inert atmosphere. Hexane refers to a mixture of hexane isomers and petroleum ether refers to the fraction of light petroleum ether boiling within the range of 40–60 °C. Brine refers to a saturated aqueous solution of sodium chloride. Rochelle's salt refers to an aqueous solution of potassium sodium tartrate tetrahydrate. Lyophilisation refers to the removal of H_2O from aqueous solutions by freeze drying using a CHRIST Alpha 1-2 LD lyophiliser. Celite® refers to Celite® 545 filter aid, treated with sodium carbonate, flux-calcined which was purchased from Sigma Aldrich. Glass microfiber filter refers to Whatman® borosilicate glass microfiber filters, Grade GF/B.

Compound purity was determined by analytical high-performance liquid chromatography (HPLC) on a PerkinElmer Flexar system with a Binary LC Pump and UV/VIS LC Detector using: a) a reversed phase Dionex Acclaim® 120 column (C18, 5 μm, 4.6 × 150 mm) with H_2O/MeCN/TFA 95:5:0.1 (A) and MeCN/ H_2O/TFA 95:5:0.1 (B) or H_2O/MeCN 95:5 (A) and MeCN/H_2O 95:5 (B) as eluents; or b) a normal phase HyperSil GOLD™ Silica column (5 μm, 4.6 × 150 mm) with heptane (A) and IPA (B) as eluents. Gradient methods of 19 to 25 minutes were employed with a constant flow rate, and detection at 254 nm (Methods 1, 2 and 3). Samples were injected by dissolving in the relevant solvent system. The methods used are described below:

Method 1 (Reversed Phase HPLC gradient Supplementary Table 6): A = H_2O/MeCN/TFA 95:5:0.1; B = MeCN/ H_2O/TFA 95:5:0.1; 1.0 mL.min^{-1}; 254 nm.

Method 2 (Reversed Phase HPLC gradient Supplementary Table 6): A = H_2O/MeCN 95:5; B = MeCN/ H_2O 95:5; 1.0 mL.min^{-1}; 254 nm.

Method 3 (Normal Phase HPLC gradient Supplementary Table 7): Normal Phase; A = Heptane; B = IPA; 1.0 mL.min^{-1}; 254 nm.

Enantiomeric purity was determined by chiral analytical high-performance liquid chromatography (HPLC) on a PerkinElmer Flexar system with a Binary LC Pump and UV/VIS LC Detector using a ChiralPak® AD-H column (5 μ m, 4.6 × 150 mm) with IPA (A) and heptane (B) as eluents. Isocratic methods of 45 minutes were employed with a constant flow rate of 1.0 mL.min^{-1} or 0.8 mL.min^{-1} and detection at 220 or 254 nm as indicated. Samples were injected by dissolving in the relevant solvent system. The enantiomeric excess (*e.e*) was determined using the following equation: *e.e.* = $((R-S)/R+S)) \times 100$ where R and S stand for the individual enantiomers and $R + S = 1$.

GC-MS analysis was performed at two sites: the University of Oxford Department of Plant Sciences (Oxford, UK), or the John Innes Centre (Norwich, UK). GC-MS analysis of nodule tissues and *in vitro* assays were performed at the University of Oxford Department of Plant Sciences using an Agilent 7989 GC coupled to an Agilent 5975 quadrupole MS detector, electron 702 impact ionisation (70 eV) equipped with an Agilent CP9013 column (30 mm, 0.25 mm inner diameter). Analysis was performed with a 0.6 mL/min constant helium flow with the following oven temperatures: 150 °C for 2 min, ramped to 300 °C at 5 °C per min, then ramped to 330 °C at 10 °C per min, then dropped to 150 °C at 150 °C per min, and held for 1 min. The front inlet, source and transfer line temperature were set throughout at 230 °C, 250°C and 250 °C respectively (LJS_TMSI protocol). Alternatively, the following oven temperatures were used for the LJS_Golm Stardard protocol: 70 °C for 5 min, ramped to 350 °C at 5 °C per min, then dropped to 330 °C at 120 °C per min, held for 5 min, then dropped to 70 °C at 120 °C per min, and held for 1 min. The front inlet, source and transfer line temperature were set throughout at 230 °C, 250°C and 250 °C respectively. GC-MS of engineered plant tissues was performed at the John Innes Centre (Eng_Plant protocol). A 1 µl derivatised sample was injected into a ZB-5HT column (Phenomenex, 35 m x 0.25 mm internal diameter, 0.25 µm film) in Agilent 7890B GC machine and operated at a constant helium flow of 1 ml per min. The oven temperature program used was as follows: 100 °C for 1 min, ramped to 200 °C at 20 °C per min, held at 200 °C for 3 min, ramped to 300 °C at 30 °C per min and held at 300 °C for 2 min. The front inlet, source and transfer line temperature were set throughout at 250 °C, 230 °C and 280 °C respectively. NISTv2.2 library was used to assign identity to peaks

in the GC-MS chromatogram. SIA and 3-*O*-MSI were identified based on the comparison of retention times and mass spectra to chemical standards synthesised in this work. GC-MS data were analysed using MassHunter Qualitative Analysis software (Agilent) or MSD Chemstation Enhanced Chemstation software (Agilent).

Synthetic protocol and characterisation data for *myo*-inositol 1,3,5-orthoformate (8)

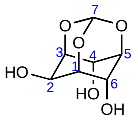

(**8**)

To a solution of *myo*-inositol **3** (20.0 g, 111 mmol, 1.0 eq) in DMF (80 mL) were added triethylorthoformate (82.5 g, 555 mmol, 5.0 eq) and PTSA·H_2O (8.45 g, 44.5 mmol, 0.40 eq). The reaction mixture was stirred at 105 °C for 3 days, then allowed to cool to RT. The solution was neutralised by addition of solid $NaHCO_3$ (10 g). The volatile components were removed *in vacuo* to give a yellow paste which was taken up in MeOH (300 mL). The mixture was cooled to −20 °C for 13 hours, then filtered to remove excess solid $NaHCO_3$ and sodium tosylate by-products. The filtrate was concentrated *in vacuo* to give a brown oil which was taken up in MeOH (50 mL) and cooled to −20 °C for 2 days. The resulting colourless crystalline solid was isolated by filtration, washed with $CHCl_3$ (25 mL), and dried. The remaining filtrate was concentrated *in vacuo* and purified by column chromatography over silica gel (CH_2Cl_2/MeOH 9.5:0.5) to give another batch of the crystalline solid. The combined materials provided **8** (14.9 g, 65%): mp: 300–302 °C (lit.[4,5] 300–302 °C); TLC (MeCN:EtOAc, 8:2 v/v): R_f = 0.40; ^{1}H NMR (400 MHz; D_6-DMSO): δ 5.48 (br s, 1H, C(2)O*H*), 5.44–5.36 (m, 2H, C(4)O*H* and C(6)O*H*), 5.31 (d, J = 6.2 Hz, 1H, H-7) 4.31–4.24 (dd, J = 3.9, 3.9 Hz, 2H, H-1 and H-3), 4.09–4.04 (m, 1H, H-5), 4.02–3.98 (m, 1H, H-2), 3.97–3.92 (m, 2H, H-4 and H-6); ^{13}C NMR (101 MHz; D_6-DMSO): δ 101.9 (C-7), 74.4 (C-1, C-3), 69.3 (C-5), 67.5 (C-4, C-6), 58.6 (C-2); LRMS (m/z): $[M+H]^+$ 191.2. These data are in good agreement with the literature values[4,5].

Synthetic protocol and characterisation data for 4,6-di-*O*-benzyl-*myo*-inositol 1,3,5-orthoformate (9)

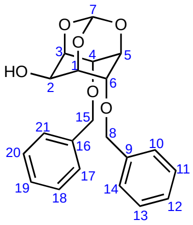

(**9**)

LiH (167 mg, 21.0 mmol, 4.0 eq) was gradually added to a solution of **8** (1.00 g, 5.26 mmol, 1.0 eq) in anhydrous DMF (15 mL), and the resulting solution was stirred at RT for 30 minutes. Benzyl bromide (1.44 mL, 12.1 mmol, 2.3 eq) was then added dropwise and the solution was stirred for a further 48 hours. After quenching the reaction with H_2O (30 mL), the aqueous phase was extracted with EtOAc (3 × 30 mL). The combined organic layers were washed with H_2O (4 × 30 mL), and brine (2 × 30 mL), then dried ($MgSO_4$), filtered, and concentrated *in vacuo*. The residue obtained was filtered through a short plug of silica gel (Petroleum ether/EtOAc 6:4) then crystallised (EtOAc) to yield **9** (1.37 g, 71%) as a colourless solid: mp: 123–124 °C (lit.[6] 122–124 °C; lit.[7] 124–125 °C); TLC (Petroleum ether:EtOAc, 1:1 v/v): R_f = 0.48; ^{1}H NMR (400 MHz; $CDCl_3$): δ 7.31–7.26 (m, 10H, H-10 to H-14 and H-17 to H-21), 5.47 (d, J = 1.0 Hz, 1H, H-7), 4.67 (d, J_{AB} = 11.5 Hz, 2H, H-8 and H-15), 4.59 (d, J_{AB} = 11.5 Hz, 2H, H-8' and H-15'), 4.48–4.45 (m, 1H, H-2), 4.38 (dd, J = 3.7, 3.7 Hz, 2H, H-4 and H-6), 4.25–4.18 (m, 3H, H-1, H-3 and H-5), 3.04 (d, J = 11.5 Hz, 1H, C(2)O*H*); LRMS (m/z): $[M+Na]^+$ 393.1. These data are in good agreement with the literature values[5–8].

Synthetic protocol and characterisation data for 2-*O*-allyl-4,6-di-*O*-benzyl-*myo*-inositol 1,3,5-orthoformate (10)

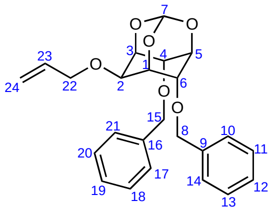

(**10**)

A solution of **9** (800 mg, 2.36 mmol, 1.0 eq) in anhydrous DMF (14 mL) was cooled to 0 °C and NaH (170 mg, 7.09 mmol, 3.0 eq, 60% dispersion in mineral oil) was added portionwise over 5 minutes. The

reaction mixture was left to stir at 0 °C for 10 minutes, then warmed to RT, and stirred for a further 30 minutes. After addition of allyl bromide (0.614 mL, 7.09 mmol, 3.0 eq) at 0 °C, followed by stirring for 10 minutes, the reaction mixture was warmed to RT, and left to stir for a further 17 hours. The reaction was then quenched with H_2O (5 ml) and diluted in EtOAc (5 mL). The aqueous phase was extracted with EtOAc (4 × 10 mL), and the combined organic layers were washed with H_2O (2 × 10 mL), and brine (2 × 10 mL), then dried ($MgSO_4$), filtered, and concentrated *in vacuo*. Purification by column chromatography over silica gel (Petroleum ether/EtOAc 9:1, 8:2, 7:3, 6:4, 5:5) afforded **10** (893 mg, 92%) as a thick colourless oil: TLC (Petroleum ether:EtOAc, 1:1 v/v): R_f = 0.73; ^{1}H NMR (400 MHz; $CDCl_3$): δ 7.35–7.27 (m, 10H, H-10 to H-14 and H-17 to H-21), 6.04–5.91 (m, 1H, H-23), 5.53 (s, 1H, H-7), 5.35–5.27 (m, 1H, H-24), 5.24–5.18 (m, 1H, H-24'), 4.69 (d, J_{AB} = 11.7 Hz, 2H, H-8 and H-15), 4.57 (d, J_{AB} = 11.7 Hz, 2H, H-8' and H-15'), 4.49–4.43 (m, 1H, H-5), 4.40–4.35 (m, 2H, H-4 and H-6), 4.35–4.31 (m, 2H, H-1 and H-3), 4.14–4.09 (ddd, 2H, J_{XY} = 5.6, 1.3, 1.3 Hz, H-22 and H-22'), 3.98–4.02 (m, 1H, H-2); IR (thin film): 3384 (O-H) (br), 3064 (w), 3030 (w), 2970 (br), 2961 (w), 1497 (w), 1454 (w), 1377 (w), 1306 (w), 1207 (w), 1164 (s), 1139 (m), 1098 (s), 996 (s), 950 (m), 933 (m), 896 (m), 820 (w), 739 (m), 699 (m) cm^{-1}; LRMS (m/z): $[M+H]^+$ 411.2. These data are in good agreement with the literature values[9].

Synthetic protocol and characterisation data for 2-*O*-allyl-4,6-di-*O*-benzyl-*myo*-inositol (11)

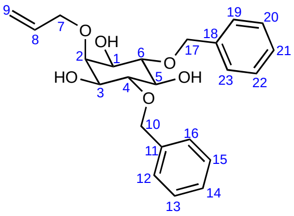

(11)

To a solution of **10** (893 mg, 2.18 mmol, 1.0 eq) in MeOH (2.2 mL) was added PTSA·H_2O (414 mg, 2.18 mmol, 1.0 eq). The reaction mixture was stirred at RT for 16 hours and quenched with Et_3N (304 µL, 2.18 mmol, 1.0 eq). The solution was concentrated *in vacuo*, and the residue obtained was purified by column chromatography over silica gel (Petroleum ether/EtOAc 8:2, 7:3, 6:4, 5:5, 4:6, 3:7, 2:8) to give **11** (822 mg, 94%) as a colourless oil: TLC (EtOAc): R_f = 0.34; ^{1}H NMR (500 MHz; $CDCl_3$): δ 7.40–7.35 (m, 8H, H-12, H-13, H-15, H-16, H-19, H-20, H-22 and H-23), 7.34–7.29 (m, 2H, H-14 and H-21), 5.94 (dddd, J = 17.2,

10.4, 5.5, 5.5 Hz, 1H, H-8), 5.28 (dddd, J_{XY} = 17.2, 1.8, 1.7, 1.7 Hz, 1H, H-9), 5.20 (dddd, J_{XY} = 10.4, 1.8, 1.4, 1.4 Hz, 1H, H-9'), 4.89 (d, J_{AB} = 11.4 Hz, 2H, H-10 and H-17), 4.86 (d, J_{AB} = 11.4 Hz, 2H, H-10' and H-17'), 4.33 (ddd, J_{XY} = 5.5, 1.7, 1.4 Hz, 2H, H-7 and H-7'), 3.90 (dd, J 2.8, 2.8 Hz, 1H, H-2), 3.64 (dd, J = 9.4, 9.4 Hz, 2H, H-4 and H-6), 3.57–3.50 (m, 3H, H-1, H-3 and H-5), 2.53 (d, J = 1.9 Hz, 1H, C(5)O*H*), 2.42 (s, 1H, C(1)O*H*), 2.41 (s, 1H, C(3)O*H*); ^{13}C NMR (126 MHz; $CDCl_3$): δ 138.6 (C-11, C-18), 135.0 (C-8), 128.8, 128.2, 128.1 (C-12 to C-16 and C-19 to C-23), 117.2 (C-9), 82.2 (C-4, C-6), 79.3 (C-2), 75.2 (C-10, C-17), 75.1 (C-5), 74.4 (C-7), 72.6 (C-1, C-3); IR (thin film): 3545 (O-H) (br), 3444 (O-H) (br), 3031 (w), 2981 (w), 2884 (w), 1497 (w), 1398 (w), 1364 (w), 1251 (w), 1211 (w), 1115 (s), 1061 (s), 1030 (s), 1002 (s), 930 (m), 732 (s), 697 (s) cm^{-1}; LRMS (m/z): $[M+Na]^+$ 423.2; HRMS (m/z): $[M+H]^+$ calcd. for $C_{23}H_{29}O_6$, 401.19587; found 401.19523. These data are in good agreement with the literature values[8,10].

Synthetic protocol and characterisation data for 2-*O*-allyl-1,3,4,5,6-penta-*O*-benzyl-*myo*-inositol (12)

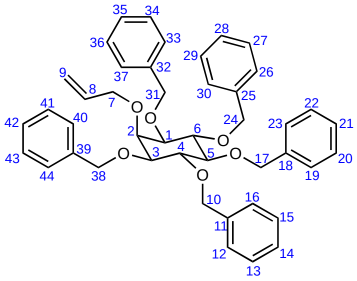

(**12**)

A solution of **11** (786 mg, 1.96 mmol, 1.0 eq) in DMF (16 mL) was cooled to 0 °C and NaH (471 mg, 11.8 mmol, 6.0 eq, 60% dispersion in mineral oil) was added po rtionwise. The reaction mixture was stirred at this temperature for 10 minutes, warmed to RT, and stirred for a further 30 minutes. The reaction mixture was then once again cooled to 0 °C. BnBr (1.38 mL, 11.6 mmol, 5.9 eq) was added dropwise. The reaction mixture was stirred at 0 °C for 10 minutes, warmed to RT, and stirred for a further 15 hours. After quenching with H_2O (8 mL), the aqueous phase was extracted with EtOAc (4 × 10 mL) and the combined organic layers were washed with H_2O (10 mL), and brine (10 mL), then dried ($MgSO_4$), filtered, and concentrated *in vacuo*. The residue obtained was purified by column chromatography over silica gel (Petroleum ether/EtOAc 9.5:0.5, 9:1, 8.5:1.5, 8:2, 8:3) to give **12** (1.04 g, 80%) as a colourless solid: mp: 102–103 °C; TLC (Petroleum ether:EtOAc, 7:3 v/v): R_f = 0.66; ^{1}H NMR (500 MHz; $CDCl_3$): δ 7.36–7.23 (m, 25H, H-12 to H-16, H-19 to

H-23, H-26 to H-30, H-33 to H-37 and H-40 to H-44), 5.98 (dddd, 1H, J = 17.4, 10.3, 6.0, 6.0 Hz, H-8), 5.30 (dddd, J_{XY} = 17.4, 1.8, 1.7, 1.7 Hz, 1H, H-9), 5.18 (dddd, J_{XY} = 10.3, 1.8, 1.4, 1.4 Hz, 1H, H-9'), 4.91 (d, J_{AB} = 10.6 Hz, 2H, H-10 and H-24), 4.87 (s, 2H, H-17), 4.82 (d, J_{AB} = 10.6 Hz, 2H, H-10' and H-24'), 4.68 (d, J_{AB} = 12.2 Hz, 2H, H-31 and H-38), 4.66 (d, J_{AB} = 12.2 Hz, 2H, H-31' and H-38'), 4.34 (ddd, J_{XY} 6.0, 1.7, 1.4 Hz, 2H, H-7 and H-7'), 4.03 (dd, J = 9.7, 9.4 Hz, 2H, H-4 and H-6), 3.97 (dd, J = 2.4, 2.4 Hz, 1H, H-2), 3.46 (dd, J = 9.4, 9.4 Hz, 1H, H-5), 3.33 (dd, J 9.7, 2.4 Hz, 2H, H-1 and H-3); ^{13}C NMR (126 MHz; $CDCl_3$): δ 139.00 (C-11, C-25), 138.97 (C-18), 138.5 (C-32, C-39), 135.9 (C-8), 128.53, 128.49, 128.46, 128.2, 128.0, 127.8, 127.6 (C-12 to C-16, C-19 to C-23, C-26 to C-30, C-33 to C-37 and C-40 to C-44), 117.0 (C-9), 83.8 (C-5), 81.8 (C-4, C-6), 80.9 (C-1, C-3), 76.1 (C-17), 76.0 (C-10, C-24), 74.0 (C-2), 73.6 (C-7), 72.9 (C-31, C-38); IR (thin film): 3065 (w), 3029 (w), 2921 (w), 2874 (w), 1497 (w), 1454 (w), 1358 (m), 1215 (w), 1159 (w), 1130 (m), 1090 (s), 1066 (s), 1039 (s), 1029 (s), 998 (m), 930 (w), 749 (m), 730 (s), 694 (s) cm^{-1}; LRMS (m/z): $[M+Na]^+$ 693.3; HRMS (m/z): $[M+H]^+$ calcd. for $C_{44}H_{47}O_6$, 671.33672; found 671.33641. These data are in good agreement with the literature values[10].

Synthetic protocol and characterisation data for 1,3,4,5,6-penta-*O*-benzyl-*myo*-inositol (13)

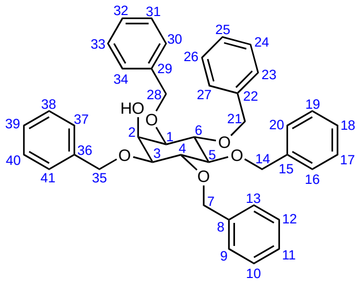

(**13**)

To a solution of **12** (820 mg, 1.22 mmol, 1.0 eq) in anhydrous MeOH/CH_2CH_2 (2.5:1.0 *v/v*, 8.8 mL) was added $PdCl_2$ (24 mg, 0.133 mol, 0.11 eq). The reaction mixture was stirred at RT for 18 hours, then diluted with CH_2Cl_2 (5 mL), filtered through a pad of Celite® and washed with CH_2Cl_2 (3 × 5 mL). The filtrate was concentrated *in vacuo*, and the residue obtained was purified by column chromatography over silica gel (Petroleum ether/EtOAc 9:1, 8:2, 7:3), to give **13** (739 mg, 96%) as a colourless solid: mp: 129–130 °C (lit.[11] 128–129 °C; lit.[12] 128–130 °C); TLC (Petroleum ether:EtOAc, 7:3 v/v): R_f = 0.43; ^{1}H NMR (400 MHz; $CDCl_3$): δ 7.37–7.24 (m, 25H, H-9 to H-13, H-16 to H-20, H-23 to H-27, H-30 to H-34 and H-37 to H-41),

4.93–4.82 (m, 6H, H-7, H-14 and H-21), 4.73 (d, J_{AB} = 12.3 Hz, 2H, H-28 and H-35), 4.70 (d, J_{AB} = 12.3 Hz, 2H, H-28' and H-35'), 4.22 (dd, J = 2.6, 2.6 Hz, 1H, H-2), 4.00 (dd, J = 9.5, 9.5 Hz, 2H, H-4 and H-6), 3.46 (dd, J = 9.5, 9.5 Hz, 1H, H-5), 3.39 (dd, J = 9.5, 2.6 Hz, 2H, H-1 and H-3), 2.45 (br s, 1H, C(2)O*H*); ^{13}C NMR (101 MHz; $CDCl_3$): δ 138.91 (C-8, C-22), 138.85 (C-15), 138.1 (C-29, C-36), 128.6, 128.5, 128.1, 128.02, 127.98, 127.71, 127.68 (C-9 to C-13, C-16 to C-20, C-23 to C-27, C-30 to C-34 and C-37 to C-41), 83.3 (C-5), 81.4 (C-4, C-6), 80.0 (C-1, C-3), 76.1 (C-7, C-14, C-21), 72.9 (C-28, C-35), 67.7 (C-2); IR (thin film): 3449 (O-H) (br), 3065 (w), 3032 (w), 2882 (w), 1497 (w), 1454 (m), 1358 (m), 1134 (m), 1086 (m), 1064 (s), 1030 (s), 914 (w), 753 (s), 726 (s), 695 (s) cm^{-1}; LRMS (m/z): $[M+Na]^+$ 653.3; HRMS (m/z): $[M+H]^+$ calcd. for $C_{41}H_{43}O_6$, 631.30542; found 631.30512. The data are in good agreement with the literature values[11–13].

Synthetic protocol and characterisation data for 2,3,4,5,6-penta-*O*-benzyl-1-azido-1-deoxy-*scyllo*-inositol (14)

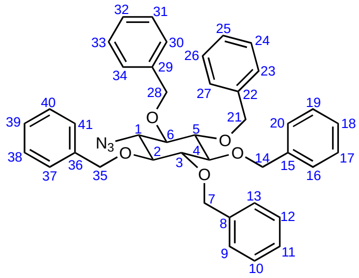

(**14**)

A solution of **13** (100 mg, 0.159 mmol, 1.0 eq) in anhydrous pyridine (1.4 mL) was cooled to 0 °C. Methanesulfonyl chloride (288 µL, 3.73 mmol, 23.5 eq) was added dropwise. The cloudy solution obtained was gradually warmed to RT and was stirred for a further 24 hours. The reaction was then quenched by the addition of H_2O (1 mL) and diluted with CH_2Cl_2 (2 mL). The aqueous phase was extracted with CH_2Cl_2 (3 × 2 mL) and the combined organic layers were washed with H_2O (2 × 1 mL), and brine (2 × 1 mL), then dried (Na_2SO_4), filtered, and concentrated *in vacuo*. Without any further purification the presumed crude mesyl inositol intermediate obtained was dissolved in anhydrous DMF (1.4 mL). NaN_3 (75 mg, 1.16 mmol, 7.3 eq) was added in one portion and the reaction mixture was stirred at 90 °C for 15 hours. After cooling to RT, H_2O (2 mL) was added. The aqueous phase was extracted with CH_2Cl_2 (3 × 2 mL), and the combined organic layers were washed with H_2O (2 × 1 mL), a saturated aqueous solution of $NaHCO_3$ (1 mL), and brine (2 × 1

mL), then dried ($MgSO_4$), filtered, and concentrated *in vacuo*. Purification by column chromatography over silica gel (Petroleum ether/EtOAc 9.5:0.5, 9:1, 8:2) yielded **14** (91 mg, 88%) as a yellow solid: mp: 96–97 °C (lit.[13] 95–96 °C); TLC (Petroleum ether:EtOAc, 4:1 v/v): R_f = 0.65; ^{1}H NMR (500 MHz; $CDCl_3$): δ 7.41–7.25 (m, 25H, H-9 to H-13, H-16 to H-20, H-23 to H-27, H-30 to H-34 and H-37 to H-41), 4.93–4.84 (m, 10H, H-7, H-14, H-21, H-28 and H-35), 3.62–3.52 (m, 10H, H-3, H-4 and H-5), 3.49 (dd, J = 10.1, 10.1 Hz, 1H, H-1), 3.36 (dd, J = 9.0, 9.0 Hz, 2H, H-2 and H-6); LRMS (m/z): 678.3 ($[M+Na]^+$; HRMS (m/z): $[M+Na]^+$ calcd. for $C_{41}H_{41}O_5N_3Na$, 678.29384; found 678.29340. The data are in good agreement with the literature values[13,14].

Synthetic protocol and characterisation data for 2,3,4,5,6-tetra-*O*-benzyl-*scyllo*-inosamine (15)

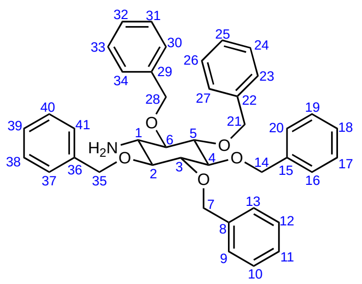

(15)

To a solution of **14** (2.00 g, 3.05 mmol, 1.0 eq) in THF (49 mL) was added PPh_3 (1.60 g, 6.10 mmol, 2.0 eq) and H_2O (2.1 mL). The solution was stirred at 40 °C for 30 hou rs, then cooled to RT. The solvents were concentrated *in vacuo* and the residue obtained was redissolved in CH_2Cl_2 (10 mL) and H_2O (5 mL) was added. The aqueous layer was extracted with CH_2Cl_2 (4 × 10 mL) and the combined organic layers were washed with H_2O (10 mL), and brine (10 mL), then dried (Na_2SO_4), filtered, and concentrated *in vacuo*. Purification by column chromatography over silica gel (Petroleum ether/EtOAc/Et_3N 9.5:0.45:0.05, 9:0.95:0.05, 8:1.95:0.05, 7:2.95:0.05, 6:3.95:0.05, 5:4.95:0.05) gave **15** (1.40 g, 73%) as a colourless glassy solid: mp: 122–123 °C; TLC (Petroleum ether:EtOAc, 5:5 v/v): R_f = 0.59; ^{1}H NMR (500 MHz; $CDCl_3$): δ 7.37–7.27 (m, 25H, H-9 to H-13, H-16 to H-20, H-23 to H-27, H-30 to H-34 and H-37 to H-41), 4.99 (d, J_{AB} = 10.9 Hz, 2H, H-28 and H-35), 4.93 (d, J_{AB} = 10.8 Hz, 2H, H-7 and H-21), 4.91 (s, 2H, H-14), 4.86 (d, J_{AB} = 10.8 Hz, 2H, H-7' and H-21'), 4.70 (d, J_{AB} = 10.9 Hz, 2H, H-28' and H-35'), 3.65–3.59 (m, 3H, H-3, H-4 and H-5), 3.40–3.31 (m, 2H, H-2 and H-6), 2.94 (dd, J = 9.9, 9.9 Hz, 1H, H-1); ^{13}C NMR (126 MHz; $CDCl_3$): δ

138.55 (C-15), 138.51, 138.48 (C-8, C-22, C-29, C-36), 128.7, 128.61 (C-10, C-12, C-24, C-26, C-31, C-33, C-38, C-40), 128.58 (C-17, C-19), 128.04, 128.02, 127.97, 127.94, 127.85, 127.82 (C-9, C-11, C-13, C-16, C-18, C-20, C-23, C-25, C-27, C-30, C-32, C-34, C-37, C-39, C-41), 84.5 (C-2, C-6), 83.7 (C-4), 83.1 (C-3, C-5), 76.2 (C-14), 76.0, 75.9 (C-7, C-21, C-28, C-35), 55.5 (C-1); IR (thin film): 3067 (N-H) (w), 3030 (N-H) (w), 2908 (w), 2870 (w), 1574 (w), 1496 (w), 1454 (w), 1351 (m), 1215 (w), 1151 (w), 1126 (m), 1094 (m), 1062 (s), 1040 (s), 1026 (s), 1011 (s), 906 (w), 830 (w), 751 (s), 694 (s), 661 (m), 624 (m) cm^{-1}; LRMS (m/z): $[M+H]^+$ 630.3; HRMS (m/z): $[M+H]^+$ calcd. for $C_{41}H_{44}NO_5$, 630.32140; found 630.32066; RP-HPLC (Method 1) t_R = 11.46 min, 95.25%.

Synthetic protocol and characterisation data for *scyllo*-inosamine hydrochloride (1)

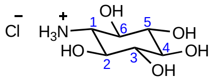

(1)

To a solution of the protected amine **15** (304 mg, 0.483 mmol, 1.0 eq) in CH_2Cl_2/MeOH/H_2O (3:2:1 *v/v/v*, 7.2 mL) was added 10% Pd/C (82 mg, 0.0773 mmol, 0.16 eq) and molecular biology grade concentrated HCl (200 µL) under $N_{2(g)}$. The flask was purged with 3 balloons of $H_{2(g)}$, and the suspension was stirred at RT under $H_{2(g)}$ for 24 hours. The reaction mixture was filtered through a glass microfiber filter and washed with MeOH/H_2O (4:1 *v/v*, 20 mL). The filtrate was partially concentrated *in vacuo*, then lyophilised. The colourless solid obtained was then dissolved in the minimal amount of H_2O (0.3 mL) and loaded onto a pre-washed DOWEX® 50WX8 cation exchange column (~3 mL of resin). The column was eluted with H_2O (10 mL), then 0.1 M HCl (10 mL) and 0.2 M HCl (5 mL). The fractions were combined and lyophilised to give the hydrochloride salt **1** (83 mg, 80%) as a colourless solid: decomp. 290–291 °C (lit.[14] 299 °C); ^1H NMR (400 MHz; D_2O): δ 3.49 (dd, 2H, J = 10.5, 9.0 Hz, H-2 and H-6), 3.42 (dd, J = 9.2, 9.0 Hz, 2H, H-3 and H-5), 3.35–3.29 (m, 1H, H-4), 3.04 (dd, J = 10.5, 10.5 Hz, 1H, H-1); ^{13}C NMR (126 MHz; D_2O): δ 74.2 (C-3, C-5), 73.1 (C-4), 70.1 (C-2, C-6), 55.7 (C-1); LRMS (m/z): $[M+H]^+$ 180.1; GC-MS (derivatised with TMSI) t_R = 16.81 min, 100%. The data are in good agreement with the literature values[13,14].

Synthetic protocol and characterisation data for (±)-4-*O*-(4-methoxybenzyl)-6-*O*-methyl-*myo*-inositol 1,3,5-orthoformate i.e. (±)-(24)

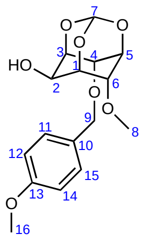

(±)-**(24)**

To a solution of **8** (19.7 g, 104 mmol, 1.0 eq) in anhydrous DMF (150 mL) was added NaH (4.24 g, 106 mmol, 1.02 eq, 60% dispersion in mi neral oil) portionwise over 10 minutes. The solution was stirred at RT for 30 minutes, then MeI (6.73 mL, 108 mmol, 1.04 eq) was added dropwise. After 16 hours stirring at RT, NaH (4.24 g, 106 mmol, 1.02 eq, 60% dispersion in mineral oil) was added portionwise and the reaction mixture was stirred for 1 hour. PMBCl (14.9 mL, 108 mmol, 1.04 eq) was then added dropwise over 10 minutes, and the solution was stirred at RT for 16 hours. The reaction was quenched by addition of H_2O (2 mL) and diluted in CH_2Cl_2 (20 mL). The aqueous layer was extracted with CH_2Cl_2 (3 × 10 mL). The combined organic phases were washed with H_2O (2 × 10 mL), brine (2 × 10 mL), and an aqueous solution of LiCl (10% *w/v*, 2 × 10 mL), then dried ($MgSO_4$), filtered, and concentrated *in vacuo*. The residue obtained was purified twice by column chromatography over silica gel (Petroleum ether/EtOAc 8:2, 7:3, 6:4, 5:5) to give (±)-**24** (1.56 g, 46%) as a yellow oil. This compound could not be purified further due to contamination by other regioisomers, which could only be separated in subsequent steps: TLC (Petroleum ether:EtOAc, 1:1 v/v): R_f = 0.27; ^{1}H NMR (400 MHz; $CDCl_3$): δ 7.28–7.22 (m, 2H, H-11 and H-15), 6.91–6.85 (m, 2H, H-12 and H-14), 5.45 (s, 1H, H-7), 4.59 (d, J_{AB} = 11.5 Hz, 1H, H-9), 4.51 (d, J_{AB} = 11.5 Hz, 1H, H-9'), 4.42–4.38 (m, 1H, H-5), 4.32–4.27 (m, 1H, H-4), 4.25–4.21 (m, 1H, H-1), 4.18–4.10 (m, 2H, H-6 and H-3), 4.09–4.06 (m, 1H, H-2), 3.80 (s, 3H, H-16), 3.44 (s, 3H, H-8), 3.09–2.97 (m, 1H, C(2)O*H*); ^{13}C NMR (101 MHz; $CDCl_3$): δ 159.6 (C-13), 129.8 (C-10), 129.4 (C-11, C-15), 114.1 (C-12, C-14), 103.5 (C-7), 76.0 (C-6), 73.3 (C-4), 73.1 (C-3), 72.7 (C-1), 71.5 (C-9), 67.7 (C-5), 61.5 (C-2), 57.7 (C-8), 55.4 (C-16); IR (thin film): 3476 (O-H) (br), 2980 (w), 2884 (w), 1734 (m), 1612 (w), 1514 (m), 1373 (w), 1302 (w), 1242 (s), 1144 (s) cm^{-1};

LRMS (m/z): $[M+Na]^+$ 347.1; HRMS (m/z): $[M+Na]^+$ calcd. for $C_{17}H_{24}O_6Na$, 347.1107; found 347.10932; RP-HPLC (Method 1) t_R = 10.62 min, 85.85%.

Synthetic protocol and characterisation data for (±)-2-*O*-allyl-4-*O*-(4-methoxybenzyl)-6-*O*-methyl-*myo*-inositol 1,3,5-orthoformate i.e. (±)-(16)

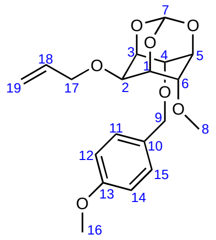

(±)-(**16**)

A solution of (±)-**24** (14.8 g, 45.6 mmol, 1.0 eq) in anhydrous DMF (250 mL) was cooled to 0 °C. NaH (4.56 g, 114 mmol, 2.5 eq, 60% dispersion in mineral oil) was added portionwise over 10 minutes. The reaction mixture was warmed to RT and stirred f or 2 hours. Allyl bromide (7.90 mL, 91.3 mmol, 2.0 eq) was subsequently added dropwise at 0 °C. The solution was warmed to RT and stirred for a further 17 hours. The reaction was quenched with H_2O (60 mL) and the aqueous layer was extracted with EtOAc (3 × 40 mL). The combined organic layers were washed with H_2O (2 × 30 mL), and brine (2 × 30 mL), then dried ($MgSO_4$), filtered, and concentrated *in vacuo*. Purification twice by column chromatography over silica gel (Petroleum ether/EtOAc 9:1, 8:2, 7:3, 6:4, 5:5) gave (±)-**16** (9.08 g, 55%) as a colourless oil. ^{1}H NMR analysis indicated that this compound was contaminated with ~3% of regioisomers: TLC (Petroleum ether/EtOAc, 5:5 v/v): R_f = 0.67; ^{1}H NMR (400 MHz; $CDCl_3$): δ 7.28–7.23 (m, 2H, H-11 and H-15), 6.92–6.86 (m, 2H, H-12 and H-14), 5.98 (dddd, J_{XY} = 17.1, 10.7, 5.6, 5.4 Hz, 1H, H-18), 5.50 (s, 1H, H-7), 5.36–5.28 (m, 1H, H-19), 5.24–5.18 (m, 1H, H-19'), 4.61 (d, J_{AB} = 11.7 Hz, 1H, H-9), 4.50 (d, J_{AB} = 11.7 Hz, 1H, H-9'), 4.41–4.37 (m, 1H, H-5), 4.36–4.31 (m, 1H, H-1), 4.31–4.27 (m, 1H, H-4), 4.24–4.19 (m, 1H, H-3), 4.17–4.10 (m, 3H, H-6 and H-17), 3.88–3.85 (m, 1H, H-2), 3.81 (s, 3H, H-16), 3.44 (s, 3H, H-8); ^{13}C NMR (101 MHz; $CDCl_3$): δ 159.6 (C-13), 134.8 (C-18), 129.9 (C-10), 129.4 (C-11, C-15), 117.8 (C-19), 114.1 (C-12, C-14), 103.4 (C-7), 76.2 (C-6), 73.8 (C-4), 71.7 (C-9), 70.8 (C-17), 70.7 (C-3), 70.4 (C-1), 68.0 (C-5), 67.6 (C-2), 57.6 (C-8), 55.4 (C-16); IR (thin film): 3564 (w), 2995 (w), 2916 (m), 2837 (w), 1612 (m), 1514 (s), 1464 (w), 1302 (m), 1250 (s), 1175

(s), 1138 (s) cm^{-1}; LRMS (m/z): $[M+Na]^+$ 387.2; HRMS (m/z): $[M+H]^+$ calcd. for $C_{19}H_{25}O_7$, 365.16003; found 365.15893; RP-HPLC (Method 1) t_R = 13.45 min, 97.11%.

Synthetic protocol and characterisation data for (±)-2-*O*-allyl-4-*O*-(4-methoxybenzyl)-6-*O*-methyl-*myo*-inositol-1,3-*O*-methylene i.e. (±)-(17)

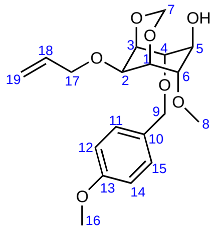

(±)-(17)

To a solution of (±)-**16** (10.3 g, 28.2 mmol, 1.0 eq) in anhydrous CH_2Cl_2 (140 mL) at 0 °C was added DIBAL-H (141 mL, 141 mmol, 5.0 eq, 1.0 M in hexane) dropwise over 10 minutes. After stirring at 0 °C for 10 minutes, the solution was warmed to RT and stirred for 24 hours. The reaction was then quenched by slow addition of a 1.0 M aqueous solution of Rochelle's salt (20 mL) and a saturated aqueous solution of NH_4Cl (20 mL) at 0 °C. After 30 minutes, the solution was warmed to RT, stirred for a further hour and then diluted in CH_2Cl_2 (20 mL). The aqueous phase was extracted with CH_2Cl_2 (3 × 20 mL), and the combined organic layers were washed with brine (15 mL), then dried ($MgSO_4$), filtered, and concentrated in *vacuo*. Purification by column chromatography over silica gel (Petroleum ether/EtOAc 7:3) afforded a yellowish oil which was cooled to −20 °C for 48 hours to give a colourless solid which was crystallised from Et_2O to yield (±)-**17** (3.05 g, 90%) as a colourless crystalline solid: mp: 63–65 °C; TLC (Petroleum ether:EtOAc, 1:1 v/v): R_f = 0.42; ^{1}H NMR (400 MHz; $CDCl_3$): δ 7.30–7.23 (m, 2H, H-11 and H-15), 6.92–6.84 (m, 2H, H-12 and H-14), 5.94 (dddd, J = 17.1, 10.5, 5.6, 5.4 Hz, 1H, H-18), 5.47 (d, J_{AB} = 5.0 Hz, 1H, H-7), 5.35–5.26 (m, 1H, H-19), 5.24–5.17 (m, 1H, H-19'), 4.66 (d, J_{AB} = 5.0 Hz, 1H, H-7'), 4.62 (d, J_{AB} = 11.8 Hz, 1H, H-9), 4.55 (d, J_{AB} = 11.8 Hz, 1H, H-9'), 4.41–4.37 (m, 1H, H-1), 4.33–4.29 (m, 1H, H-3), 4.12–4.07 (m, 2H, H-17), 4.06–4.03 (m, 1H, H-2), 3.97–3.93 (m, 1H, H-4), 3.88–3.83 (m, 1H, H-5), 3.81 (s, 3H, H-16), 3.79–3.76 (m, 1H, H-6), 3.46 (s, 3H, H-8), 2.91 (br s, 1H, C(5)O*H*); ^{13}C NMR (101 MHz; $CDCl_3$): δ 159.5 (C-13) 134.4 (C-18), 130.1 (C-10), 129.5 (C-11, C-15), 117.4 (C-19), 114.1 (C-12, C-14), 85.1 (C-7), 83.7 (C-6), 80.8 (C-4), 72.8 (C-3), 72.3 (C-

1), 72.0 (C-9), 70.4 (C-2), 69.83 (C-17), 69.77 (C-5), 58.3 (C-8), 55.4 (C-16); IR (thin film): 3545 (O-H) (br), 2933 (w), 2920 (w), 1611 (w), 1514 (m), 1408 (w), 1250 (m), 1184 (m), 1134 (s), 1094 (s), 1060 (s), 1028 (s) cm^{-1}; LRMS (m/z): $[M+Na]^+$ 389.2; HRMS (m/z): $[M+Na]^+$ calcd. for $C_{19}H_{26}O_7Na$, 389.15762; found 389.15637; RP-HPLC (Method 1) t_R = 11.62 min, 96.99%.

Synthetic protocol and characterisation data for (±)-2-*O*-allyl-5-*O*-benzyl-4-*O*-(4-methoxybenzyl)-6-*O*-methyl-*myo*-inositol-1,3-*O*-methylene i.e. (±)-(18)

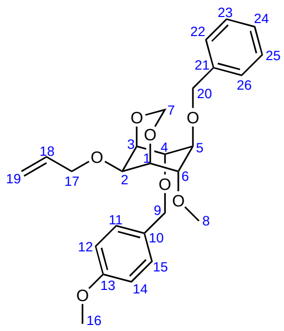

(±)-(**18**)

A solution of (±)-**17** (5.19 g, 14.2 mmol, 1.0 eq) in anhydrous DMF (40 mL) was cooled to 0 °C. Imidazole (675 mg, 9.91 mmol, 0.70 eq) was added to the solution, followed by NaH (1.70 g, 42.5 mmol, 3.0 eq, 60% dispersion in mineral oil). The reaction mixture was stirred at 0 °C for 10 minutes, warmed to RT, and stirred for a further 45 minutes. BnBr (5.05 mL, 42.5 mmol, 3.0 eq) was subsequently added dropwise at 0 °C. The reaction mixture was stirred for 25 minutes, warmed to RT, and stirred for 20 hours. The reaction was quenched with H_2O (25 ml) and diluted in EtOAc (25 mL). The aqueous phase was extracted with EtOAc (5 × 20 mL), and the combined organic layers were washed with brine (2 × 20 mL), H_2O (2 × 20 ml), and an aqueous solution of LiCl (10% *w/v*, 20 mL). The organic phase was then dried ($MgSO_4$), filtered, and concentrated in *vacuo*. Purification by column chromatography over silica gel (Petroleum ether/EtOAc 9:1, 8.5:1.5, 8.2) afforded (±)-**18** (6.03 g, 93%) as a thick colourless oil: TLC (Petroleum ether:EtOAc, 5:5 v/v): R_f = 0.75; ^{1}H NMR (400 MHz; $CDCl_3$): δ 7.29–7.14 (m, 7H, H-11, H-15 and H-22 to H-26), 6.82–6.76 (m, 2H, H-12 and H-14), 5.90 (dddd, J = 17.2, 10.5, 5.9, 5.5 Hz, 1H, H-18), 5.30–5.21 (m, 1H, H-19), 5.18–5.11 (m, 1H, H-19'), 5.05 (d, J_{AB} = 5.6 Hz, 1H, H-7), 4.78 (d, J_{AB} = 5.6 Hz, 1H, H-7'), 4.62 (s, 2H, H-20), 4.52 (d, J_{AB} = 11.4 Hz, 1H, H-9), 4.52 (d, J_{AB} = 11.4 Hz, 1H, H-9'), 4.16–4.12 (m, 1H, H-1), 4.11–4.03 (m, 3H, H-3

and H-17), 3.89–3.85 (m, 1H, H-4), 3.73 (s, 3H, H-16), 3.65–3.60 (m, 1H, H-6), 3.60–3.56 (m, 1H, H-2), 3.46 (dd, J = 5.9, 5.9 Hz, 1H, H-5), 3.35 (s, 3H, H-8); ^{13}C NMR (101 MHz; $CDCl_3$): δ 159.6 (C-13), 138.6 (C-21), 134.5 (C-18), 129.9 (C-10), 129.7 (C-11, C-15), 128.5 (C-23, C-25), 127.9 (C-22, C-26), 127.8 (C-24), 117.7 (C-19), 114.0 (C-12, C-14), 85.6 (C-7), 84.8 (C-6), 81.8 (C-4), 80.8 (C-5), 73.6 (C-20), 72.3 (C-1), 71.8 (C-3), 71.7 (C-9), 70.1 (C-2, C-17), 57.7 (C-8), 55.4 (C-16); IR (thin film): 2864 (w), 2836 (w), 1612 (w), 1514 (m), 1455 (w), 1366 (w), 1248 (m), 1176 (m), 1084 (s), 1029 (s), 1012 (s), 821 (m), 733 (s), 698 (s) cm^{-1}; LRMS (m/z): $[M+Na]^+$ 479.2; HRMS (m/z): $[M+Na]^+$ calcd. for $C_{26}H_{32}O_7Na$, 479.20402; found 479.20308; RP-HPLC (Method 1) t_R = 14.56 min, 94.32%.

Synthetic protocol and characterisation data for (±)-2-*O*-allyl-5-*O*-benzyl-6-*O*-methyl-*myo*-inositol i.e. (±)-(19)

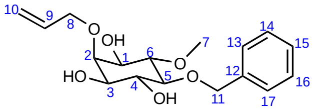

(±)-(19)

To a solution of (±)-**18** (832 mg, 1.82 mmol, 1.0 eq) in MeOH (10 mL) at 0 °C was added concentrated HCl (1.30 mL). The solution was warmed to RT and subsequently heated under reflux for 2 hours. After cooling to RT, the reaction was quenched with a saturated aqueous solution of $NaHCO_3$ until the pH reached 7. The volatile components were concentrated *in vacuo* and the residue obtained was purified by column chromatography over silica gel (Petroleum ether/EtOAc 3:7, 2:8, 1:9). The colourless solid obtained was then crystallised from EtOAc to give (±)-**19** (518 mg, 88%) as a colourless crystalline solid: mp: 141–143 °C; TLC (Petroleum ether:EtOAc, 1:1 v/v): R_f = 0.32; ^{1}H NMR (400 MHz; $CDCl_3$): δ 4.41–7.28 (m, 5H, H-13 to H-17), 5.93 (dddd, J = 17.2, 10.4, 5.6, 5.6 Hz, 1H, H-9), 5.30 (dddd, J_{XY} = 17.2, 1.9, 1.7, 1.5 Hz, 1H, H-10), 5.20 (dddd, J_{XY} = 10.4, 1.9, 1.6, 1.4 Hz, 1H, H-10'), 4.90 (d, J_{AB} = 11.4 Hz, 1H, H-11), 4.78 (d, J_{AB} = 11.4 Hz, 1H, H-11'), 4.36 (dddd, J_{XY} = 12.9, 5.6, 1.7, 1.6 Hz, 1H, H-8), 4.31 (dddd, J_{XY} = 12.9, 5.6, 1.5, 1.4 Hz, 1H, H-8'), 3.92 (dd, J = 2.6, 2.6 Hz, 1H, H-2), 3.82–3.75 (m, H-41H,), 3.66 (s, 3H, H-7), 3.54–3.48 (m, 1H, H-1 or H-3), 3.48–3.40 (m, 2H, H-1 or H-3 and H-6), 3.23 (dd, J = 9.0, 9.0 Hz, 1H, H-5), 2.45 (d, J = 1.6 Hz, 1H, C(4)O*H*), 2.42 (d, J = 5.5 Hz, 1H, C(1)O*H* or C(3)O*H*), 2.38 (d, J = 6.5 Hz, 1H, C(1)O*H* or

C(3)O*H*); ^{13}C NMR (101 MHz; $CDCl_3$): δ 138.7 (C-12), 135.0 (C-9), 128.7 (C-14, C-16), 128.1 (C-13, C-17), 128.0 (C-15), 117.1 (C-10), 83.9 (C-6), 83.0 (C-5), 78.7 (C-2), 75.1 (C-11), 74.4 (C-8), 74.1 (C-4), 72.9 (C-1 or C-3), 72.6 (C-1 or C-3), 61.4 (C-7); IR (thin film): 3360 (O-H) (br), 2918 (w), 1498 (w), 1362 (w), 1192 (m), 1156 (s), 1053 (s), 1033 (s), 923 (m), 913 (m), 733 (s), 695 (s) cm^{-1}; LRMS (m/z): [M+Na]$^+$ 347.1; HRMS (m/z): [M+Na]$^+$ calcd. for $C_{17}H_{24}O_6Na$, 347.14651; found 347.14600; RP-HPLC (Method 1) t_R = 9.09 min, 98.22%; Chiral HPLC (Heptane/IPA 90:10, 0.8 mL.min^{-1}, 254 nm) t_R = 19.59 min, 50.49% (+)-1L-**19**, t_R = 22.39 min, 49.51% (−)-1D-**19**.

Synthetic protocol and characterisation data for (+)-1D-1-*O*-((*S*)-α-methoxyphenylacetoxy)-2-*O*-allyl-3,4-*O*-(L-1,7,7-trimethyl[2.2.1]bicyclohept-2-ylidene)-5-*O*-benzyl-6-*O*-methyl-*myo*-inositol i.e. (+)-1D-(25)

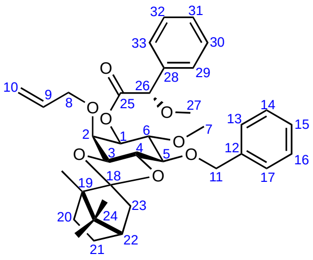

(+)-1D-**(25)**

To a solution of (±)-**19** (2.00 g, 6.17 mmol, 1.0 eq) in anhydrous CH_2Cl_2 (39 mL) were added 1-(*S*)-(−)-camphor dimethylacetal (3.67 g, 18.5 mmol, 3.0 eq) and PTSA·H_2O (59 mg, 0.308 mmol, 0.050 eq). The solution was heated under reflux for 24 hours, allowed to cool to RT and quenched with Et_3N (43 μL, 0.308 mmol, 0.050 eq). The solvent was removed *in vacuo*. The residue obtained, containing a mixture the four diastereoisomers **20a–d**, was then purified and resolved by column chromatography over silica gel (Petroleum ether/EtOAc 9.7:0.3, 9.6:0.4, 9.5:0.5, 9.4:0.6, 9.3:0.7, 9.2:0.8, 9.1:0.9, 9:1, 8.8:1.2, 8.5:1.5, 8:2, 7.5:2.5, 7:3, 6.5:3.5, 6:4) to give a mixture of diastereoisomers of **20b–d** (1.90 g, 67%) and (−)-1D-**20a** (466 mg, 16%) as colourless oils.

Deprotection of a small sample of (−)-1D-**20a**, with acetyl chloride (2.48 mmol, 0.60 eq) in MeOH/CH_2Cl_2 (1.0:1.8 *v/v*) for 21 hours, as described below, and analysis of the triol (−)-**19** obtained by

chiral HPLC revealed that this material was only partially resolved: Chiral HPLC (Heptane/IPA 90:10, 0.8 mL.min^{-1}, 254 nm) t_R = 19.74 min, 72.40% (+)-1L-**19**, t_R = 22.60 min, 27.60% (−)-1D-**19**, 45% *e.e.*

Intermediate (−)-1D-**20a** was thus further resolved, and its *e.e.* was simultaneous determined, using α-methoxyphenylacetic acid: To a solution of (−)-1D-**20a** (15 mg, 0.0327 mmol, 1.0 eq) in CH_2Cl_2 (0.30 mL) were added (*S*)-(+)-α-methoxyphenylacetic acid (14 mg, 0.0818 mmol, 2.5 eq), 1-ethyl-3-(3-dimethylaminopropyl)carbodiimide (14 mg, 0.0883 mmol, 2.7 eq) and 4-DMAP (2 mg, 0.0164 mmol, 0.50 eq). After 42 hours stirring at RT, the reaction mixture was diluted with CH_2Cl_2 (3 mL), and the organic layer was washed with a saturated aqueous solution of $NaHCO_3$ (2 × 5 mL), and brine (5 mL), then dried ($MgSO_4$), filtered, and concentrated *in vacuo*. Purification by column chromatography over silica gel (Petroleum ether/EtOAc 9:1, 8:2) yielded (+)-1D-**25** (18 mg, 90%) as a colourless oil: TLC (Petroleum ether:EtOAc, 4:1 v/v): R_f = 0.43; $[\alpha]_D^{25}$ = +27.2 (*c* 1.0 in $CHCl_3$); ^{1}H NMR (500 MHz; $CDCl_3$): δ 7.51–7.45 (m, 2H, H-29 and H-33), 7.40–7.24 (m, 8H, H-13 to H-17 and H-30 to H-32), 5.46 (dddd, J = 17.4, 10.6, 5.5, 5.4 Hz, 1H, H-9), 5.08–5.02 (m, 1H, H-10), 5.01–4.95 (m, 1H, H-10'), 4.89 (d, J_{AB} = 11.8 Hz, 1H, H-11), 4.83 (dd, J = 9.9, 2.8 Hz, 1H, H-1), 4.82 (s, 1H, H-26), 4.72 (d, J_{AB} = 11.8 Hz, 1H, H-11'), 3.97–3.85 (m, 3H, H-8, H-2 and H-4), 3.63–3.55 (m, 2H, H-8' and H-6), 3.52 (s, 3H, H-7), 3.54–4.48 (m, 1H, H-5), 3.44 (s, 3H, H-27), 3.22 (dd, J = 9.8 Hz, 1H, 1.0, H-3), 2.11 (dt, J = 13.3, 3.5 Hz, 1H, H_{eq}-23), 1.84–1.75 (m, 1H, H_{eq}-20), 1.74–1.64 (m, 2H, H-22 and H_{eq}-21), 1.40 (d, J = 13.3 Hz, 1H, H_{ax}-23), 1.32 (td, J = 12.2, 4.2 Hz, 1H, H_{ax}-20), 1.20–1.11 (m, 1H, H_{ax}-21), 1.03 (s, 3H, C(24)CH_3), 0.84 (s, 3H, C(19)CH_3 or C(24)CH_3), 0.82 (s, 3H, C(19)CH_3 or C(24)CH_3); ^{13}C NMR (126 MHz; $CDCl_3$): δ 170.2 (C-25), 138.8 (C-12), 136.2 (C-28), 134.6 (C-9), 128.9, 128.8, 128.4, 127.9, 127.6, 127.4 (C-13 to C-17 and C-29 to C-33), 120.8 (C-18), 116.8 (C-10), 82.6 (C-26), 81.9 (C-6), 80.4 (C-5), 77.2 (C-4), 76.0 (C-3), 75.0 (C-1), 72.9 (C-8), 72.6 (C-11), 71.9 (C-2), 61.5 (C-7), 57.6 (C-27), 53.0 (C-19), 48.4 (C-24), 46.2 (C-23), 45.0 (C-22), 28.9 (C-20), 26.7 (C-21), 20.4 (C(24)CH_3), 20.3 (C(24)CH_3), 9.7 (C(19)CH_3); IR (thin film): 2975 (m), 2932 (m), 2871 (m), 1754 (C=O) (m), 1496 (w), 1454 (m), 1371 (w), 1349 (w), 1310 (w), 1262 (w), 1202 (m), 1165 (m), 1153 (m), 1115 (s), 1089 (s), 1067 (s), 1046 (m), 1028 (m), 1016 (m), 926 (m), 844 (w), 823 (w), 736 (m), 697 (s) cm^{-1}; LRMS (m/z): $[M+H]^+$ 607.3; HRMS (m/z): $[M+H]^+$ calcd. for $C_{36}H_{47}O_8$, 607.32654; found 607.32603.

Synthetic protocols and characterisation data for (−)-1D-1-*O*-((*R*)-α-methoxyphenylacetoxy)-2-*O*-allyl-3,4-*O*-(L-1,7,7-trimethyl[2.2.1]bicyclohept-2-ylidene)-5-*O*-benzyl-6-*O*-methyl-*myo*-inositol i.e. (−)-1D-(25)

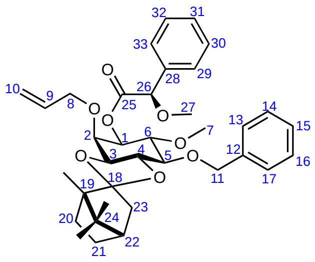

(-)-1D-**(25)**

The partially resolved intermediate (−)-1D-**20a** obtained as described above, was further resolved, and its *e.e.* determined, using α-methoxyphenylacetic acid: To a solution of (−)-1D-**20a** (15 mg, 0.0327 mmol, 1.0 eq) in CH_2Cl_2 (0.30 mL) were added (*R*)-(−)-α-methoxy phenylacetic acid (14 mg, 0.0818 mmol, 2.5 eq), 1-ethyl-3-(3-dimethylaminopropyl)carbodiimide (14 mg, 0.0883 mmol, 2.7 eq) and 4-DMAP (2 mg, 0.0164 mmol, 0.50 eq). After 42 hours stirring at RT, the reaction mixture was diluted with CH_2Cl_2 (3 mL), and the organic layer was washed with a saturated aqueous solution of $NaHCO_3$ (2 × 5 mL), and brine (5 mL), then dried ($MgSO_4$), filtered, and concentrated *in vacuo*. Purification by column chromatography over silica gel (Petroleum ether/EtOAc 9:1, 8:2) yielded (−)-1D-**25** (7 mg, 57%) as a colourless oil: TLC (Petroleum ether:EtOAc, 4:1, v/v): R_f = 0.43; $[\alpha]_D^{25}$ = −29.6 (*c* 0.56, $CHCl_3$); ^{1}H NMR (400 MHz; $CDCl_3$): δ 7.50–7.45 (m, 2H, H-29 and H-33), 7.40–7.23 (m, 8H, H-13 to H-17 and H-30 to H-32), 5.80 (dddd, J = 17.4, 10.6, 5.5, 5.4 Hz, 1H, H-9), 5.32–5.23 (m, 1H, H-10), 5.19–5.12 (m, 1H, H-10’), 4.86 (d, J_{AB} = 11.8 Hz, 1H, H-11), 4.81 (s, 1H, H-26), 4.75 (dd, J = 9.0, 3.3 Hz, 1H, H-1), 4.68 (d, J_{AB} = 11.8 Hz, 1H, H-11’), 4.27–4.20 (m, 1H, H-8), 4.20–4.16 (m, 1H, H-2), 4.04–3.97 (m, 1H, H-8’), 3.98–3.91 (m, 1H, H-4), 3.48–3.44 (m, 2H, H-5 and H-6), 3.43 (s, 3H, H-27), 3.25 (dd, J = 9.9, 1.0 Hz, 1H, H-3), 3.05 (s, 3H, H-7), 2.12 (dt, J = 13.3, 3.5 Hz, 1H, H_{eq}-23), 1.89–1.78 (m, 1H, H_{eq}-20), 1.77–1.64 (m, 2H, H-22 and H_{eq}-21), 1.43 (d, J = 13.3 Hz, 1H, H_{ax}-23), 1.36 (td, J = 12.2, 4.2 Hz, 1H, H_{ax}-20), 1.23–1.14 (m, 1H, H_{ax}-21), 1.05 (s, 3H, C(24)CH_3), 0.85 (s, 6H, C(19)CH_3 and C(24)CH_3); ^{13}C NMR (101 MHz; $CDCl_3$): δ 170.3 (C-25), 138.8 (C-12), 136.1 (C-28), 134.7 (C-9), 129.0, 128.8, 128.4, 127.9, 127.7, 127.6 (C-13 to C-17 and C-29 to C-33), 120.8 (C-18), 116.9 (C-10), 82.8 (C-26), 81.9 (C-6), 80.7 (C-5), 77.4 (C-4), 76.2 (C-3), 75.2 (C-1), 73.0 (C-8), 72.7 (C-11), 72.1 (C-2),

61.2 (C-7), 57.4 (C-27), 53.1 (C-19), 48.4 (C-24), 46.1 (C-23), 45.1 (C-22), 29.0 (C-20), 26.8 (C-21), 20.4 (C(24)CH_3), 20.3 (C(24)CH_3), 9.76 (C(19)CH_3); IR (thin film): 2930 (br), 2874 (w), 1753 (w), 1735 (C=O) (w), 1454 (w), 1261 (m), 1202 (m), 1167 (m), 1109 (s), 1088 (s), 1066 (s), 1028 (s), 1015 (s), 926 (w), 737 (m), 697 (s) cm^{-1}; LRMS (m/z): $[M+H]^+$ 607.3; HRMS (m/z): $[M+Na]^+$ calcd. for $C_{36}H_{46}O_8Na$, 629.30849; found 629.30679; NP-HPLC (Method 3) t_R = 5.69 min, 100%.

Synthetic protocol and characterisation data for (+)-1D-1-*O*-((*S*)-acetylmandelyl)-3,4-*O*-(L-1,7,7-trimethylbicyclo[2.2.1]hept-2-ylidene)-5-*O*-benzyl-6-*O*-methyl-*myo*-inositol i.e. (+)-1D-(21) & (−)-1L-1-*O*-((*R*)-acetylmandelyl)-2-*O*-allyl-3,4-*O*-(L-1,7,7-trimethylbicyclo[2.2.1]hept-2-ylidene)-5-*O*-benzyl-6-*O*-methyl-*myo*-inositol i.e. (−)-1L-(21)

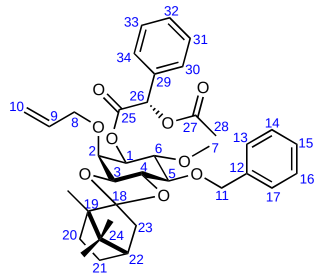
(+)-1D-(**21**)

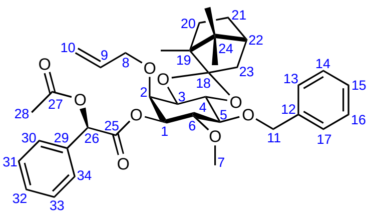
(−)-1L-(**21**)

To a solution of (±)-**19** (2.00 g, 6.17 mmol, 1.0 eq) in anhydrous CH_2Cl_2 (39 mL) were added 1-(*S*)-(−)-camphor dimethylacetal (3.67 g, 18.5 mmol, 3.0 eq) and PTSA·H_2O (59 mg, 0.308 mmol, 0.050 eq). The solution was heated under reflux for 24 hours, allowed to cool to RT and quenched with Et_3N (43 μL, 0.308 mmol, 0.050 eq). The solvent was r emoved *in vacuo*. The residue obtained, containing a mixture of four diastereoisomers, was then purified and resolved by column chromatography over silica gel (Petroleum ether/EtOAc 9.7:0.3, 9.6:0.4, 9.5:0.5, 9.4:0.6, 9.3:0.7, 9.2:0.8, 9.1:0.9, 9:1, 8.8:1.2, 8.5:1.5, 8:2, 7.5:2.5, 7:3, 6.5:3.5, 6:4) to give a mixture of diastereoisomers of **20b–d** (1.90 g, 67%) and partially resolved (−)-1D-**20a** (466 mg, 16%) as colourless oils. Intermediate (−)-1D-**20a** was then further resolved using (*S*)-(+)-*O*-acetylmandelic acid as a chiral auxiliary: To a solution of (−)-1D-**20a** (450 mg, 0.981 mmol, 1.0 eq) in CH_2Cl_2 (9.3 mL) were added (*S*)-(+)-*O*-acetylmandelic acid (381 mg, 1.96 mmol, 2.0 eq), 1-ethyl-3-(3-dimethylaminopropyl)carbodiimide (508 mg, 2.65 mmol, 2.7 eq) and 4-DMAP (60 mg, 0.491 mmol, 0.5 eq). After 17 hours stirring at RT the yellow solution was diluted with CH_2Cl_2 (4 mL), and washed with a

saturated aqueous solution of $NaHCO_3$ (2 × 10 mL). The aqueous layer was then back-extracted with CH_2Cl_2 (2 × 10 mL). The combined organic layers were dried ($MgSO_4$), filtered, and concentrated *in vacuo*. Purification and further resolution by column chromatography over silica gel (Petroleum ether/EtOAc 9.9:0.1, 9.8:0.2, 9.7:0.3, 9.6:0.4, 9.5:0.5, 9:1, 8.5:1.5, 8:2) yielded (+)-1D-**21** (458 mg, 74%) as an amorphous, colourless foam: TLC (Petroleum ether:EtOAc, 4:1 v/v): R_f = 0.43; $[\alpha]_D^{25}$ = +11.9 (*c* 2.0, CH_3Cl); ^{1}H NMR (400 MHz; $CDCl_3$): δ 7.54–7.48 (m, 2H, H-30 and H-34), 7.44–7.26 (m, 8H, H-13 to H-17 and H-31 to H-33), 5.96 (s, 1H, H-26), 5.49 (dddd, J = 17.2, 10.3, 5.5, 5.4 Hz, 1H, H-9), 5.10–5.03 (m, 1H, H-10), 5.01–4.95 (m, 1H, H-10'), 4.89 (d, J_{AB} = 12.0 Hz, 1H, H-11), 4.86 (dd, J = 9.2, 3.2 Hz, 1H, H-1), 4.73 (d, J_{AB} = 12.0 Hz, 1H, H-11'), 3.98–3.87 (m, 3H, H-8, H-4 and H-2), 3.70–3.63 (m, 1H, H-8'), 3.60 (dd, J = 9.2, 9.2 Hz, 1H, H-6), 3.57 (s, 3H, H-7), 3.50 (dd, J = 9.2, 8.6 Hz, 1H, H-5), 3.20 (dd, J = 9.8, 1.4 Hz, 1H, H-3), 2.19 (s, 3H, H-28), 2.10 (dt, J = 13.6, 3.8 Hz, 1H, H_{eq}-23), 1.82–1.74 (m, 1H, H_{eq}-20), 1.74–1.63 (m, 2H, H-22 and H_{eq}-21), 1.39 (d, J = 13.6 Hz, 1H, H_{ax}-23), 1.29 (td, J = 12.0, 4.3 Hz, 1H, H_{ax}-20), 1.19–1.10 (m, 1H, H_{ax}-21), 1.04 (s, 3H, C(19)CH_3), 0.83 (s, 3H, C(24)CH_3), 0.82 (s, 3H, C(24)CH_3); ^{13}C NMR (101 MHz; $CDCl_3$): δ 170.3 (C-25), 168.4 (C-27), 138.8 (C-12), 134.6 (C-9), 133.8 (C-29), 129.4, 128.9, 128.4, 127.9, 127.6 (C-13 to C-17 and C-30 to C-34), 120.8 (C-18), 116.6 (C-10), 82.1 (C-6), 80.4 (C-5), 77.4 (C-4), 76.1 (C-3), 75.4 (C-1), 74.6 (C-26), 72.9 (C-8), 72.7 (C-11), 72.0 (C-2), 61.7 (C-7), 53.1 (C-19), 48.4 (C-24), 46.2 (C-23), 45.1 (C-22), 29.0 (C-20), 26.7 (C-21), 20.8 (C-28), 20.4 (C(24)CH_3), 20.3 (C(19)CH_3), 9.69 (C(24)CH_3); IR (thin film): 2934 (br), 2873 (w), 2360 (w), 1749 (C=O) (s), 1454 (w), 1373 (m), 1263 (m), 1229 (s), 1206 (s), 1166 (m), 1110 (s), 1087 (s), 1064 (s), 1047 (s), 918 (m), 734 (s), 696 (s) cm^{-1}; LRMS (m/z): $[M+H]^+$ 635.3; HRMS (m/z): $[M+H]^+$ calcd. for $C_{37}H_{47}O_9$, 635.32146; found 635.3209; NP-HPLC (Method 3) t_R = 6.56 min, 99.73%.

Acetyl chloride (177 µL, 2.48 mmol, 0.60 eq) was added to a solution of the diastereoisomeric mixture **20b–d** (1.90 g, 4.14 mmol, 1.0 eq) in MeOH/CH_2Cl_2 (1.0:1.8 *v/v*, 27 mL). After stirring at RT for 21 hours, the reaction was quenched with Et_3N (346 µL, 2.48 mmol, 0.60 eq) and the solvents were removed *in vacuo*. The off-white solid obtained was then crystallised from EtOAc to give triol (+)-**19** (1.34 g, 94%) enriched in the opposite enantiomer.

Similarly therefore, from enriched (+)-**19** or racemic (±)-**19** (1.14 g, 3.51 mmol, 1.0 eq), 1-(*R*)-(+)-camphor dimethylacetal (3.48 g, 17.6 mmol, 5.0 eq) and PTSA·H_2O (33 mg, 0.176 mmol, 0.050 eq) in CH_2Cl_2 (18 mL), (+)-1L-**20c** (175 mg, 11% from racemic (±)-**19**, 589 mg, 37% from enriched (+)-**19**) was obtained as a colourless oil. Reaction of (+)-1L-**20c** (170 mg, 0.371 mmol, 1.0 eq) with (*R*)-(−)-*O*-acetylmandelic acid (144 mg, 0.741 mmol, 2.0 eq), 1-ethyl-3-(3-dimethylaminopropyl)carbodiimide (192 mg, 1.00 mmol, 2.7 eq) and 4-DMAP (23 mg, 0.185 mmol, 0.50 eq) yielded (−)-1L-**21** (191 mg, 74–81%) as a colourless oil: $[\alpha]_D^{25}$ = −11.2 (*c* 1.0, CH_3Cl). All other data (R_f, ^{1}H NMR, ^{13}C NMR, IR, LRMS, HRMS, HPLC) match those of (+)-1D-**21**.

Synthetic protocol and characterisation data for (+)-1D-1-*O*-((*S*)-acetylmandelyl)-2-*O*-allyl-5-*O*-benzyl-6-*O*-methyl-*myo*-inositol i.e. (+)-1D-(26); (−)-1D-1-*O*-((*S*)-2-hydroxy-2-phenylacetoxy)-2-*O*-allyl-5-*O*-benzyl-6-*O*-methyl-*myo*-inositol i.e. (−)-1D-(27) & (−)-1D-2-*O*-allyl-5-*O*-benzyl-6-*O*-methyl-*myo*-inositol i.e. (−)-1D-(19)

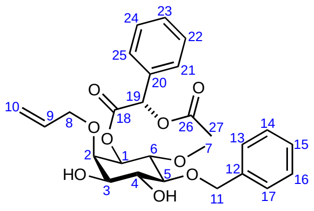

(+)-1D-(**26**)

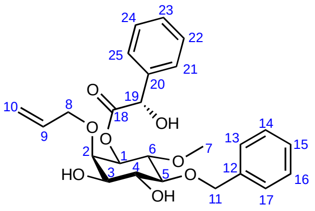

(−)-1D-(**27**)

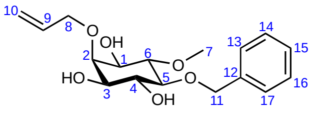

(−)-1D-(**19**)

To a solution of (+)-1D-**21** (30 mg, 0.0473 mmol, 1.0 eq) in MeOH/CH_2Cl_2 (1:2 *v/v*) was added acetyl chloride (2 µL, 0.0284 mmol, 0.60 eq). The solution was stirred at RT for 19 hours, then neutralised with Et_3N (4 µL, 0.0284 mmol, 0.60 eq) and the so lvents were concentrated *in vacuo*. Purification by column chromatography over silica gel (Petroleum eth er/EtOAc, 5:5, 4:6, 3:7, 2:8) afforded the expected product (+)-1D-**26** (4 mg, 17%) as a colourless gum, but also the deacetylated product (−)-1D-**27** (11 mg, 32%), and the fully deprotected product (−)-1D-**19** (6 mg, 37%) as colourless solids: (+)-1D-**26**: TLC (Petroleum ether:EtOAc, 1:1 v/v): R_f = 0.22; $[\alpha]_D^{25}$ = +17.7 (*c* 0.27, CH_3Cl); ^{1}H NMR (400 MHz; $CDCl_3$): δ 7.54–7.48 (m, 2H, H-21, H-25), 7.43–7.27 (m, 8H, H-22 to H-24 and H-13 to H-17), 5.97 (s, 1H, H-19), 5.57 (dddd, J = 17.1, 10.5, 5.7, 5.5 Hz, 1H, H-9), 5.02–4.94 (m, 2H, H-10 and H-10'), 4.92 (d, J_{AB} = 11.4 Hz, 1H, H-11), 4.89 (dd, J = 9.9, 2.6 Hz, 1H, H-1), 4.73 (d, J_{AB} = 11.4 Hz, 1H, H-11'), 3.70 (ddd, J = 9.3, 9.3, 1.5 Hz, 1H, H-4),

3.68–3.57 (m, 7H, H-7, H-6, H-2, H-8 and H-8’), 3.42 (ddd, J = 9.3, 6.8, 2.9 Hz, 1H, H-3), 3.25 (dd, J = 9.3, 9.3 Hz, 1H, H-5), 2.39 (d, J = 1.5 Hz, 1H, C(4)OH), 2.22–2.19 (m, 4H, C(3)O*H* and H-27); ^{13}C NMR (101 MHz; $CDCl_3$): δ 170.4 (C-18), 168.4 (C-26), 138.7 (C-12) 134.6 (C-9), 133.6 (C-20), 129.7, 129.1, 128.7, 128.2, 128.1, 128.0 (C-13 to C-17 and C-21 to C-25), 116.8 (C-10), 82.6 (C-5), 81.4 (C-6), 77.4 (C-2), 75.4 (C-11), 75.3 (C-1), 74.7 (C-19), 73.9 (C-8), 73.5 (C-4), 71.9 (C-3), 61.3 (C-7), 20.8 (C-27); IR (thin film): 3426 (O-H) (br), 2930 (w), 1744 (C=O) (s), 1497 (w), 1455 (w), 1372 (m), 1229 (s), 1210 (s), 1180 (m), 1118 (m), 1048 (s), 924 (m), 738 (m), 698 (s) cm^{-1}; LRMS (m/z): $[M+Na]^+$ 523.2; HRMS (m/z): $[M+Na]^+$ calcd. for $C_{27}H_{32}O_9Na$, 523.19385; found 523.1936; NP-HPLC (Method 3) t_R = 13.21 min, 88.75%; (−)-1D-**27**: mp: 145–146 °C; TLC (Petroleum ether:EtOAc, 1:1 v/v): R_f = 0.11; $[\alpha]_D^{25}$ = −5.16 (c 0.95, $CHCl_3$); ^{1}H NMR (400 MHz; $CDCl_3$): δ 7.46–7.42 (m, 2H, H-21, H-25), 7.40–7.28 (m, 8H, H-22 to H-24 and H-13 to H-17), 5.47 (dddd, J = 17.1, 10.5, 5.9, 5.5 Hz, 1H, H-9), 5.21 (d, J = 5.1 Hz, 1H, H-19), 5.00–4.95 (m, 1H, H-10), 4.95–4.88 (m, 1H, H-10’), 4.90 (d, J_{AB} = 11.3 Hz, 1H, H-11), 4.86 (dd, J = 10.3, 2.6 Hz, 1H, H-1), 4.76 (d, J_{AB} = 11.3 Hz, 1H, H-11’), 3.70 (dd, J = 9.4, 9.4 Hz, 1H, H-4), 3.67–3.60 (m, 2H, H-6 and H-2), 3.58 (s, 3H, H-7), 3.52 (d, J = 5.1 Hz, 1H, C(19)O*H*), 3.45–3.38 (m, 3H, H-3, H-8 and H-8’), 3.25 (dd, J = 9.4, 9.4 Hz, 1H, H-5), 2.43 (d, J = 1.5 Hz, 1H, C(4)O*H*), 2.22 (d, J = 7.1 Hz, 1H, C(3)O*H*); ^{13}C NMR (101 MHz; $CDCl_3$): δ 173.3 (C-18), 138.6 (C-12), 138.0 (C-20), 134.5 (C-9), 129.0, 128.9, 128.7, 128.08, 128.05 (C-13 to C-17 and C-22 to C-24), 127.0 (C-21, C-25), 116.8 (C-10), 82.7 (C-5), 81.2 (C-6), 77.3 (C-2), 76.1 (C-1), 75.4 (C-11), 73.70 (C-8), 73.65 (C-4), 73.4 (C-19), 71.8 (C-3), 61.4 (C-7); IR (thin film): 3395 (O-H) (br), 2934 (w), 2874 (w), 1739 (m), 1714 (w), 1453 (w), 1421 (w), 1262 (w), 1206 (w), 1182 (w), 1156 (m), 1143 (m), 1123 (m), 1053 (s), 1030 (s), 933 (m), 735 (m), 696 (s) cm^{-1}; LRMS (m/z): $[M+Na]^+$ 481.2; HRMS (m/z): $[M+Na]^+$ calcd. for $C_{25}H_{30}O_8Na$, 481.18329; found 481.1831; NP-HPLC (Method 3) t_R = 11.36 min, 99.51%. The data for (−)-1D-**19** are identical to those reported below.

Synthetic protocol and characterisation data for (−)-1D-2-*O*-allyl-3,4-*O*-(L-1,7,7-trimethylbicyclo-[2.2.1]hept-2-ylidene)-5-*O*-benzyl-6-*O*-methyl-*myo* inositol i.e. (−)-1D-(20a) & (+)-1L-2-*O*-allyl-3,4-*O*-(L-1,7,7-trimethylbicyclo[2.2.1]hept-2-ylidene)-4-*O*-methyl-5-*O*-benzyl-*myo* inositol i.e. (+)-1L-(20c)

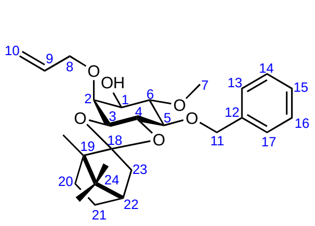

(−)-1D-**(20a)**

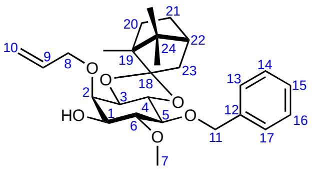

(+)-1L-**(20c)**

Ground solid NaOH (135 mg, 3.37 mmol, 8.1 eq) was added to a solution of (+)-1D-**21** (263 mg, 0.414 mmol, 1.0 eq) in MeOH (35 mL). The reaction mixture was heated under reflux for 30 minutes, then allowed to cool to RT and neutralised by slow addition of solid CO_2. The solvent was concentrated *in vacuo* and the residue obtained was purified by column chromatography over silica gel (Petroleum ether/EtOAc 8:2, 7:3, 6:4, 5:5) to afford (−)-1D-**20a** (187 mg, 99%) as a colourless oil: TLC (Petroleum ether:EtOAc, 5:5 v/v): R_f = 0.44; $[\alpha]_D^{25}$ = −11.7 (*c* 0.74, $CHCl_3$); ^1H NMR (400 MHz; $CDCl_3$): δ 7.34–7.17 (m, 5H, H-13 to H-17), 5.84 (dddd, J = 17.2 Hz, 10.6, 5.4, 5.2 Hz, 1H, H-9), 5.26–5.18 (m, 1H, H-10), 5.14–5.08 (m, 1H, H-10’), 4.84 (d, J_{AB} = 11.7 Hz, 1H, H-11), 4.64 (d, J_{AB} = 11.7 Hz, 1H, H-11’), 4.40–4.33 (m, 1H, H-8), 4.13–4.04 (m, 1H, H-8’), 4.05–4.00 (m, 1H, H-2), 3.87 (dd, J = 9.7, 9.4 Hz, 1H, H-4), 3.59 (s, 3H, H-7), 3.51–3.44 (m, 1H, H-1), 3.41 (dd, J = 9.4, 8.9 Hz, 1H, H-5), 3.24 (dd, J = 8.9, 8.7 Hz, 1H, H-6), 3.16 (dd, J = 9.7, 1.2 Hz, 1H, H-3), 2.43 (d, J = 6.8 Hz, 1H, C(1)O*H*), 2.11–2.02 (m, 1H, H_{eq}-23), 1.82–1.73 (m, 1H, H_{ax}-20), 1.70–1.58 (m, 2H, H-22 and H_{eq}-21), 1.36 (d, J = 13.6 Hz, 1H, H_{ax}-23), 1.34–1.25 (m, 1H, H_{ax}-20), 1.18–1.08 (m, 1H, H_{ax}-21), 0.98 (s, 3H, C(24)C*H*$_3$), 0.792 (s, 3H, C(27)C*H*$_3$), 0.785 (s, 3H, C(24)C*H*$_3$); ^{13}C NMR (101 MHz; $CDCl_3$): δ 138.8 (C-12), 134.4 (C-9), 128.3 (C-14, C-16), 127.8 (C-13, C-17), 127.5 (C-15), 120.4 (C-18), 117.2 (C-10), 85.4 (C-6), 80.6 (C-5), 77.3 (C-4), 76.8 (C-3), 73.8 (C-2), 73.3 (C-1), 73.1 (C-8), 72.5 (C-11), 61.7 (C-7), 52.9 (C-19), 48.3 (C-24), 46.1 (C-23), 45.0 (C-22), 28.9 (C-20), 26.7 (C-21), 20.3, 20.2 (C-25, C-26), 9.7 (C-27); IR (thin film): 3426 (O-H) (br), 2933 (m), 1454 (w), 1312 (w), 1262 (w), 1202 (m), 1163 (m), 1088 (s), 1069 (s), 1032 (s), 949 (m), 926 (m), 777 (w), 740 (m), 698 (m) cm^{-1}; LRMS (m/z): $[M+H]^+$ 459.3 ($[M+H]^+$;

HRMS (m/z): $[M+Na]^+$ calcd. for $C_{27}H_{38}O_6Na$, 481.25606; found 481.25495; RP-HPLC (Method 2) t_R = 29.92 min, 97.42%.

Similarly, from (−)-1L-**21** (253 mg, 0.399 mmol, 1.0 eq) and ground NaOH (129 mg, 3.23 mmol, 8.1 eq) in MeOH (34 mL), (+)-1L-**20c** (190 mg, 89%) was obtained as a colourless oil: $[\alpha]_D^{25}$ = +11.9 (*c* 0.70, $CHCl_3$). All other data (R_f, ^1H NMR, ^{13}C NMR, IR, LRMS, HRMS, HPLC) matched those of the opposite enantiomer (−)-1D-**20a**.

Synthetic protocol and characterisation data for (−)-1D-2-*O*-allyl-5-*O*-benzyl-6-*O*-methyl-*myo*-inositol i.e. (−)-1D-(19) & (+)-1L-2-*O*-allyl-5-*O*-benzyl-6-*O*-methyl-*myo*-inositol i.e. (+)-1L-(19)

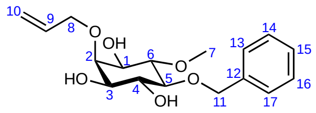

(−)-1D-(**19**)

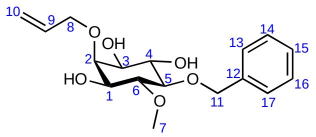

(+)-1L-(**19**)

Acetyl chloride (13 µL, 0.192 mmol, 0.60 eq) was added to a solution of (−)-1D-**20a** (147 mg, 0.321 mmol, 1.0 eq) in $MeOH/CH_2Cl_2$ (1.0:1.8 *v/v*, 2.2 mL). After stirring at RT for 16.5 hours, the reaction was quenched with Et_3N (27 µL, 0.192 mmol, 0.60 eq) and the solvents were removed *in vacuo*. The off-white solid obtained was then crystallised from EtOAc to give (−)-1D-**19** (99 mg, 95%, >95% *e.e.*) as a colourless crystalline solid: $[\alpha]_D^{25}$ = −15.4 (*c* 1.0, $CHCl_3$); RP-HPLC (Method 1) t_R = 9.05 min, 100%; Chiral HPLC (Heptane/IPA 90:10, 0.8 $mL.min^{-1}$, 254 nm) t_R = 18.88 min, 2.39% (+)-1L-**19**, t_R = 21.43 min, 97.61% (−)-1D-**19**, >95% *e.e.* All other data (R_f, mp, ^1H NMR, ^{13}C NMR, IR, LRMS, HRMS, Purity) match those of the racemic product (±)-**19**.

Similarly, by treating (+)-1L-**20c** (158 mg, 0.345 mmol, 1.0 eq) with acetyl chloride (15.0 µL, 0.207 mmol, 0.60 eq) in $MeOH/CH_2Cl_2$ (0.57:1.0 *v/v*, 2.4 mL), the opposite enantiomer (+)-1L-**19** (103 mg, 93%, >99% *e.e.*) was obtained as a colourless crystalline solid: $[\alpha]_D^{25}$ = +15.9 (*c* 1.0, $CHCl_3$); Chiral HPLC (Heptane/IPA 90:10, 0.8 $mL.min^{-1}$, 254 nm) t_R = 19.08 min, 99.99% (+)-1L-**19**, >99% *e.e.* (other enantiomer not observed, t_R = 21.43 min). All other data (R_f, mp, ^1H NMR, ^{13}C NMR, IR, HRMS, Purity) match those of the racemic product (±)-**19**.

The key intermediates (−)-1D-**19** and (+)-1L-**19** were studied using single crystal X-ray diffraction (See CIF file). Raw frame data were collected using an Oxford Diffraction SuperNovaA and the structure solved with SuperFlip[15] and refined with CRYSTALS[16]. In both cases, the Flack x, Hooft y parameter and Bayesian probabilities are all strongly indicative of the given enantiomer[17], however, both structures exhibited extremely large displacement ellipsoids. Several datasets were collected at low temperature and at room temperature with different thermal treatment, but in all cases, on close inspection of the raw diffraction images there was some evidence of diffuse scattering and weak super-lattice reflections believed to be due to modulation though the reflections were too weak to integrate. It is believed that this should have little or no influence on the absolute structure determination. X-ray crystallographic data for these compounds is included below.

Synthetic protocol and characterisation data for (−)-1D-2-*O*-allyl-1,3,4,5-tetra-*O*-benzyl-6-*O*-methyl-*myo*-inositol i.e. (−)-1D-(28) & (+)-1L-2-O-allyl-1,3,4,5-tetra-*O*-benzyl-6-*O*-methyl-*myo*-inositol i.e. (+)-1L-(28)

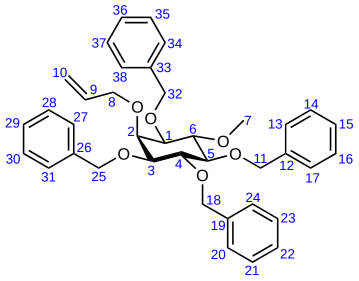

(−)-1D-(**28**)

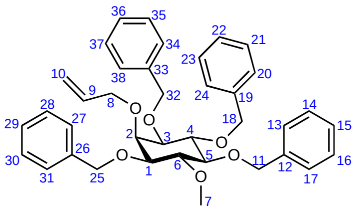

(+)-1L-(**28**)

To a solution of (−)-1D-**19** (116 mg, 0.358 mmol, 1.0 eq) in DMF (3.0 mL) at 0 °C was added NaH (86 mg, 2.15 mmol, 6.0 eq, 60% dispersion in mineral oil) portionwise. The reaction mixture was stirred at 0 °C for 10 minutes, warmed to RT and stirred for a further 30 minutes. After cooling once again to 0 °C, BnBr (251 μL, 2.11 mmol, 5.9 eq) was added dropwise. The reaction mixture was stirred for 10 minutes at 0 °C, then warmed to RT and stirred for 20 hours. The reaction was quenched by addition of H_2O (2.0 mL), then diluted in EtOAc (5 mL). The layers were separated and the aqueous phase was extracted with EtOAc (4 × 10 mL). The combined organic layers were washed with H_2O (2 × 10 mL), brine (2 × 10 mL), and an aqueous solution of LiCl (10% *w/v*, 2 × 10 mL), then dried ($MgSO_4$), filtered, and concentrated *in vacuo*. The

residue obtained was purified by column chromatography over silica gel (Petroleum ether/EtOAc 9.5:0.5, 9:1, 8.5:1.5, 8:2) to give (−)-1D-**28** (200 mg, 94%) as a colourless solid: mp: 70–72 °C; TLC (Petroleum ether:EtOAc, 1:1 v/v): R_f = 0.90; $[\alpha]_D^{25}$ = −13.4 (*c* 1.0, $CHCl_3$); ^1H NMR (400 MHz; $CDCl_3$): δ 7.42–7.29 (m, 20H, H-13 to H-17, H-20 to H-24, H-27 to H-31 and H-34 to H-38), 5.98 (dddd, J = 17.2, 10.2, 5.5, 5.6 Hz, 1H, H-9), 5.34–5.26 (m, 1H, H-10), 5.22–5.16 (m, 1H, H-10'), 4.90 (d, J_{AB} = 10.6 Hz, 1H, H-11), 4.86 (s, 2H, H-18), 4.82 (d, J_{AB} = 10.6 Hz, 1H, H-11'), 4.65–4.54 (m, 4H, H-25 and H-32), 4.34–4.30 (m, 2H, H-8), 3.99 (dd, J = 9.5, 9.5 Hz, 1H, H-4), 3.95 (dd, J = 2.2, 2.2 Hz, 1H, H-2), 3.74 (dd, J = 9.5, 9.5 Hz, 1H, H-6), 3.68 (s, 3H, H-7), 3.36 (dd, J = 9.5, 9.5 Hz, 1H, H-5), 3.31 (dd, J = 9.5, 2.2 Hz, 1H, H-3), 3.22 (dd, J = 9.5, 2.2 Hz, 1H, H-1); ^{13}C NMR (101 MHz; $CDCl_3$): δ 139.1 (C-12, C-19), 138.7, 138.5 (C-26, C-33), 136.0 (C-9), 128.53, 128.48, 128.4, 128.2, 128.1, 127.8, 127.73, 127.65, 127.6 (C-13 to C-17, C-20 to C-24, C-27 to C-31, C-34 to C-38), 116.8 (C-10), 84.0 (C-5), 83.7 (C-6), 81.7 (C-4), 80.9 (C-3), 80.8 (C-1), 75.99 (C-11), 75.97 (C-18), 74.2 (C-2), 73.5 (C-8), 72.9 (C-32, C-25), 61.5 (C-7); IR (thin film): 2956 (m), 2919 (s), 2851 (m), 2362 (m), 2338 (m), 1457 (w), 1361 (w), 1261 (w), 1159 (w), 1130 (m), 1090 (s), 1043 (m), 801 (w), 735 (m), 698 (m) cm^{-1}; LRMS (m/z): $[M+Na]^+$ 617.3; HRMS (m/z): $[M+Na]^+$ calcd. for $C_{38}H_{42}O_6Na$, 617.28736; found 617.28706; Chiral HPLC (Heptane/IPA 95:5, 1.0 $mL.min^{-1}$, 254 nm) t_R = 11.94 min, 99.85%.

Similarly, (+)-1L-**19** (116 mg, 0.357 mmol, 1.0 eq), NaH (86.0 mg, 2.15 mmol, 6.0 eq, 60% dispersion in mineral oil), and BnBr (251 µL, 2.11 mmol, 5.9 eq) in DMF (3 mL) afforded (+)-1L-**28** (191 mg, 90%) as a colourless solid: $[\alpha]_D^{25}$ = +12.3 (*c* 1.0, $CHCl_3$). All other data (R_f, mp, ^1H NMR, ^{13}C NMR, IR, LRMS, HRMS, Purity) match those of the opposite enantiomer (−)-1D-**28**.

Synthetic protocol and characterisation data for (−)-1D-1,3,4,5-Tetra-*O*-benzyl-6-*O*-methyl-*myo*-inositol i.e. (−)-1D-(22) & (+)-1L-1,3,4,5-tetra-*O*-benzyl-6-*O*-methyl-*myo*-inositol i.e. (+)-1L-(22)

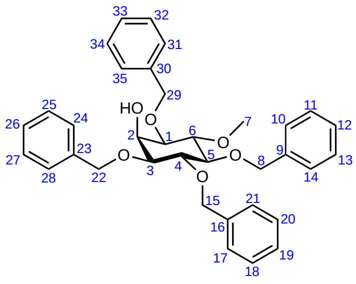

(−)-1D-(**22**)

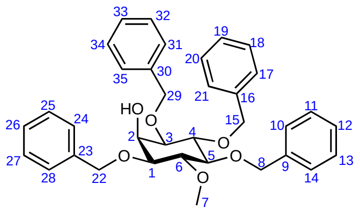

(+)-1L-(**22**)

To a solution of (−)-1D-**28** (182 mg, 0.306 mmol, 1.0 eq) in MeOH/CH_2Cl_2 (2.5:1.0 *v*/*v*, 2.2 mL) was added $PdCl_2$ (6.0 mg, 0.0337 mmol, 0.11 eq). The reaction mixture was stirred at RT for 19 hours, then diluted in CH_2Cl_2 (5 mL). The solution was filtered through a short pad of Celite®, washed with CH_2Cl_2 (5 × 10 mL), and the filtrate was concentrated *in vacuo*. Purification by column chromatography over silica gel (Petroleum ether/EtOAc 8:2, 7:3) afforded (−)-1D-**22** (160 mg, 94%) as a colourless oil: TLC (Petroleum ether:EtOAc, 7:3 v/v): R_f = 0.32; $[\alpha]_D^{25}$ = −19.4 (*c* 0.81, $CHCl_3$); ^{1}H NMR (400 MHz; $CDCl_3$): δ 7.41–7.26 (m, 20H, H-10 to H-14, H-17 to H-21, H-24 to H-28 and H-31 to H-35), 4.91–4.81 (m, 4H, H-8 and H-15), 4.77–4.66 (m, 4H, H-22 and H-29), 4.19 (dd, J = 2.7, 2.7 Hz, 1H, H-2), 3.95 (dd, J = 9.6, 9.6 Hz, 1H, H-5), 3.70 (dd, J = 9.6, 9.6 Hz, 1H, H-6), 3.68 (s, 3H, H-7), 3.39–3.32 (m, 2H, H-3 and H-4), 3.28 (dd, J = 9.6, 2.7 Hz, 1H, H-1), 2.43 (s, 1H, C(2)O*H*); ^{13}C NMR (101 MHz; $CDCl_3$): δ 139.0, 138.9 (C-9, C-16), 138.3, 138.1 (C-23, C-30), 128.6, 128.49, 128.48, 128.12, 128.11, 128.01, 127.98, 127.96, 127.92, 127.72, 127.69 (C-10 to C-14, C-17 to C-21, C-24 to C-28 and C-31 to C-35), 83.4 (C-4), 83.2 (C-6), 81.2 (C-5), 79.9 (C-3), 79.8 (C-1), 76.1, 76.0 (C-8, C-15), 72.92, 72.85 (C-22, C-29), 67.9 (C-2), 61.5 (C-7); IR (thin film): 3465 (O-H) (w), 3063 (w), 3030 (w), 2924 (w), 2878 (w),1497 (w), 1454 (w), 1360 (w), 1128 (m), 1085 (s), 1069 (s), 1028 (m), 732 (s), 696 (s) cm^{-1}; LRMS (m/z): $[M+Na]^+$ 577.3; HRMS (m/z): $[M+Na]^+$ calcd. for $C_{35}H_{38}O_6Na$, 577.25606; found 577.25541; RP-HPLC (Method 1) t_R = 15.95 min, 98.57%.

Similarly, from (+)-1L-**28** (173 mg, 0.291 mmol, 1.0 eq) and $PdCl_2$ (6 mg, 0.0320 mmol, 0.11 eq) in MeOH/CH_2Cl_2 (2.5:1.0 *v*/*v*, 2.0 mL), (+)-1L-**22** (150 mg, 93%) was obtained as a colourless oil: $[\alpha]_D^{25}$ = +19.6

(c 1.0, $CHCl_3$). All other data (R_f, mp, ^1H NMR, ^{13}C NMR, IR, LRMS, HRMS, Purity) match those of the opposite enantiomer (−)-1D-**22**.

Synthetic protocol and characterisation data for (+)-1D-2,4,5,6-tetra-*O*-benzyl-3-*O*-methyl-1-azido-1-deoxy-*scyllo*-inositol i.e. (−)-1D-(29) & (−)-1L-2,4,5,6-tetra-*O*-benzyl-3-*O*-methyl-1-azido-1-deoxy-*scyllo*-inositol i.e. (−)-1L-(29)

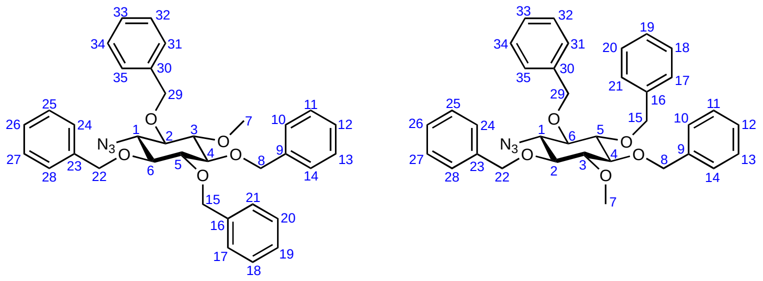

(+)-1D-**(29)**   (−)-1L-**(29)**

A solution of (−)-1D-**22** (250 mg, 0.451 mmol, 1.0 eq) in pyridine (4.0 mL) was cooled to 0 °C. Methanesulfonyl chloride (837 µL, 10.8 mmol, 24 eq) was added dropwise and the reaction mixture was gradually warmed to RT. After stirring for 20 hours, the reaction was quenched with H_2O (3 mL) and diluted with CH_2Cl_2 (3 mL). The aqueous layer was extracted with CH_2Cl_2 (3 × 5 mL) and the combined organic layers were washed with H_2O (2 × 3 mL) and brine (2 × 3 mL), then dried (Na_2SO_4), filtered, and concentrated *in vacuo*. Without any further purification, the yellowish oil obtained was dissolved in DMF (4.0 mL). NaN_3 (214 mg, 3.29 mmol, 7.3 eq) was added and the reaction mixture was heated at 90 °C for 18 hours. H_2O (3 mL) was added and the aqueous phase was extracted with EtOAc (2 × 5 mL). The combined organic layers were washed with H_2O (5 mL) and brine (5 mL), then dried ($MgSO_4$), filtered, and concentrated *in vacuo*. The yellow oil obtained was purified by column chromatography over silica gel (Hexane/EtOAc 10:2) to give (+)-1D-**29** (248 mg, 95%) as a gummy solid: TLC (Petroleum ether:EtOAc, 7:3 v/v): R_f = 0.77; $[\alpha]_D^{25}$ = +11.6 (c 0.82, $CHCl_3$); ^1H NMR (500 MHz; $CDCl_3$): δ 7.47–7.27 (m, 20H, H-10 to H-14, H-17 to H-21, H-24 to H-28 and H-31 to H-35), 4.90–4.81 (m, 8H, H-8, H-15, H-22 and H-29), 3.68 (s, 3H, H-7), 3.55–3.47 (m, 1H, H-4), 3.47–3.38 (m, 2H, H-1 and H-5), 3.32 (dd, J = 9.7, 9.7 Hz, 1H, H-2 or H-6), 3.28–

3.22 (m, 2H, H-3 and H-2 or H-6); ^{13}C NMR (126 MHz; $CDCl_3$): δ 138.5, 138.05, 137.97 (C-9, C-16, C-23, C-30), 128.63, 128.60, 128.57, 128.5, 128.4, 128.2, 128.11, 128.06, 127.9, 127.8 (C-10 to C-14, C-17 to C-21, C-24 to C-28 and C-31 to C-35), 85.5 (C-3), 83.2 (C-4), 82.8 (C-5), 81.3 (C-2 or C-6), 81.2 (C-2 or C-6), 76.11, 76.08, 76.03, 76.02 (C-8, C-15, C-22, C-29), 66.9 (C-1), 61.7 (C-7); IR (thin film): 3031 (w), 2919 (br), 2105 (N_3) (m), 1497 (w), 1454 (w), 1359 (w), 1264 (w), 1137 (m), 1063 (s), 1027 (m), 735 (m), 697 (s) cm^{-1}; LRMS (m/z): $[M+K]^+$ 618.3; HRMS (m/z): $[M+Na]^+$ calcd. for $C_{35}H_{37}O_5N_3Na$, 602.26254; found 602.26233. The data are in good agreement with the literature values for racemic (±)-**29**[13,14].

Similarly, the reaction of (+)-1L-**22** (300 mg, 0.541 mmol, 1.0 eq) and methanesulfonyl chloride (1.00 mL, 13.0 mmol, 24 eq) in pyridine (4.8 mL), followed by aqueous work-up, gave a residue which was dissolved in anhydrous DMF (4.8 mL). NaN_3 (257 mg, 3.95 mmol, 7.3 eq) was added and the reaction mixture was heated at 90 °C for 18 hours. Aqueous work-up and purification by column chromatography over silica gel (Hexane/EtOAc 10:2) yielded (−)-1L-**29** (280 mg, 89%) as a gummy solid: $[\alpha]_D^{25} = -12.5$ (*c* 1.0 in $CHCl_3$). All other data (R_f, ^{1}H NMR, ^{13}C NMR, IR, LRMS, HRMS) match those of the opposite enantiomer (+)-1D-**29** and those of the literature values for racemic racemic (±)-**29**[13,14].

Synthetic protocol and characterisation data for (−)-1D-2,4,5,6-tetra-*O*-benzyl-3-*O*-methyl-*scyllo*-inosamine i.e. (−)-1D-(30) & (+)-1L-2,4,5,6-tetra-*O*-benzyl-3-*O*-methyl-*scyllo*-inosamine i.e. (+)-1L-(30)

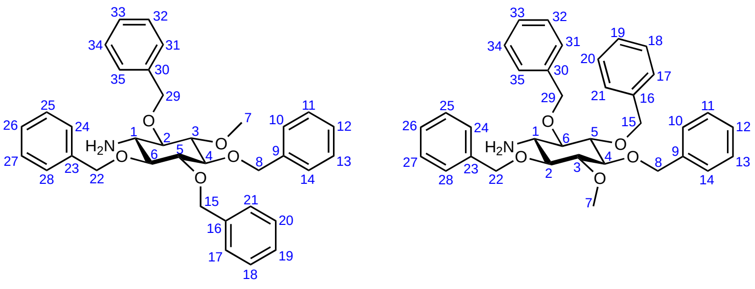

(−)-1D-**(30)** (+)-1L-**(30)**

To a solution of the azide (+)-1D-**29** (232 mg, 0.400 mmol, 1.0 eq) in THF (6.3 mL) were added PPh_3 (210 mg, 0.801 mmol, 2.0 eq) and H_2O (272 µL). The solution was stirred at 40 °C for 28 hours then cooled to RT. The volatile components were concentrated *in vacuo*. EtOAc (10 mL) and H_2O (5 mL) were added to the residue obtained. The aqueous layer was extracted with EtOAc (3 × 8 mL) and the combined organic layers

were washed with H_2O (8 mL), and brine (8 mL), then dried (Na_2SO_4), filtered, and concentrated *in vacuo*. Purification by column chromatography over silica gel (Petroleum ether/EtOAc/Et_3N 9:0.95:0.05, 8:1.95:0.05, 7:2.95:0.05, 6:3.95:0.05) yielded (−)-1D-**30** (134 mg, 60%) as a colourless, glassy solid: mp: 85–88 °C; TLC (Petroleum ether:EtOAc, 1:1 v/v): R_f = 0.36; $[\alpha]_D^{25}$ = −5.2 (*c* 2.0, $CHCl_3$); ^{1}H NMR (500 MHz; $CDCl_3$): δ 7.51–7.05 (m, 20H, H-10 to H-14, H-17 to H-21, H-24 to H-28 and H-31 to H-35), 4.943 (d, J_{AB} = 10.9 Hz, 1H, H-22 or H-29), 4.940 (d, J_{AB} = 10.9 Hz, 1H, H-22 or H-29), 4.88 (d, J_{AB} = 10.8 Hz, 1H, H-15), 4.85 (d, J_{AB} = 10.6 Hz, 1H, H-8), 4.82 (d, J_{AB} = 10.6 Hz, 1H, H-8'), 4.79 (d, J_{AB} = 10.8 Hz, 1H, H-15'), 4.66 (d, J = 10.9 Hz, 1H, H-22' or H-29'), 4.65 (d, J = 10.9 Hz, 1H, H-22' or H-29'), 3.65 (s, 3H, H-7), 3.53 (dd, J = 9.5, 9.1 Hz, 1H, H-5), 3.47 (dd, J = 9.1, 8.9 Hz, 1H, H-4), 3.30 (dd, J = 9.7, 9.5 Hz, 1H, H-6), 3.27 (dd, J = 9.1, 8.9 Hz, 1H, H-3), 3.23 (dd, J = 9.5, 9.1 Hz, 1H, H-2), 2.85 (dd, J = 9.7, 9.5 Hz, 1H, H-1), 1.81 (br s, 3H, C(1)NH_2); ^{13}C NMR (126 MHz; $CDCl_3$): δ 138.6, 138.55, 138.45 (C-9, C-16, C-23, C-30), 128.71, 128.68, 128.6 (C-11, C-13, C-18, C-20, C-25, C-27, C-32, C-34), 128.2, 128.1, 128.04, 128.00, 127.95, 127.86, 127.8 (C-10, C-12, C-14, C-17, C-19, C-21, C-24, C-26, C-28, C-31, C-33, C-35), 86.5 (C-3), 84.2 (C-5), 83.7 (C-4), 83.00, 82.98 (C-2, C-6), 76.1 (C-8), 75.94 (C-15), 75.86, 75.85 (C-22, C-29), 61.4 (C-7), 55.3 (C-1); IR (thin film): 3378 (N-H) (w), 3064 (w), 3030 (w), 2903 (w), 2360 (w), 2341 (w), 1581 (N-H) (w), 1497 (m), 1453 (m), 1353 (m), 1213 (w), 1150 (m), 1133 (m), 1065 (s), 1047 (s), 1027 (s), 940 (w), 803 (s), 753 (m), 735 (s), 695 (s), 661 (m), 626 (m) cm^{-1}; LRMS (m/z): $[M+H]^+$ 554.3; HRMS (m/z): $[M+H]^+$ calcd. for $C_{35}H_{40}O_5N$, 554.29010; found 554.28939; RP-HPLC (Method 1) t_R = 13.14 min, 95.81%.

Similarly, from the azide (−)-1L-**29** (261 mg, 0.450 mmol, 1.0 eq), PPh_3 (236 mg, 0.900 mmol, 2.0 eq) and H_2O (306 µL) in THF (7.1 mL), (+)-1L-**30** (136 mg, 63%) was obtained as a colourless, glassy solid: $[\alpha]_D^{25}$ = +4.7 (*c* 1.7, $CHCl_3$). All other data (R_f, ^{1}H NMR, ^{13}C NMR, IR, LRMS, HRMS) match those of the opposite enantiomer (−)-1D-**30**.

Synthetic protocol and characterisation data for (+)-1D-3-*O*-methyl-*scyllo*-inosamine hydrochloride i.e. (+)-1D-(2) & (−)-1L-3-*O*-methyl-*scyllo*-inosamine hydrochloride i.e. (−)-1L-(2)

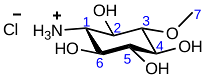

(+)-1D-**(2)**

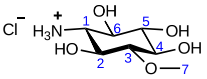

(−)-1L-**(2)**

To a solution of the protected amine (−)-1D-**30** (136 mg, 0.246 mmol, 1.0 eq) in CH_2Cl_2/MeOH/H_2O (3:2:1 *v/v/v*, 3.6 mL) was added 10% Pd/C (42 mg, 0.0393 mmol, 0.16 eq) and molecular biology grade concentrated HCl (23 µL) under $N_{2(g)}$. The flask was purged with 3 balloons of $H_{2(g)}$, and the suspension was stirred at RT, under $H_{2(g)}$ for 20 hours. The reaction mixture was then filtered through a glass microfiber filter and washed with H_2O (5 × 5 mL). The filtrate was lyophilised to give a yellowish amorphous solid. The colourless solid obtained was dissolved in the minimal amount of H_2O (0.5 mL) and loaded onto a pre-washed DOWEX® 50WX8 cation ion exchange column (~0.9 mL of resin). The column was eluted with H_2O (10 mL), then with a 0.10 M aqueous solution of HCl (10 mL). The fractions were combined and lyophilised to give the hydrochloride salt (+)-1D-**2** (43 mg, 72%) as a colourless solid: decomp. 290–291 °C; $[\alpha]_D^{25}$ = +2.6 (*c* 2.0 in $CHCl_3$); ^1H NMR (500 MHz; D_2O): δ 3.67–3.59 (m, 4H, H-2 and H-7), 3.55–3.49 (m, 1H, H-4), 3.47–3.40 (m, 2H, H-5 and H-6), 3.22 (dd, J = 8.4, 8.4 Hz, 1H, H-3), 3.13 (dd, J = 10.5, 10.5 Hz, 1H, H-1); ^{13}C NMR (126 MHz; D_2O): δ 84.0 (C-3), 74.1 (C-5), 72.5 (C-6), 69.7 (C-4), 69.3 (C-2), 60.3 (C-7), 55.6 (C-1); LRMS (m/z): $[M+H]^+$ 194.2; HRMS (m/z): $[M+H]^+$ calcd. for $C_7H_{16}O_5N$, 194.10230; found 194.10254; GC-MS (derivatised with TMSI) t_R = 15.97 min, 97.27%. The data are in good agreement with the literature values for (±)-**2**[13,14].

Similarly, reaction of (+)-1L-**30** (158 mg, 0.285 mmol, 1.0 eq) and 10% Pd/C (49 mg, 0.0457 mmol, 0.16 eq) in CH_2Cl_2/MeOH/H_2O (3:2:1 *v/v/v*, 4.3 mL) and under $H_{2(g)}$, yielded (−)-1L-**2** (53.6 mg, 77%) as a colourless solid: $[\alpha]_D^{25}$ = −2.2 (*c* 2.0, $CHCl_3$). All other data (mp, ^1H NMR, ^{13}C NMR, LRMS, HRMS) match those of the opposite enantiomer (+)-1D-**2**.

Synthetic protocol and characterisation data for (±)-2-keto-1,3,4,5-tetra-*O*-benzyl-6-*O*-methyl-*myo*-inositol i.e. (±)-(23)

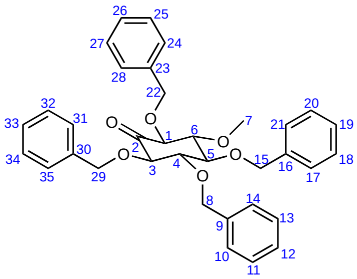

(±)-**(23)**

A solution of anhydrous DMSO (196 µL, 2.76 mmol, 2.2 eq) in anhydrous CH_2Cl_2 (0.40 mL) was added dropwise over 5 minutes to a solution of oxalyl chloride (117 µL, 1.38 mmol, 1.1 eq) in CH_2Cl_2 (2.0 mL) at −60 °C. A solution of (±)-**22** (695 mg, 1.25 mmol, 1.0 eq) in anhydrous CH_2Cl_2 (1.6 mL) was then added dropwise to the reaction mixture and the solution was stirred for a further 2.5 hours, maintaining the temperature between −60 °C and −55 °C. Anhydrous Et_3N (803 µL, 5.76 mmol, 4.6 eq) was then added and the reaction was stirred for a further 5 minutes before warming up to RT. After 1 hour, H_2O (3 mL) was added and the reaction mixture was diluted in CH_2Cl_2 (6 mL). The organic phase was separated and the aqueous layer was extracted with CH_2Cl_2 (3 × 6 mL). The combined organic layers were washed with brine (6 mL) and H_2O (6 mL), then dried ($MgSO_4$), filtered, and concentrated *in vacuo*. Purification by column chromatography over silica gel (Petroleum ether/EtOAc 9.5:0.5, 9:1, 8:2, 7:3) followed by crystallisation from Et_2O afforded (±)-**23** (320 mg, 46%) as a colourless solid: m.p. 97–99 °C; TLC (Petroleum ether:EtOAc, 7:3, v/v): R_f = 0.86; ^{1}H NMR (500 MHz; $CDCl_3$): δ 7.43–7.27 (m, 20H, H-10 to H-14, H-17 to H-21, H-24 to H-28 and H-31 to H-35), 4.91–4.83 (m, 5H, H-8, H-15, H-22 and H-29), 4.76 (d, J_{AB} = 10.6 Hz, 1H, H-8'), 4.58 (d, J_{AB} = 11.7 Hz, 1H, H-29'), 4.53 (d, J_{AB} = 11.5 Hz, 1H, H-22'), 4.12 (dd, J = 9.8, 1.5 Hz, 1H, H-3), 4.04 (dd, J = 9.9, 1.5 Hz, 1H, H-1), 3.77 (dd, J = 9.4, 9.3 Hz, 1H, H-5), 3.65 (s, 3H, H-7), 3.57 (dd, J = 9.8, 9.4 Hz, 1H, H-4), 3.32 (dd, J = 9.9, 9.3 Hz, 1H, H-6); ^{13}C NMR (126 MHz; $CDCl_3$): δ 202.2 (C-2), 138.4, 138.3 (C-9, C-16), 137.6 (C-30), 137.5 (C-23), 128.6, 128.55, 128.52, 128.3, 128.21, 128.19, 128.11, 128.08, 127.93, 127.91 (C-10 to C-14, C-17 to C-21, C-24 to C-28, C-31 to C-35), 83.8 (C-3), 83.7 (C-1), 83.5 (C-6), 82.4 (C-5), 81.5 (C-4), 76.25 (C-15), 76.16 (C-8), 73.5 (C-29), 73.4 (C-22), 61.8 (C-7); IR (thin film): 3063 (w), 3031 (w), 2909 (w), 2867 (w), 1731 (C=O) (m), 1497 (w), 1454 (w), 1360 (w), 1214 (w), 1133 (m), 1065 (s), 1026 (s), 970 (m),

921 (w), 754 (m), 732 (m), 695 (s) cm^{-1}; LRMS (m/z): $[M+Na]^+$ 575.2; HRMS (m/z): $[M+Na]^+$ calcd. for $C_{35}H_{36}O_6Na$, 575.24041; found 575.24020; RP-HPLC (Method 2) 16.01 min, 99.80%.

Synthetic protocol and characterisation data for (±)-3-*O*-Methyl-*scyllo*-inosose i.e. (±)-6

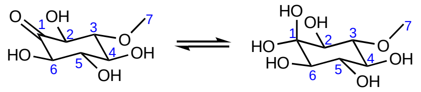

(±)-6 **(±)-31**

To a solution of (±)-**23** (27 mg, 0.0494 mmol, 1.0 eq) in MeOH/CH_2Cl_2/H_2O/AcOH (5:2:1:1 *v/v/v/v* 3.6 mL) was added 10% Pd/C (7 mg, 0.00658 mmol, 0.13 eq) under $N_{2(g)}$. The flask was purged with 3 balloons of $H_{2(g)}$, and the suspension was stirred at RT, under $H_{2(g)}$ for 17 hours. The reaction mixture was then filtered through a microfibre glass filter and washed with H_2O (3 × 2 mL). The filtrate was lyophilised to give (±)-**6** (8 mg, 84%) as a colourless solid. This compound was found to be very unstable, and in equilibrium with the hydrate form (±)-**31** as observed by ^{1}H NMR ((±)-**6** (Ket.)/(±)-**31** (Diol) 1.3:1.0). As a result, clean ^{1}H NMR and ^{13}C NMR data for this compound could not be obtained. This compounds was made fresh before use in *in vitro* assays: mp: 95–97 °C; TLC (EtOAc:H_2O:IPA, 1:1:1 v/v/v): $R_f = 0.55$; ^{1}H NMR (500 MHz; D_2O): δ 4.51 (dd, J = 10.2, 1.6 Hz, 1H, H-2 Ket.), 4.37 (dd, J = 10.3, 1.6 Hz, 1H, H-6 Ket.), 3.89 (dd, J = 9.7, 9.5 Hz, 1H, H-4 Ket.), 3.62 (s, 3H, H-7 Ket.), 3.59 (s, 3H, H-7 Diol), 3.50–3.34 (m, 5H, H-5 Ket., H-6, H-5, H-4 and H-2 Diol), 3.23 (dd, J = 10.2, 9.7 Hz, 1H, H-3 Ket.), 3.24–3.16 (m, 1H, H-3 Diol); ^{13}C NMR (126 MHz; D_2O): δ 205.6 (C-1 Ket.), 94.1 (C-1 Diol), 83.3 (C-3 Ket.), 83.0 (C-3 Diol), 75.9 (C-6 Ket.), 75.5 (C-2 Ket.), 74.1 (C-6 Diol), 73.7 (C-2 Diol), 73.6 (C-5 Ket.), 73.14, 73.07 (C-5, C-4 Diol), 72.3 (C-4 Ket.), 59.8 (C-7 Diol), 59.7 (C-7 Ket.); IR (thin film): 3089 (w), 3063 (w), 3030 (w), 2911 (w), 2867 (w), 1731 (C=O) (m), 1497 (w), 1454 (m), 1361 (m), 1215 (w), 1133 (s), 1065 (s), 1035 (s), 1026 (s), 970 (m), 921 (m), 754 (s), 734 (s), 695 (s) cm^{-1}; LRMS (m/z): $[2M+Na]^+$ 407.2 (Ket.); $[2M+H]^+$ 421.0 (Diol); HRMS (Ket.) *m/z* (ESI^-) found 227.03279 $[M+Cl]^-$ ($C_7H_{12}O_6Cl$ requires 227.03279 $[M+Cl]^-$); HRMS (m/z): $[M+Cl]^-$ calcd. for $C_7H_{14}O_7Cl$, 245.04335; found 245.04314. The data are in good agreement with the literature values.[18]

Supplementary Table 1. Bacterial strains

**Organism**	**Description/Genotype**	**Source**
Agrobacterium tumefaciens		
GV3101:pMP90	C58 pMP90(pTiC58DT-DNA), Gm^R, Rf^R	[19]
AGL1	C58 *recA* pTiBo542DT-DNA, Rf^R, $Carb^R$	[20]
Agrobacterium rhizogenes		
AR1193	C58 pRi1193 carrying pBR322 Rf^R, $Carb^R$	[21]
Escherichia coli		
DH5-α	F^- *endA1 glnV44 thi-1 recA1 relA1 gyrA96 deoR nupG purB20* φ80d*lacZ*ΔM15 Δ(*lacZYA-argF*)U169, hsdR17($r_K^- m_K^+$), $λ^-$	Bioline
BL21 (DE3)	F^- *ompT gal dcm lon* $hsdS_B(r_B^- m_B^-)$ λ(DE3 [*lacI lacUV5-T7p07 ind1 sam7 nin5*]) [*malB*$^+$]$_{K\text{-}12}$($λ^S$)	Bioline
Rhizobium leguminosarum		
3841	Wild-type, St^R	[22]
UPM1137	Wild-type	[23]
OPS1341	3841 with mCherry3 expression cassette integrated into genome by Tn7, St^R, Gm^R	Jorrin Rubio *et al.* in preparation.
Sinorhizobium meliloti		
BL225C	Wild-type	[24]
CO431A	Wild-type	[25]
L5-30	Wild-type, St^R	[26]
OPS0646	L5-30 *mosB*::pK19, St^R, Nm^R	This work
OPS0648	L5-30 *mosE*::pK19, St^R, Nm^R	This work
Rm1021	Wild-type, St^R	[27]
SM11	Wild-type	[28]

Supplementary Table 2. Bacterial plasmids

**Name**	**Description/Genotype**	**Reference**
pMQ131	Yeast gap repair cloning compatible plasmid, Km^R	[29]
pOGG024	Level 1 golden gate cloning destination vector for free-living expression, Gm^R<br>pL1V-gent-pBBR1-ELT3, Gm^R	[30]
pOPINF	Cloning vector for T7RNAP based protein purification in *E. coli*, Amp^R	[31]
pIJ11268	Cloning vector for construction of *lux* reporters that are stable in the environment, Tc^R	[32]
pOPS0046	pIJ11268 *mocRBluxCDABE* rhizopine *lux* reporter, Tc^R	This work
pOPS0241	pOPINF *iolG*, for IolG purification from BL21, Amp^R	This work
pOPS0243	pK19 *mosB*, for integration into L5-30 *mosB*, Km^R	This work
pOPS0244	pK19 *mosE* for integration into L5-30 *mosE*, Km^R	This work
pOPS0362	pMQ131par *mosDEF*, for stable nodule expression of rhizopine synthesis genes, Km^R	This work
pOPS0363	pOGG024 *MBP-mosB,* for MosB purification from *R. leguminosarum*, Gm^R	This work
pOPS0761	pMQ131par *mocRBGFPmut3* rhizopine *GFP* reporter, Km^R	This work
pRK2013	Self transmissible helper plasmid, Km^R	[33]
pTNS3	Plasmid expressing *tnsABCD* from *P1* and *Ptac*	[34]
pUC18T-miniTn*7*T-Gm	For Tn*7* insertion into Gm^S bacteria, Amp^R, Gm^R	[35,36]

Supplementary Table 3. Plant transformation plasmids

Plasmid	Description	Reference
pEC50505	Level 2 cloning vector for plant engineering, Km^R	[37]
pEC11281	EC50505-eGFP, for plant transient transformation, Km^R	This work
pEC12824	EC50505-eGFP-mosA-mosB-mosC, for plant transient transformation, Km^R	This work
pEC12805	EC50505-eGFP-IMT-mosDEF-mosB, for plant transient transformation, Km^R	This work
pEC11910	EC50505-eGFP-IDH, for plant transient transformation, Km^R	This work
pEC11906	EC50505-eGFP-mosB, for plant transient transformation, Km^R	This work
pEC11912	EC50505-eGFP-IDH-mosB, for plant transient transformation, Km^R	This work
pEC12825	EC50505-HYG, for barley stable transformation, Km^R	This work
pEC12811	EC50505-HYG-IDH-mosB, for barley stable transformation, Km^R	This work

Supplementary Table 4. Primers

Primer	Sequence (5'→ 3')	Description
oxp0024	AGGAGGAAGAACATATGATGTTTGAGGGTT CGATTAC	Forward primer for amplification of *mosABC* ORFs for cloning into pLMB509 by Infusion BD cloning
oxp0053	TGGTGATGATGCATATGTCAAGGCTCTGGC TGGCC	Reverse primer for amplification of *mosABC* ORFs for cloning into pLMB509 by Infusion BD cloning
oxp0069	ATATGGATCCTCAGTTCTTTCCCTGAAACTC G	Foreword primer for amplification of *mocRB* ORFs for cloning into pIJ11268 BamH1 site
oxp0070	ATATGGATCCTCAGCAACCACGTGGAGCAG	Reverse primer for amplification of *mocRB* ORFs for cloning into pIJ11268 BamH1 site
oxp0558	AAGTTCTGTTTCAGGGCCCGATGAGTTTAC GTATTGGC	Foreword primer for amplification of *iolG* ORF for cloning into pOPINF by Infusion BD cloning
oxp0559	CTGGTCTAGAAAGCTTTAGTTTTGAACTGTT GTAAAAG	Reverse primer for amplification of *iolG* ORF for cloning into pOPINF by Infusion BD cloning
oxp0560	TGATTACGCCAAGCTTTGTGGTCTCGGCAA GTAGC	Foreword primer for amplification of *mosB* internal fragment for cloning into pK19 by Infusion BD cloning
oxp0561	GCAGGCATGCAAGCTTCGCGGAAAACGGCA TAACC	Reverse primer for amplification of *mosB* internal fragment for cloning into pK19 by Infusion BD cloning
oxp0564	TGATTACGCCAAGCTTAGGGGGAAGGTGAC TAACGA	Foreword primer for amplification of *mosE* internal fragment for cloning into pK19 by Infusion BD cloning
oxp0565	GCAGGCATGCAAGCTTCTTGAGAGCGGGTC ATACGG	Reverse primer for amplification of *mosE* internal fragment for cloning into pK19 by Infusion BD cloning
oxp0555	TTAGAGTCACTAAGGGCTAACTAACTAATT ACGTAGAATTCACGTCGCTTCAG	Foreword primer for amplification of PmosB for yeast recombineering with *mosDEF* in pMQ131par.
oxp0576	GTGGGCGCCAAGGGTTCGCGCATGGGATGA GGCATATTACCCCCATGTC	Reverse primer for amplification of PmosB for yeast recombineering with *mosDEF* in pMQ131par.
oxp0552	TCATCCCATGCGCGAAC	Foreword primer for amplification of *mosDEF* for yeast recombineering with PmosB in pMQ131par.
oxp0578	CGTAGGGCGCATTAATGCAGCTGGCACGAC AGGTGAATTCCGAAATTGCCTAAGGTGCC	Reverse primer for amplification of *mosDEF* for yeast recombineering with PmosB in p pMQ131par
oxp1436	CCCCTCAAGACCCGTTTAGAGGCCCCAATC TAGATCTTTGGGACATGCGATCGTATTGCCT ATG	Reverse primer for amplification of *GFPmut3.1* for yeast recombineering with *mocRB* in pMQ131par
oxp1800	TAGCCCTTAGTGACTCTAATACGACTCACTA TTGGGAGATTCAGCAACCACGTGGAGC	Forward primer for amplification of *mocRB* for yeast recombineering with *GFPmut3.1* in pMQ131par
oxp1801	AAAAACGGGTATGGAGAAGGATCCTCAGTT CTTTCCCTGAAACTCGG	Reverse primer for amplification of *mocRB* for yeast recombineering with *GFPmut3.1* in pMQ131par
oxp1802	AGTTTCAGGGAAAGAACTGAGGATCCTTCT CCATACCCGTTTTTTTGGGCT	Forward primer for amplification of *GFPmut3.1* for yeast recombineering with *mocRB* in pMQ131par
GFP F	TGTTTGAACGATCTGCTTGACA	Forward primer for verification of GFP CDS in recombinant plasmid by PCR

GFP R	CAAGCTGACCCTGAAGTTCATCT	Reverse primer for verification of GFP CDS in recombinant plasmid by PCR
mosA F	ATCCTCTCAGCGAACCTAGC	Forward primer for verification of *mosA* CDS in recombinant plasmid by PCR
mosA R	TGCCCTAACGTTAAGGGTGT	Reverse primer for verification of *mosA* CDS in recombinant plasmid by PCR
mosB F	CACGATGAAAGAGTTCTCGATAGC	Forward primer for verification of *mosB* CDS in recombinant plasmid by PCR
mosB R	CATCTCTCCTACCGCTCTCG	Reverse primer for verification of *mosB* CDS in recombinant plasmid by PCR
mosC F	AGCCATTCCAAGTCCAAGGA	Forward primer for verification of *mosC* CDS in recombinant plasmid by PCR
mosC R	ATCTACCGGAATCCTCGCTG	Reverse primer for verification of *mosC* CDS in recombinant plasmid by PCR
IDH F	CCGTAGGTAGCCCTTCTAGAGTTAG	Forward primer for verification of *idhA* CDS in recombinant plasmid by PCR
IDH R	GATCGGAAAGGTTCACGCTAA	Reverse primer for verification of *idhA* CDS in recombinant plasmid by PCR
IMT F	CTCGCATATCTCATTAAAGCAGG	Forward primer for verification of *imt* CDS in recombinant plasmid by PCR
IMT R	CAGATCGGAGCTAAGAACCCTA	Reverse primer for verification of *imt* CDS in recombinant plasmid by PCR
mosD F	TCATATCTTGCACCAGGGAATC	Forward primer for verification of *mosD* CDS in recombinant plasmid by PCR
mosD R	CGTCGTGAATTGCTTTTCCG	Reverse primer for verification of *mosD* CDS in recombinant plasmid by PCR
mosE F	GCTCCGATGAATGGGTTGAT	Forward primer for verification of *mosE* CDS in recombinant plasmid by PCR
mosE R	TGGAATCGCTGGAAAGGCTG	Reverse primer for verification of *mosE* CDS in recombinant plasmid by PCR
mosF F	CTTCTGGTTGAATGGAGGCAT	Forward primer for verification of *mosF* CDS in recombinant plasmid by PCR
mosF R	GGAGTGGCTGTGATCGCTAC	Reverse primer for verification of *mosF* CDS in recombinant plasmid by PCR
HYG F	TGCATCATCGAAATTGCCGT	Forward primer for verification of *hyg* CDS in recombinant plasmid by PCR
HYG R	CGATTGCTGATCCCCATGTG	Reverse primer for verification of *hyg* CDS in recombinant plasmid by PCR

Supplementary Table 5. Determination of the absolute configuration of (−)-1D-(20a) using α-methoxyphenylacetic acid as a chiral anisotropy reagent.

Position	δ^R (−)-**25**	δ^S (+)-**25**[b]	$\Delta\delta^{RSa}$	**L_1 or L_2**
1	4.75	4.83	−0.08	N/A
2	4.18	3.95	0.23	L_2
3	3.26	3.22	0.04	L_2
4	3.94	3.93	0.01	N/A
5	3.45	3.51	−0.06	L_1
6	3.46	3.59	−0.13	L_1
7	3.06	3.52	−0.46	L_1
8[c]	4.23	3.89	0.34	L_2
8’[c]	4.00	3.59	0.41	L_2
9	5.80	5.46	0.34	L_2
10[b]	5.27	5.05	0.22	L_2
10’[b]	5.15	4.98	0.17	L_2

[a]Calculation of the differences in chemical shifts $\Delta\delta^{RS}$ for relevant protons signals in the ^{1}H NMR of (+)-1D-**25** and (−)-1D-**25**. The absolute stereochemistry was determined using the Trost conformational model[38–40] and confirmed through single crystal X-ray diffraction as per the CIF..[b]The chemical shift (δ) values quoted were obtained from a ^{1}H NMR spectra, run at 400 MHz, in CH_3Cl. [c]For multiplets, the middle of the multiplet is quoted, if the peaks were not distinguishable, HSQC was employed to assign each chemical shift. Me = methyl; Ph = phenyl

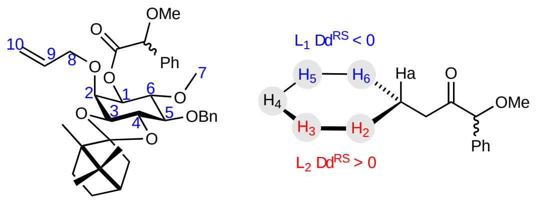

Supplementary Table 6. Reverse phase HPLC gradient for Method 1 and Method 2

Step length (min)	Elapsed time (min)	%A	%B
1	1	100	0
10	11	0	100
3	14	0	100
1	15	100	0
5	20	100	0

Supplementary Table 7. Normal phase HPLC gradient for Method 3

Step length (min)	Elapsed time (min)	%A	%B
1	1	95	5
12	13	5	95
5	18	5	95
1	19	95	5
6	25	95	5

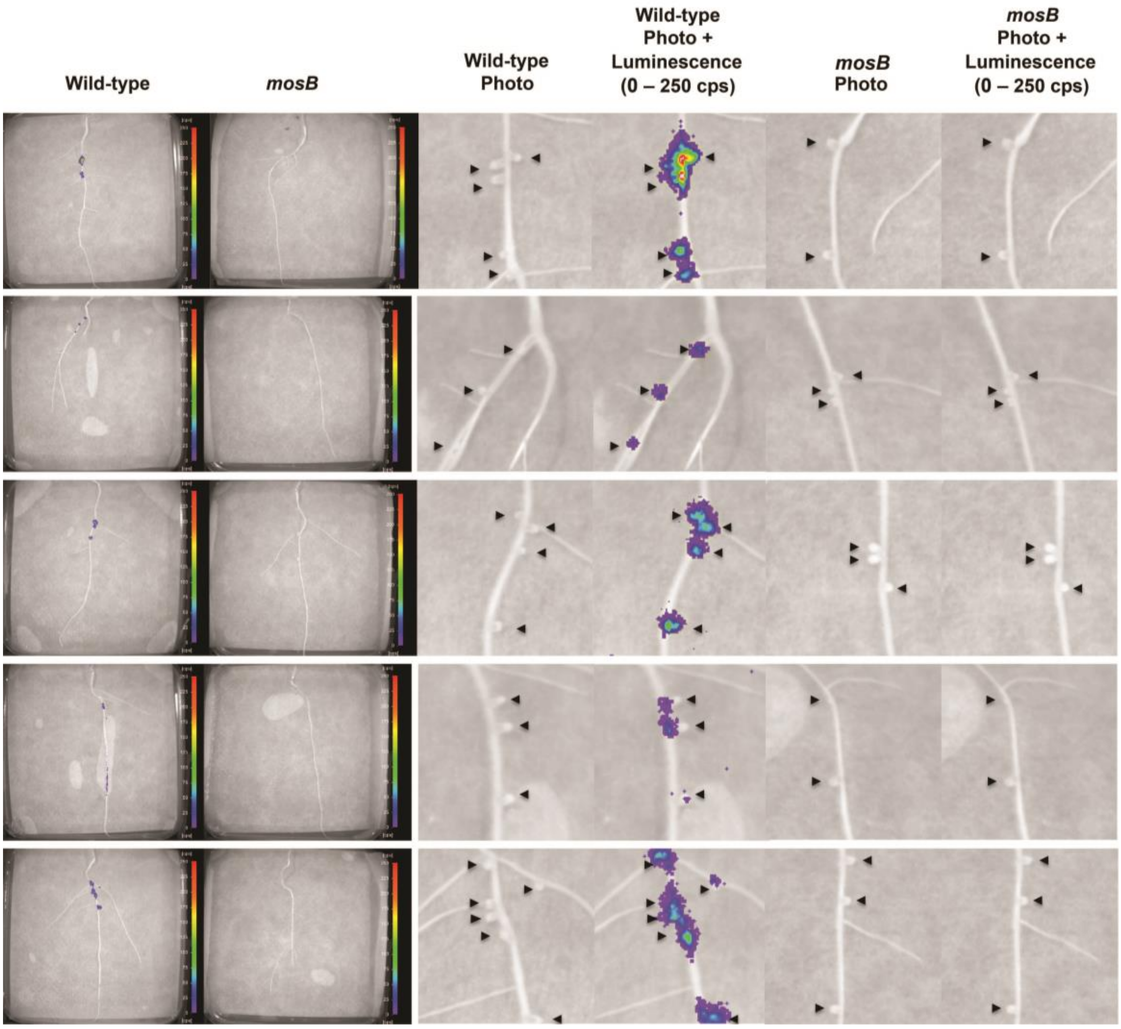

Supplementary Figure 1. Response of rhizopine lux biosensor to *Medicago sativa* rhizosphere. Bioluminescence response measured from *R. leguminosarum* Rlv3841/pOPS0046 rhizopine *lux* biosensor on the surface of *M. sativa* roots nodulated by *S. meliloti* L5-30 Wild-type (rhizopine +) and *S. meliloti* L5-30 *mosB*:pK19 (rhizopine –), see **Figure 2a**). NightOwl images are presented, with magnified root sections bearing nodules from five sets of plants (one representative set of plants presented in **Figure 1b**). Magnified images show photo only (left), or photo overlayed with luminescence response (right). Arrowheads indicate positions of nodules. Scale of all images is from 0 to 250 cps.

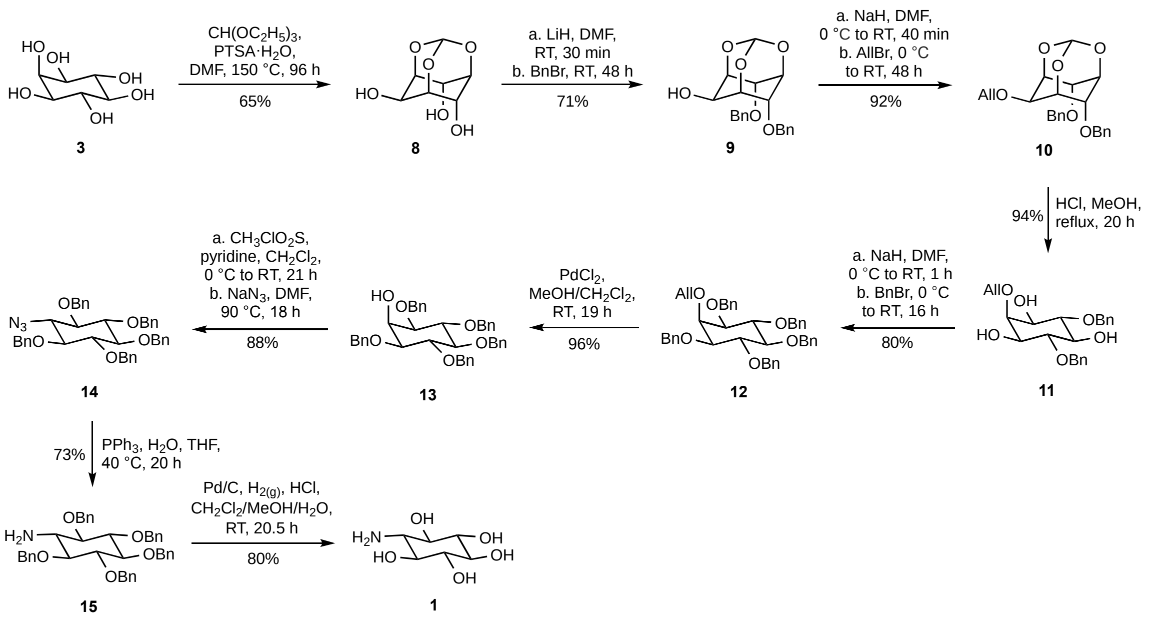

Supplementary Figure 2. Synthesis of SIA (1).

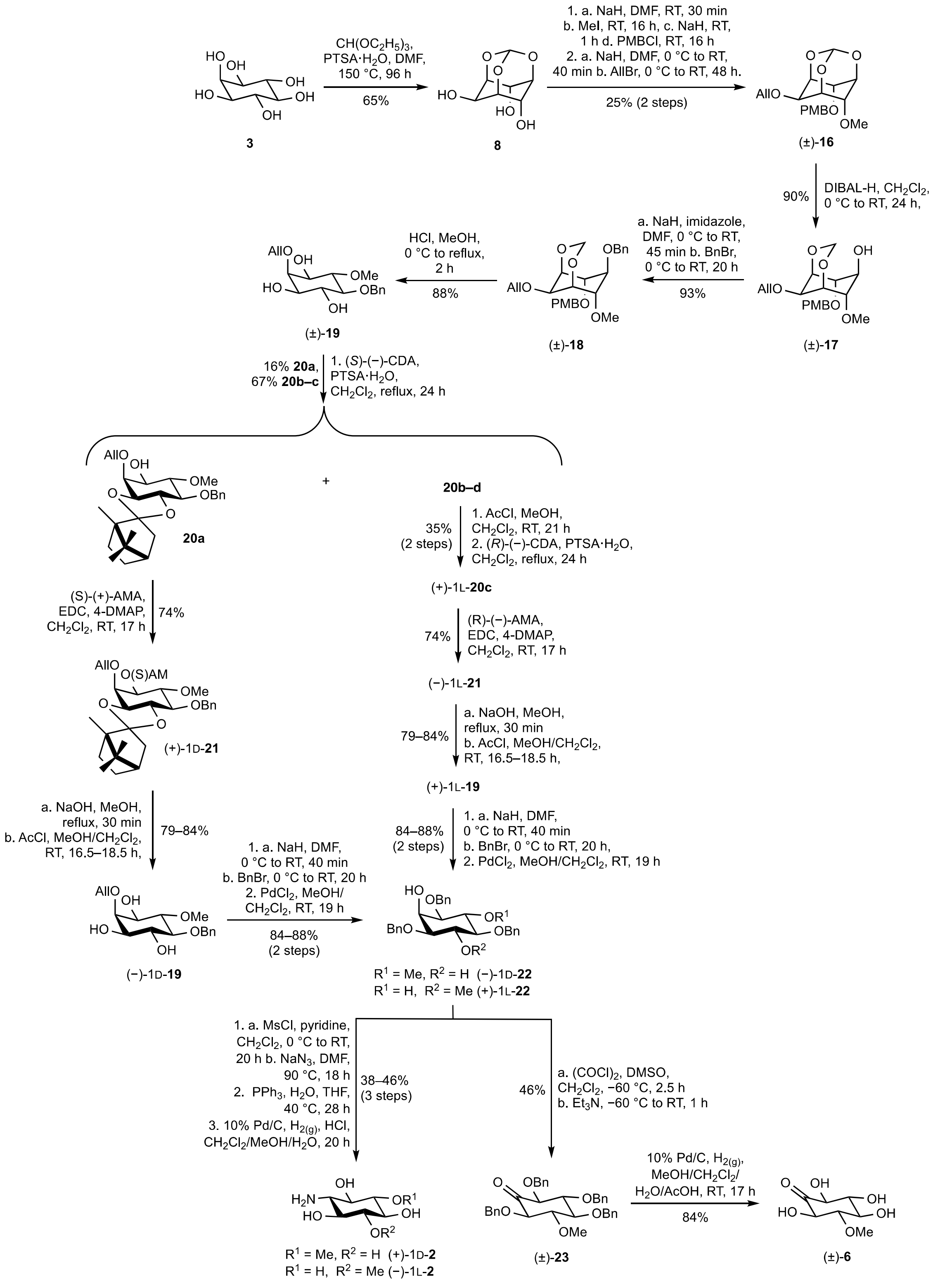

Supplementary Figure 3. Synthesis of both enantiomers of 3-*O*-MSI (+)-1D-2 and (+)-1L-2 and racemic 3-*O*-methyl-*scyllo*-inosose 6. All = allyl, Bn = benzyl; Me = methyl; Ac = acetate; AMA = acetylmandelic acid; PMB = 4-methoxybenzyl; CDA = Camphor dimethyl acetal.

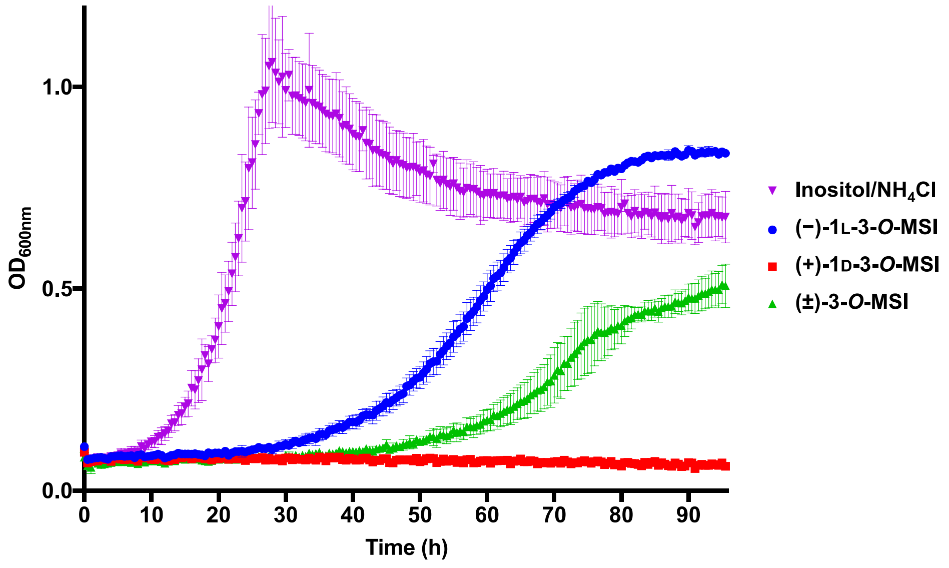

Supplementary Figure 4. Growth of *S. meliloti* L5-30 using chemically synthesised rhizopines. Growth of *S. meliloti* L5-30 measured by OD^{600} in UMS minimal media. Media contained either 10 mM *myo*-inositol **3** and 10 mM NH_4Cl, 10 mM enantiomerically pure or 10 mM racemic 3-*O*-MSI **2** as sole carbon and nitrogen sources. Error bars represent standard deviation of three independent replicates.

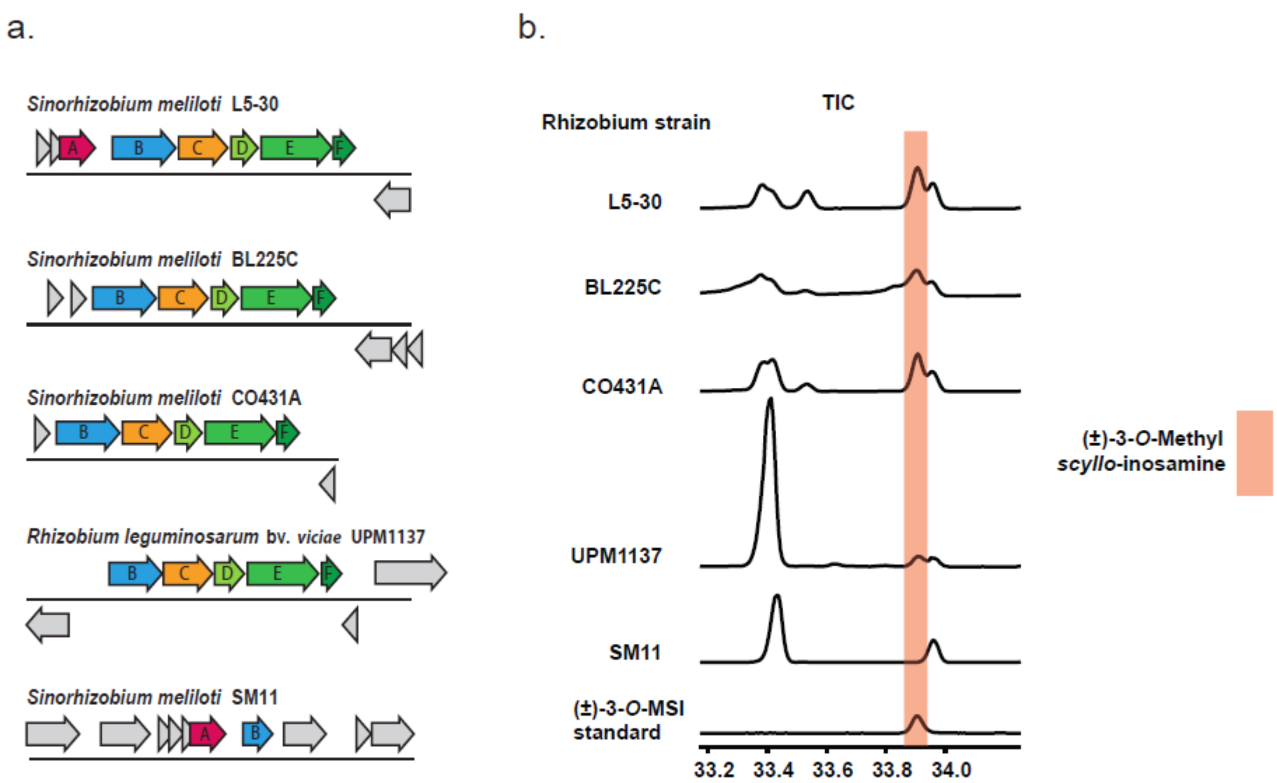

Supplementary Figure 5. Putative rhizopine loci in rhizobia.

a. Structure of putative rhizopine loci with MosB homologues in rhizobia with sequenced genomes. **b.** GC-MS TIC chromatograms from nodules formed by rhizobia with putative rhizopine loci. (±)-3-*O*-methyl scyllo-inosamine (±)-2 (red) production was observed based on comparison with a chemical standard. SIA **1** production was not observed. Source data of Supplementary Figure 5b are provided as a Source Data file.

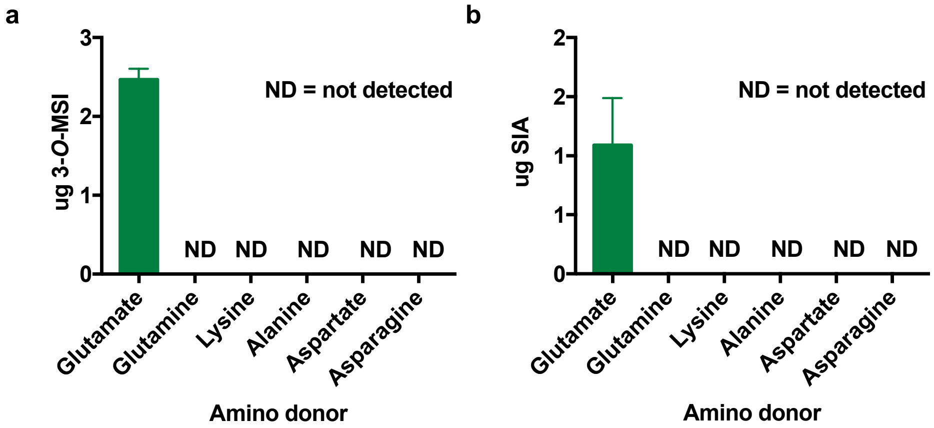

Supplementary Figure 6. Glutamate serves as amino donor to MosB.

a. Quantification of 3-*O*-MSI **2** production from (±)-3-*O*-methyl-scyllo-inosose (±)-**6** by MosB in vitro assay with different amino donors **b.** Quantification of SIA **1** production from myo-inositol by IolG + MosB linked in vitro assay with different amino donors. Error bars represent standard deviation of three independent replicates.

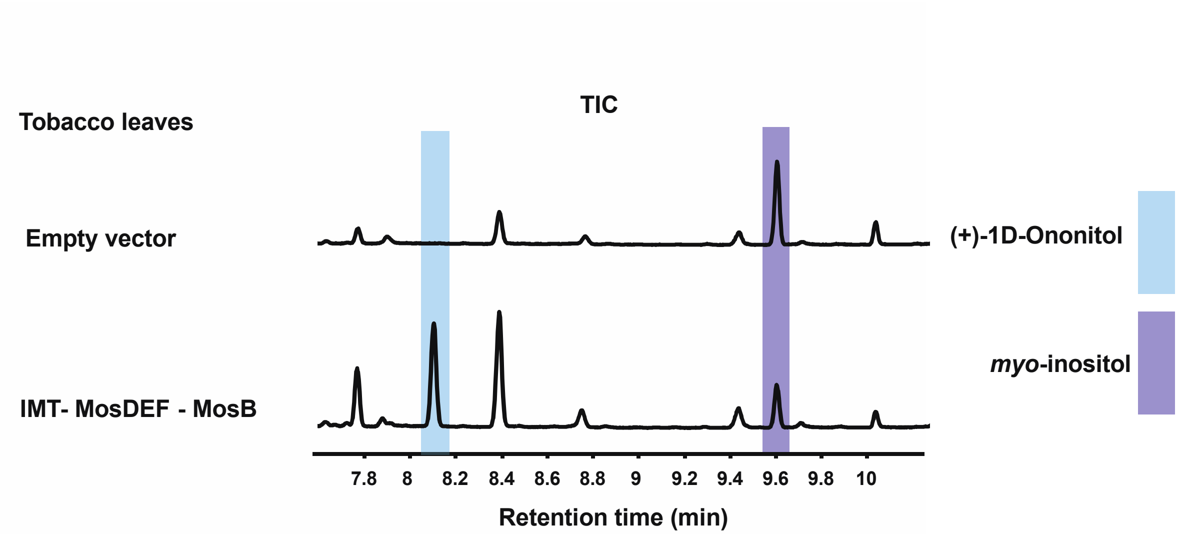

Supplementary Figure 7. Transient expression of 3-O-MSI biosynthesis pathway in *N. benthamiana leaves*.

GC-MS Chromatograms of extracts prepared from tobacco leaves agro-infiltrated with either empty vector (control) or IMT-MosDEF-MosB together. TIC, total ion chromatograms; (+)-1D-ononitol **4** (light blue); *myo*-inositol **3** (dark blue). The chromatograms shown are representative of experiments repeated at least three independent times. Source data are provided as a Source Data file.

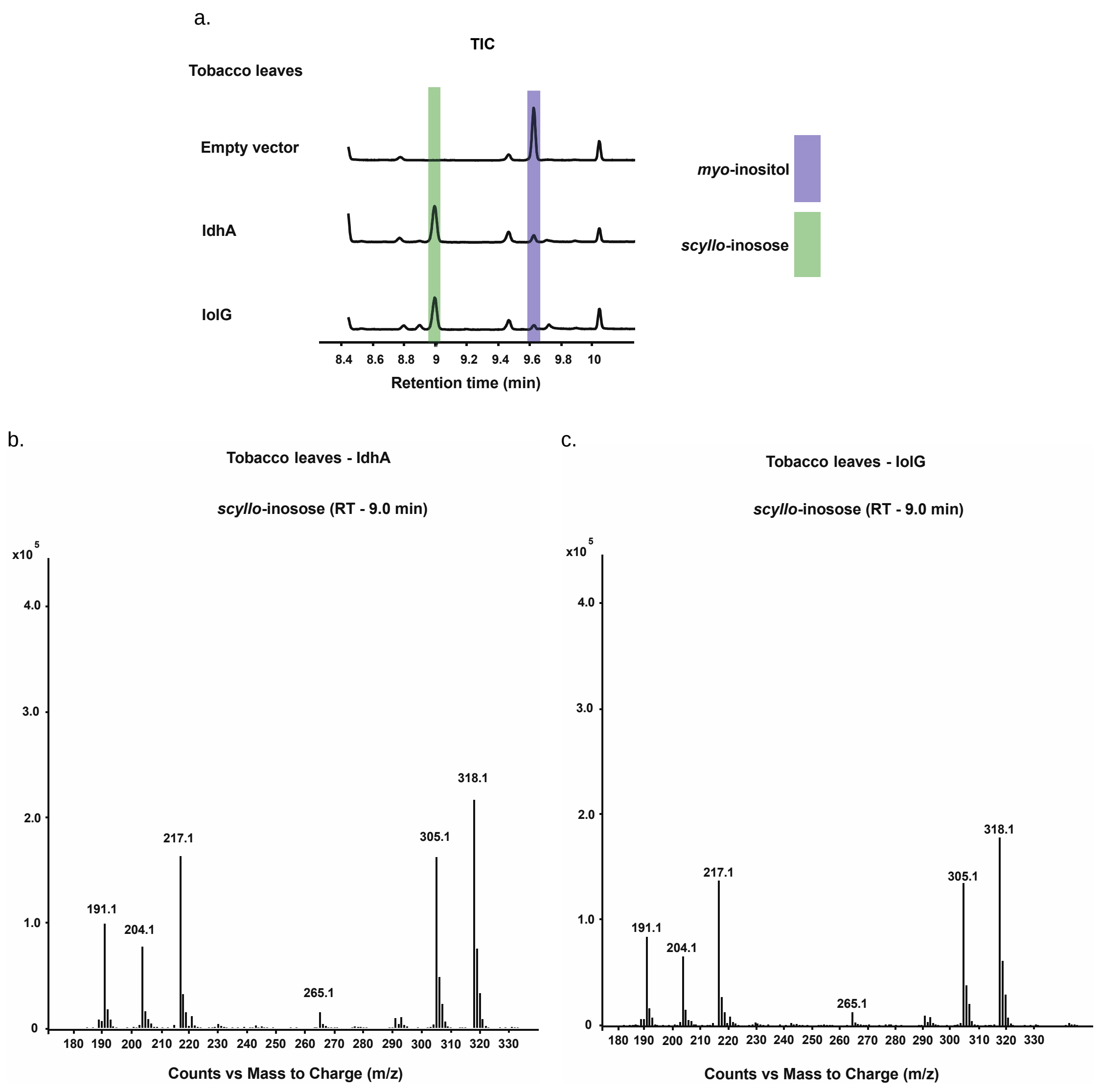

Supplementary Figure 8. Transient expression of inositol dehydrogenases in *N. benthamiana* leaves.

a. GC-MS Chromatograms of extracts prepared from tobacco leaves agro-infiltrated with either empty vector (control) or IdhA or IolG. TIC, total ion chromatograms; *scyllo*-inosose **7** (green); *myo*-inositol **3** (dark blue). All chromatograms are representative of experiments repeated at least two independent times. **b-c.** Mass spectra of *scyllo*-inosose **7** produced in tobacco leaves agro-infiltrated with either IdhA (**b**) or IolG (**c**). Source data of Supplementary Figure 8a are provided as a Source Data file.

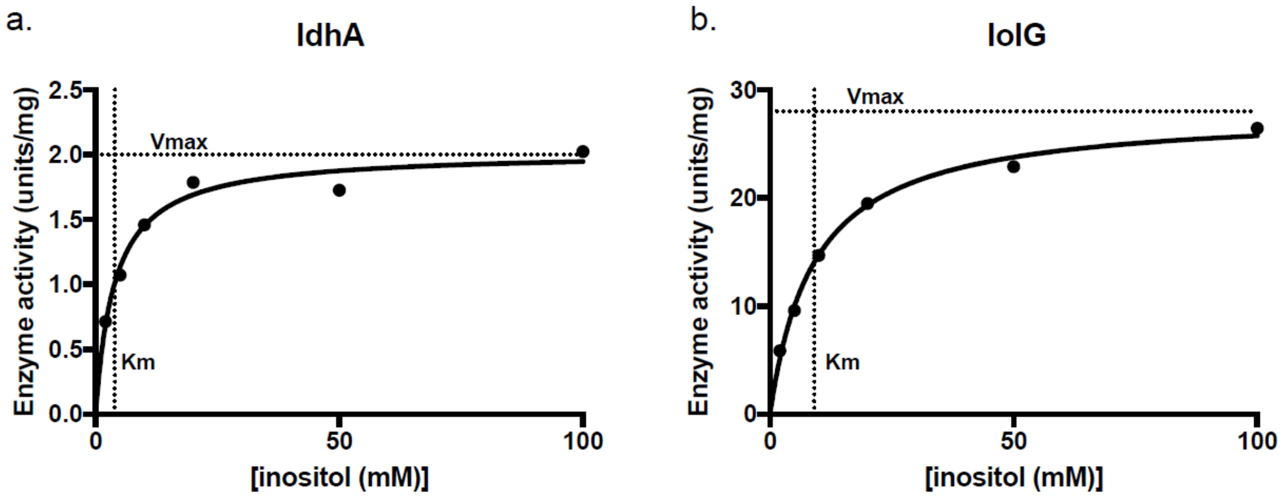

Supplementary Figure 9. Enzyme activity of purified inositol dehydrogenases

Enzyme activity of inositol dehydrogenase from *Rhizobium leguminosarum* (IdhA) (**a**) or *Bacillus subtilis* (IolG) (**b**) was measured by NAD^+-linked inositol dehydrogenase assay with *myo*-inositol as a substrate as previously described[41].

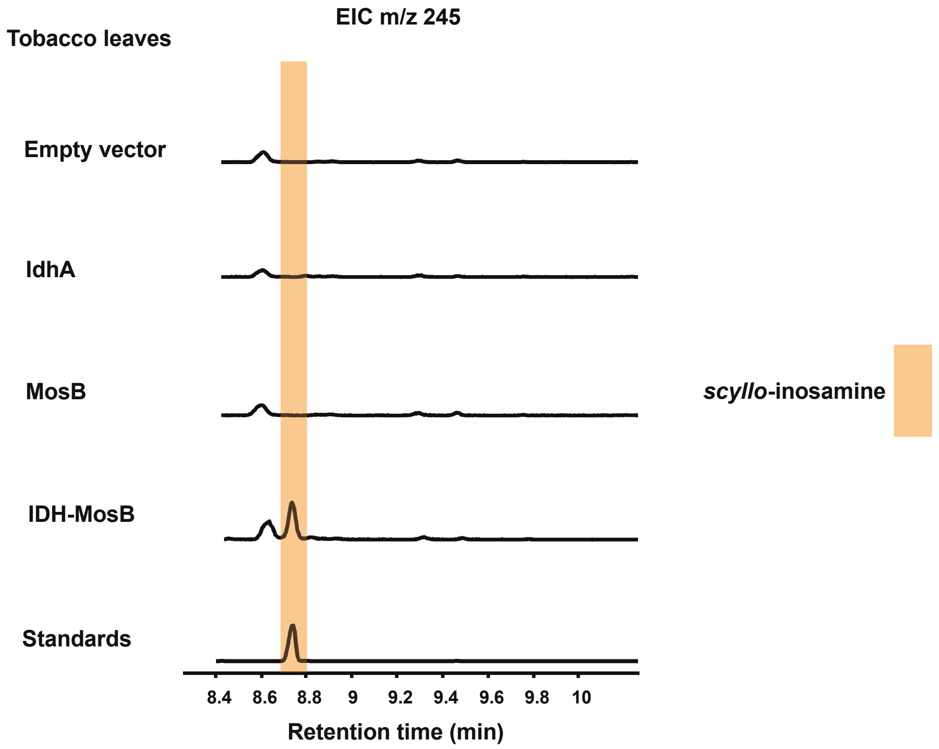

Supplementary Figure 10. EIC[245] chromatograms from transient expression of SIA biosynthesis pathway in *N. benthamiana* leaves.

GC-MS Chromatograms of extracts prepared from tobacco leaves agro-infiltrated with either empty vector (control) or IdhA or MosB or IdhA-MosB together. EIC (m/z 245), Extracted ion chromatogram (mass to charge ratio); *scyllo*-inosamine **1** (orange). All chromatograms are representative of experiments repeated at least three independent times.5t

a.

b.

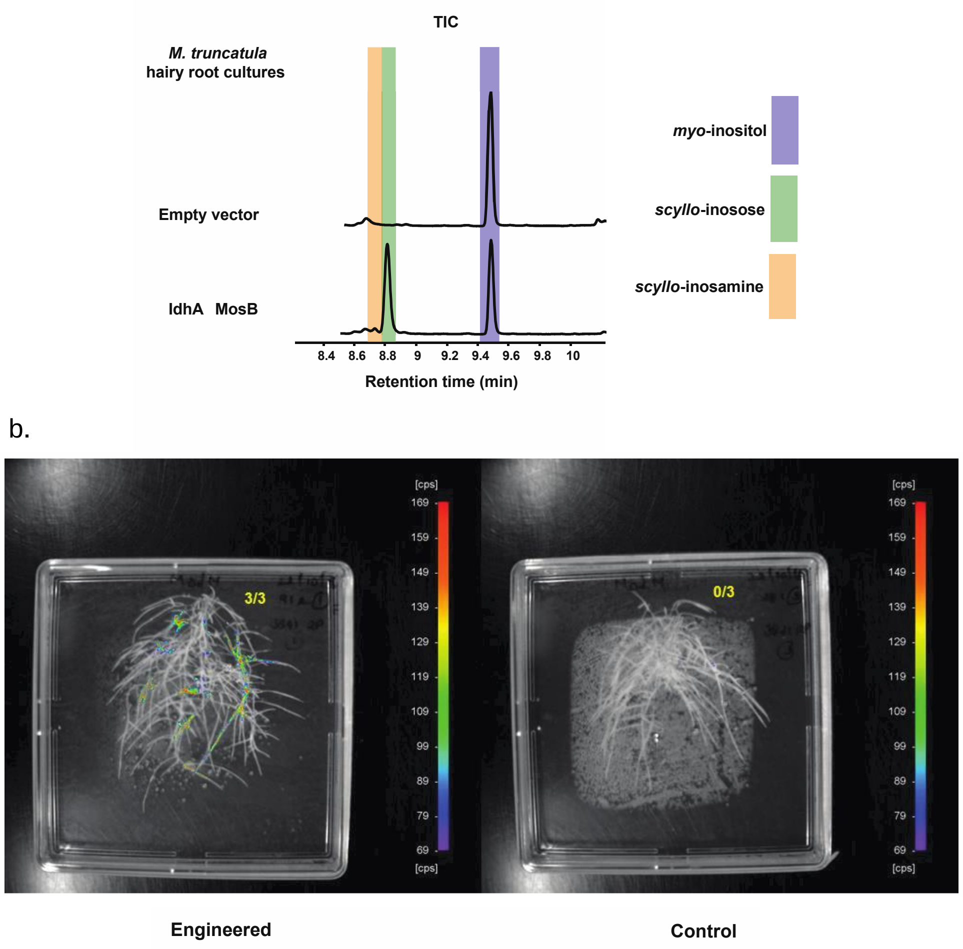

Supplementary Figure 11. Rhizopine mediated transkingdom signalling in *M. truncatula* root organ culture expressing synthetic SIA biosynthesis pathway.

a. GC-MS total ion chromatograms (TIC) of extracts prepared from M. truncatula transgenic hairy root cultures transformed with empty vector (control) or IdhA-MosB together. *scyllo*-inosamine **1** (orange); scyllo-inosose **7** (green); *myo*-inositol **3** (dark blue). All chromatograms are representative of experiments repeated at least three independent times. **b.** NightOwl images showing bioluminescence of Rlv3841/pOPS0046 rhizopine lux biosensor on the surface of hairy root cultures transformed with empty vector (control) or IdhA-MosB together (engineered). Numbers in top right corners indicate number of plants tested that showed significant levels of bioluminescence. Experiments were repeated at least three independent times. Source data of Supplementary Figure 11a are provided as a Source Data file.

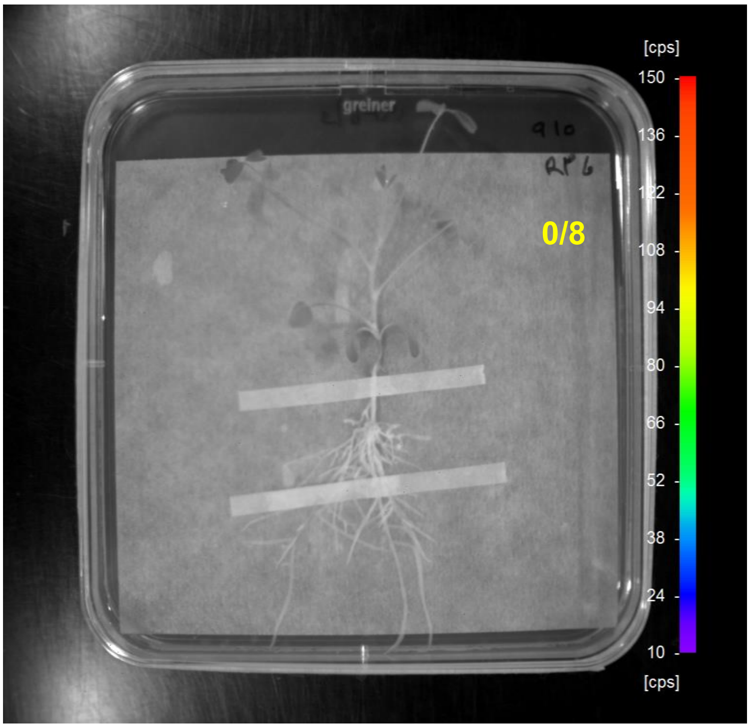

Engineered

Supplementary Figure 12. Rhizopine lux biosensor response to *M. truncatula* roots expressing inositol dehydrogenase alone.

NightOwl images showing bioluminescence of Rlv3841/pOPS0046 rhizopine *lux* biosensor on the surface of *M. truncatula* transgenic roots transformed with IdhA alone (Engineered). Numbers in top right corners indicate number of plants tested. Experiments were repeated at least two independent times.

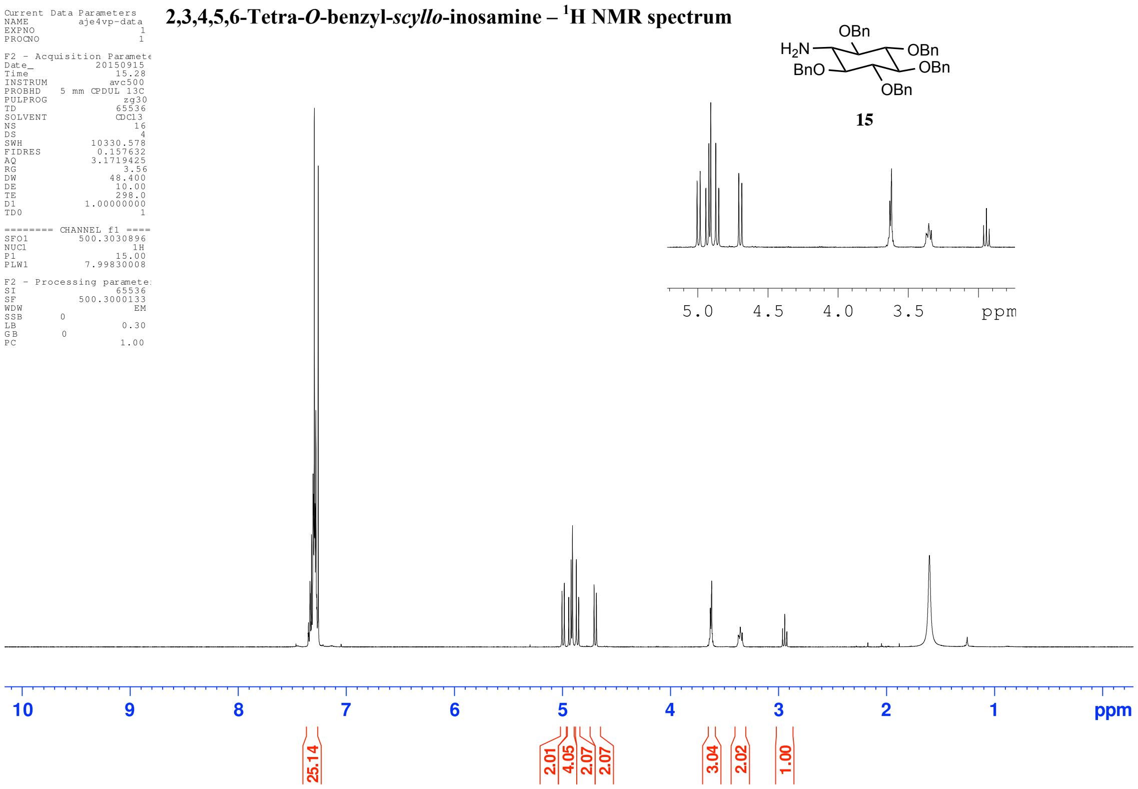

Supplementary Figure 13 – ^{1}H NMR (500 MHz; $CDCl_3$) of compound 15

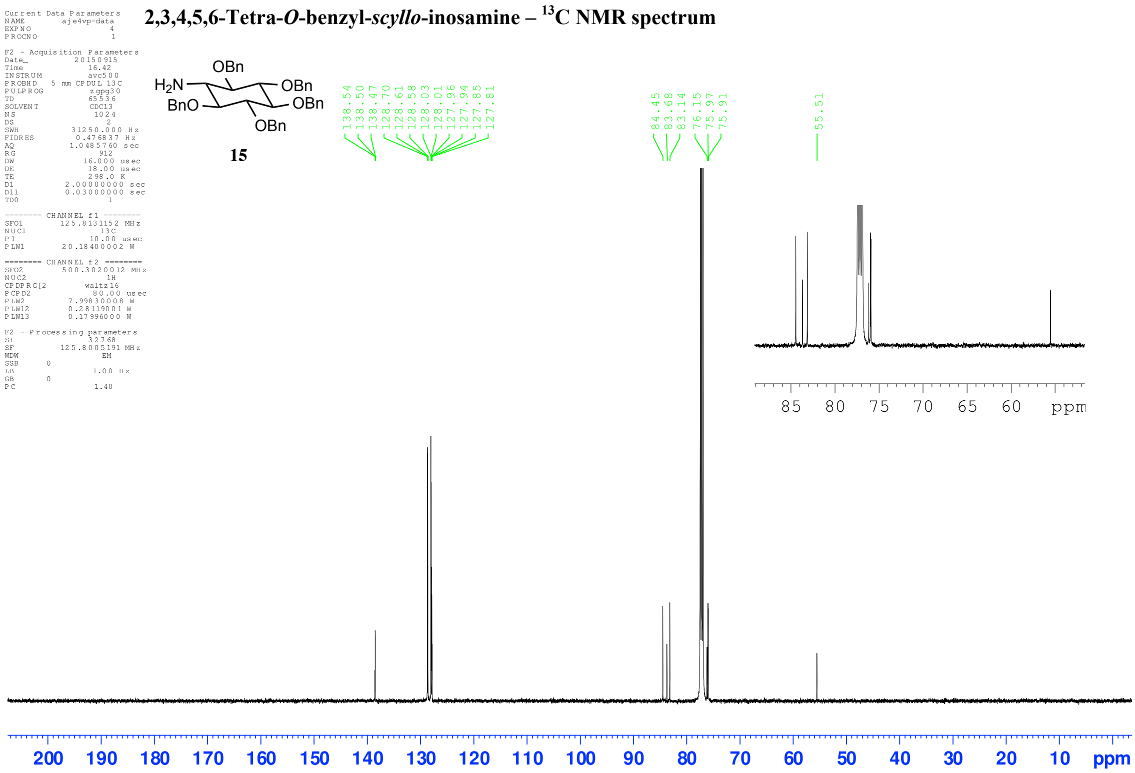

Supplementary Figure 14 – ^{13}C NMR (126 MHz; $CDCl_3$) of compound 15

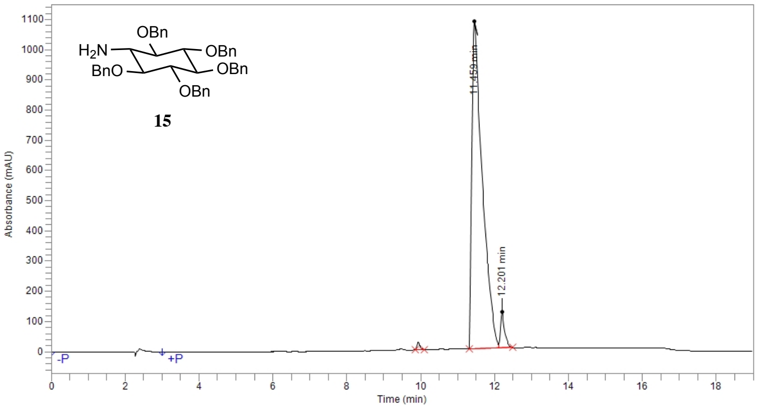

Supplementary Figure 15 – HPLC trace of compound 15

Method 1, t = 11. 459 min, 95.25%.

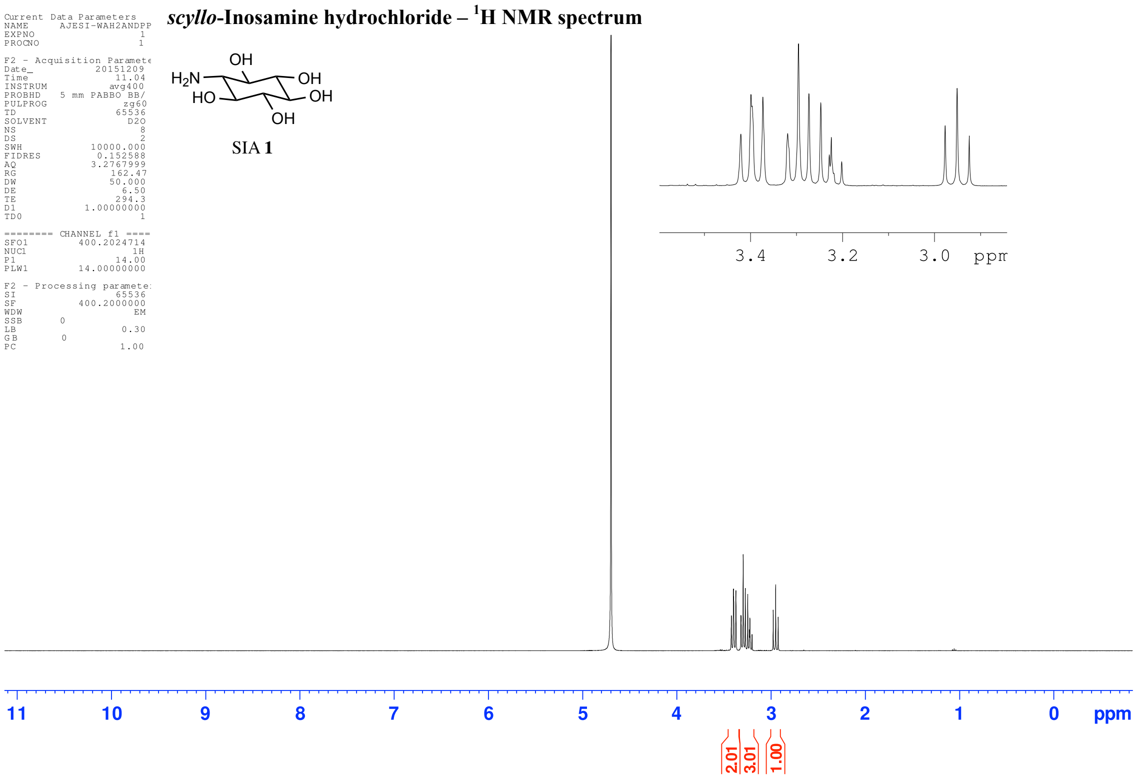

Supplementary Figure 16 – ^{1}H NMR (400 MHz; D_2O) of compound 1

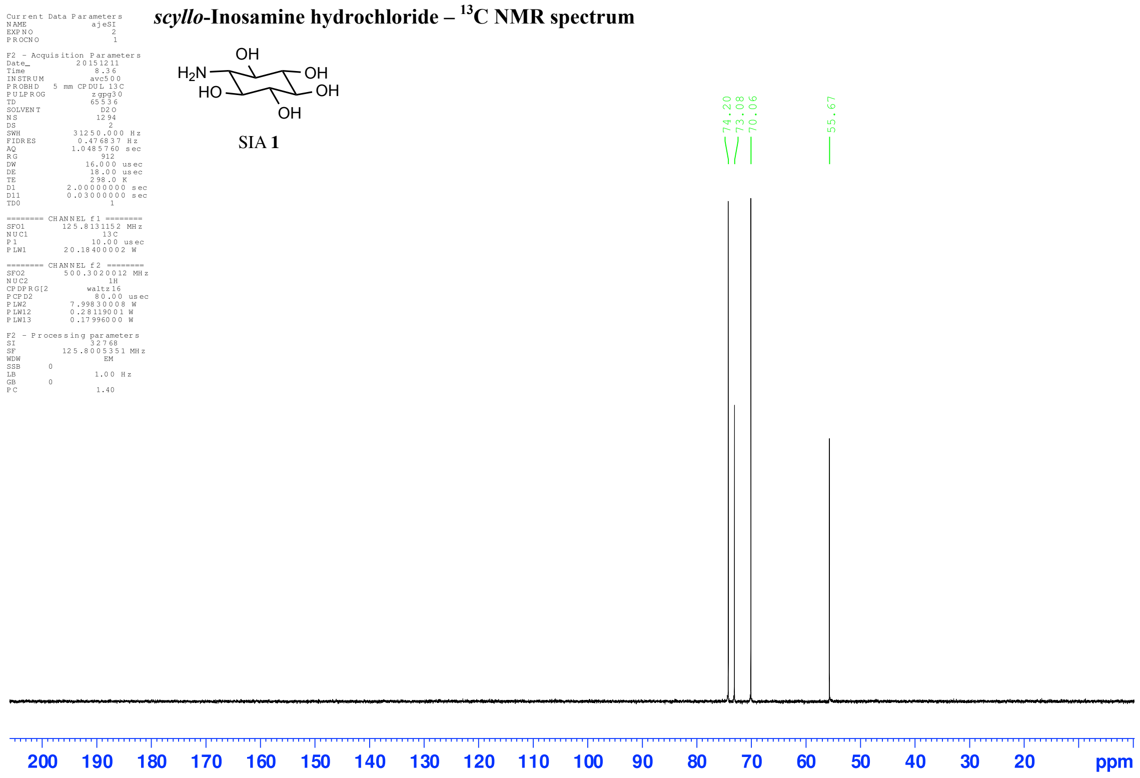

Supplementary Figure 17 – ^{13}C NMR (126 MHz; D_2O) of compound 1

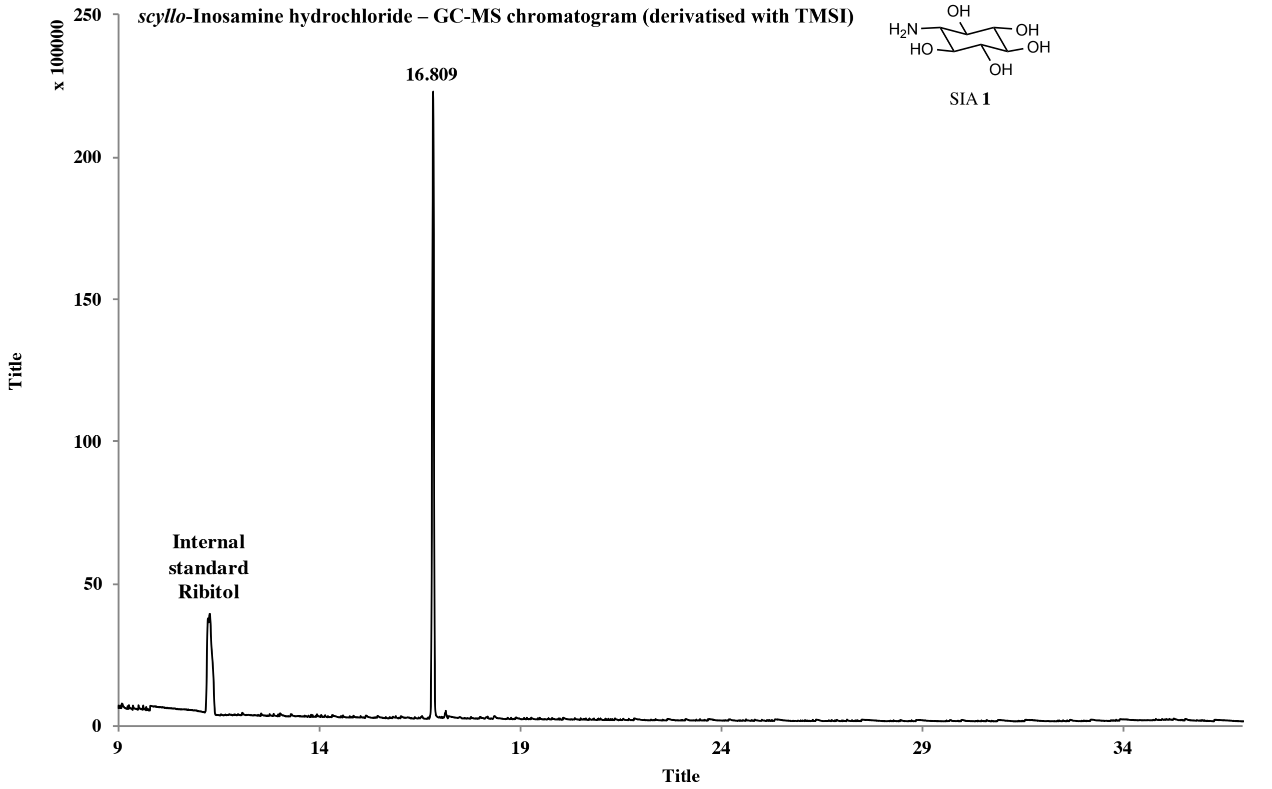

Supplementary Figure 18 – GS-MS chromatogram of compound 1 (t = 16.8 min, 100%)

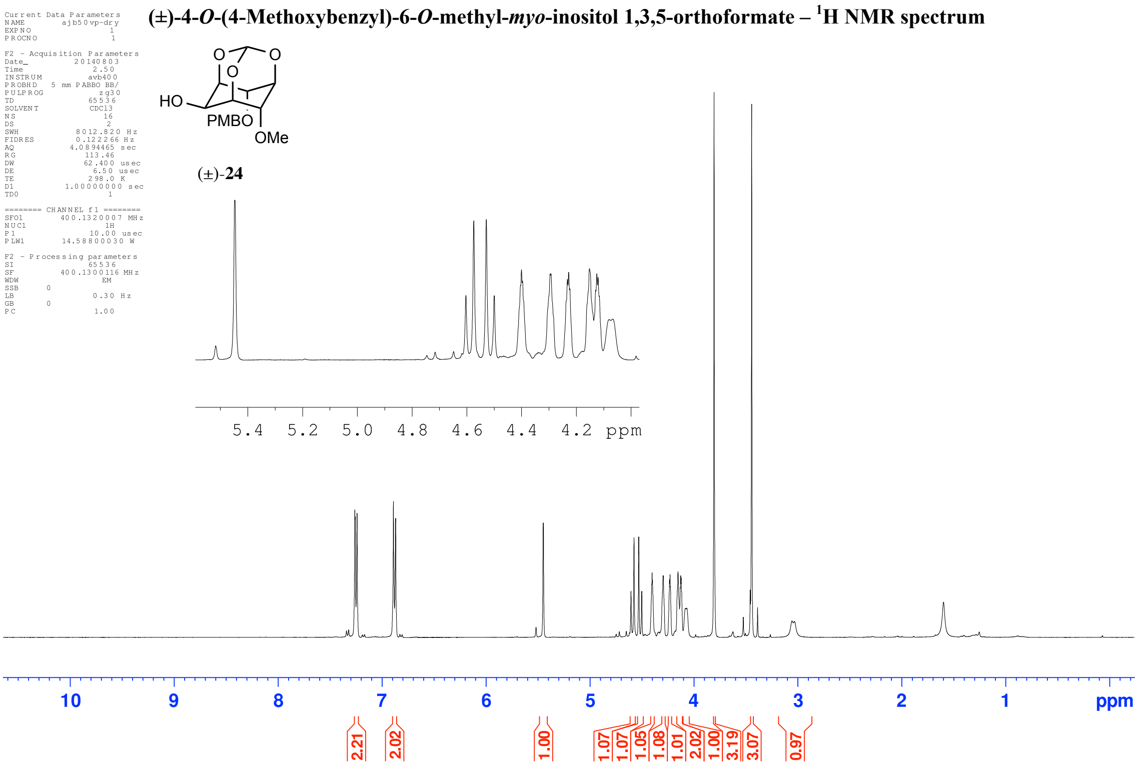

Supplementary Figure 19 – ^{1}H NMR (400 MHz; $CDCl_3$) of compound (±)-24

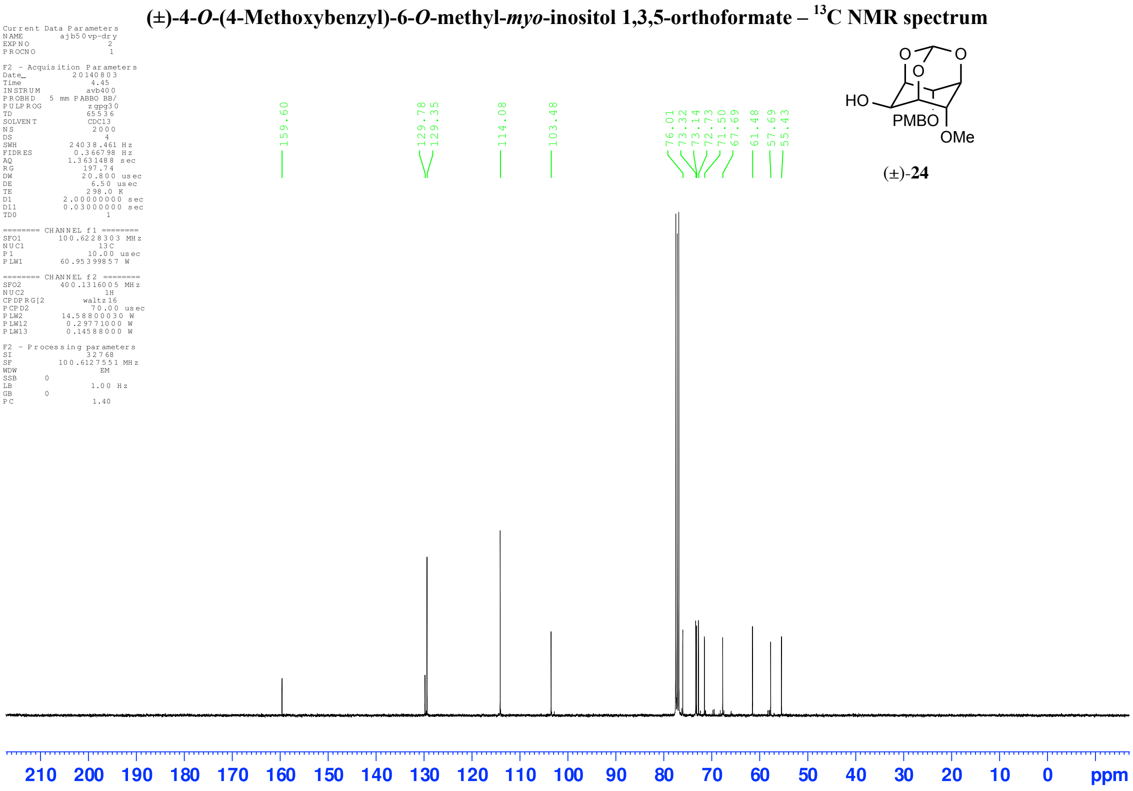

Supplementary Figure 20 – ^{13}C NMR (101 MHz; $CDCl_3$) of compound (±)-24

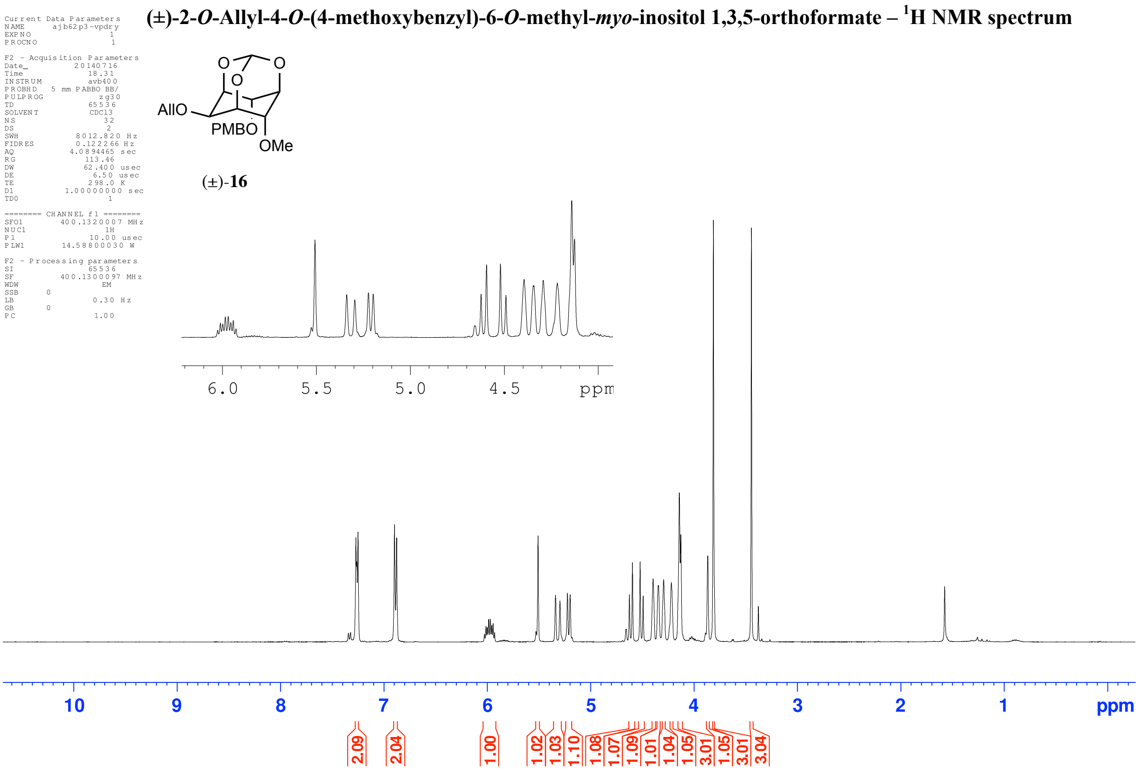

Supplementary Figure 21 – ^{1}H NMR (400 MHz; $CDCl_3$) of compound (±)-16

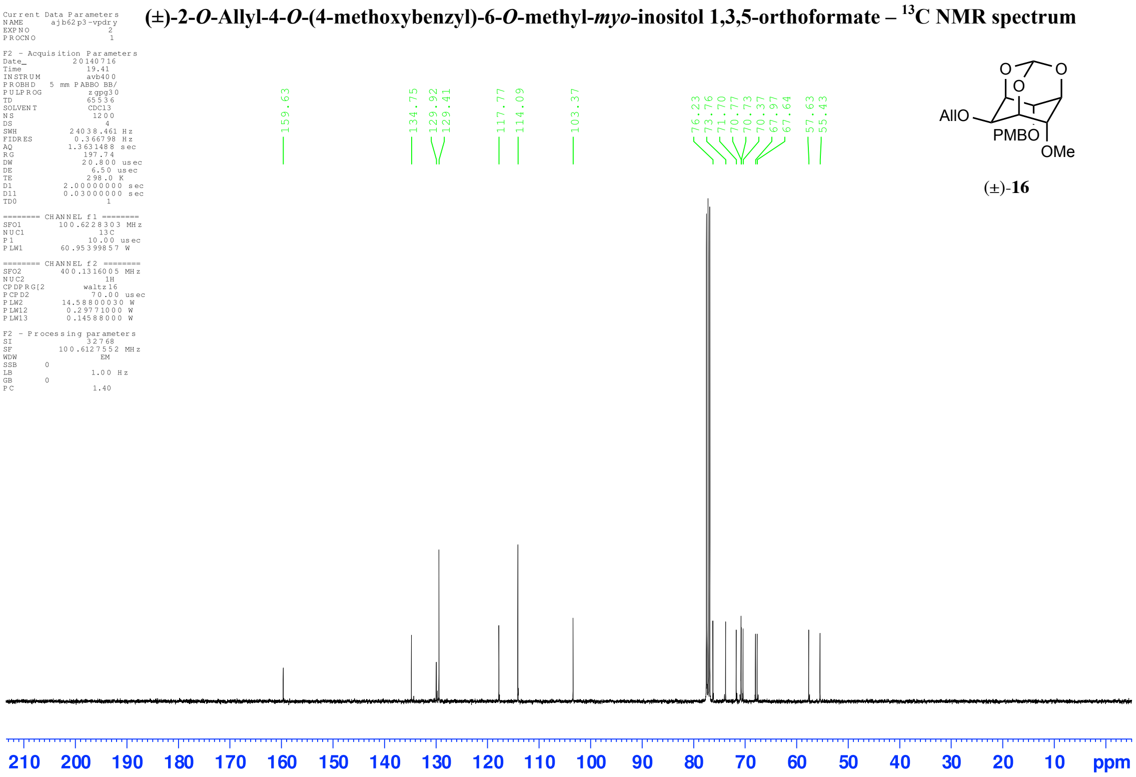

Supplementary Figure 22 – ^{13}C NMR (101 MHz; $CDCl_3$) of compound (±)-16

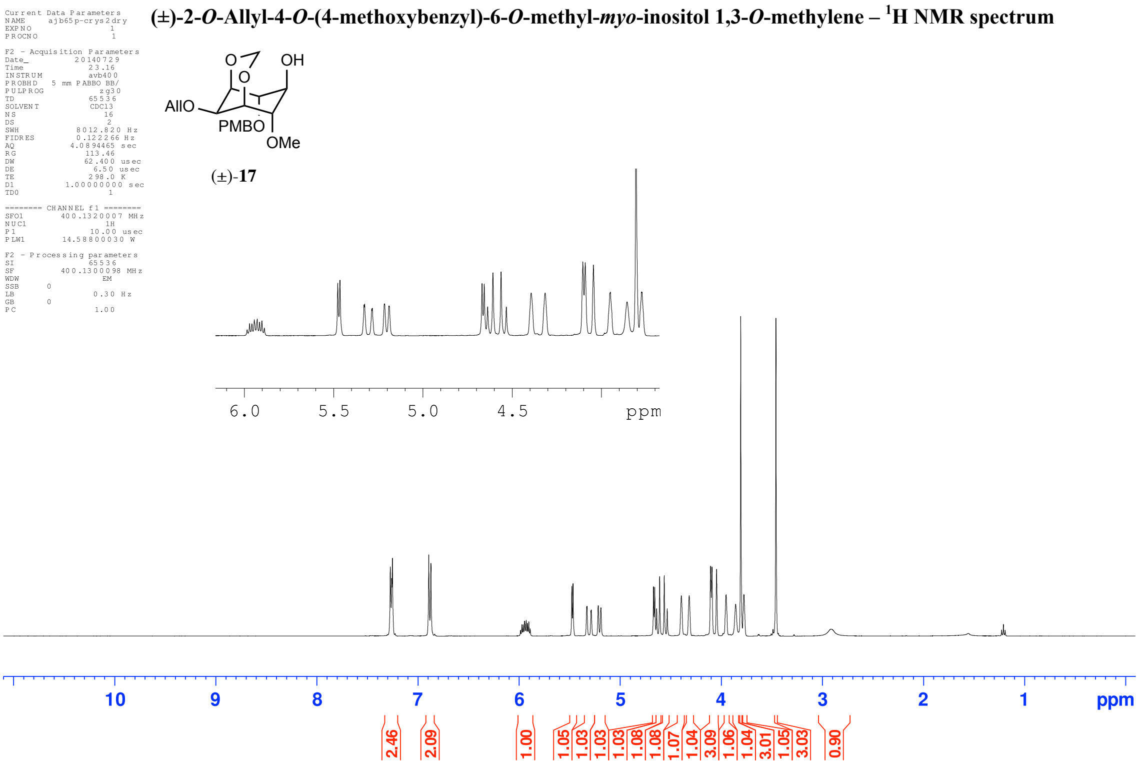

Supplementary Figure 23 – ^{1}H NMR (400 MHz; $CDCl_3$) of compound (±)-17

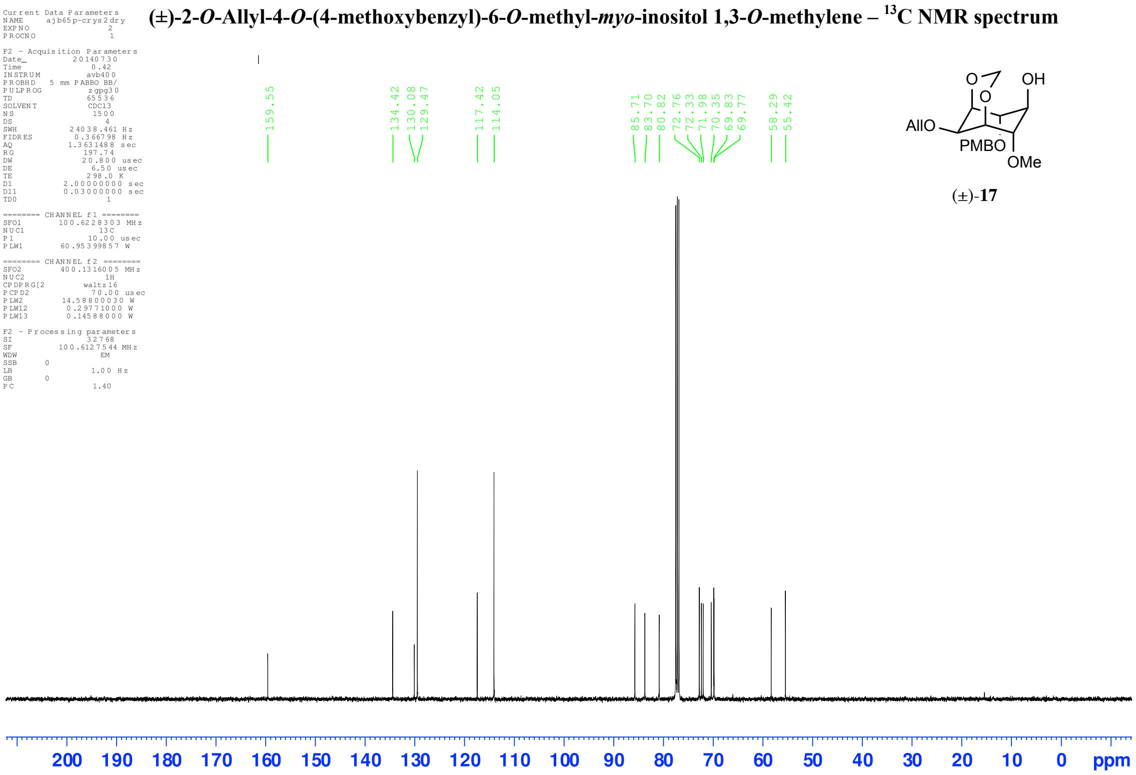

Supplementary Figure 24 – ^{13}C NMR (101 MHz; $CDCl_3$) of compound (±)-17

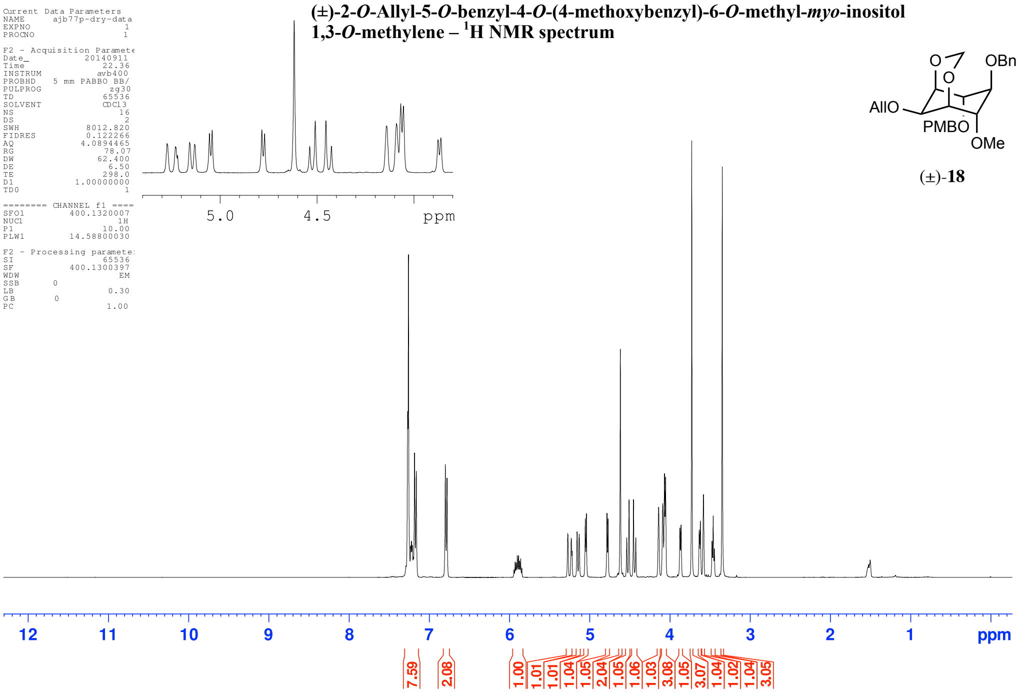

Supplementary Figure 25 – ^{1}H NMR (400 MHz; $CDCl_3$) of compound (±)-18

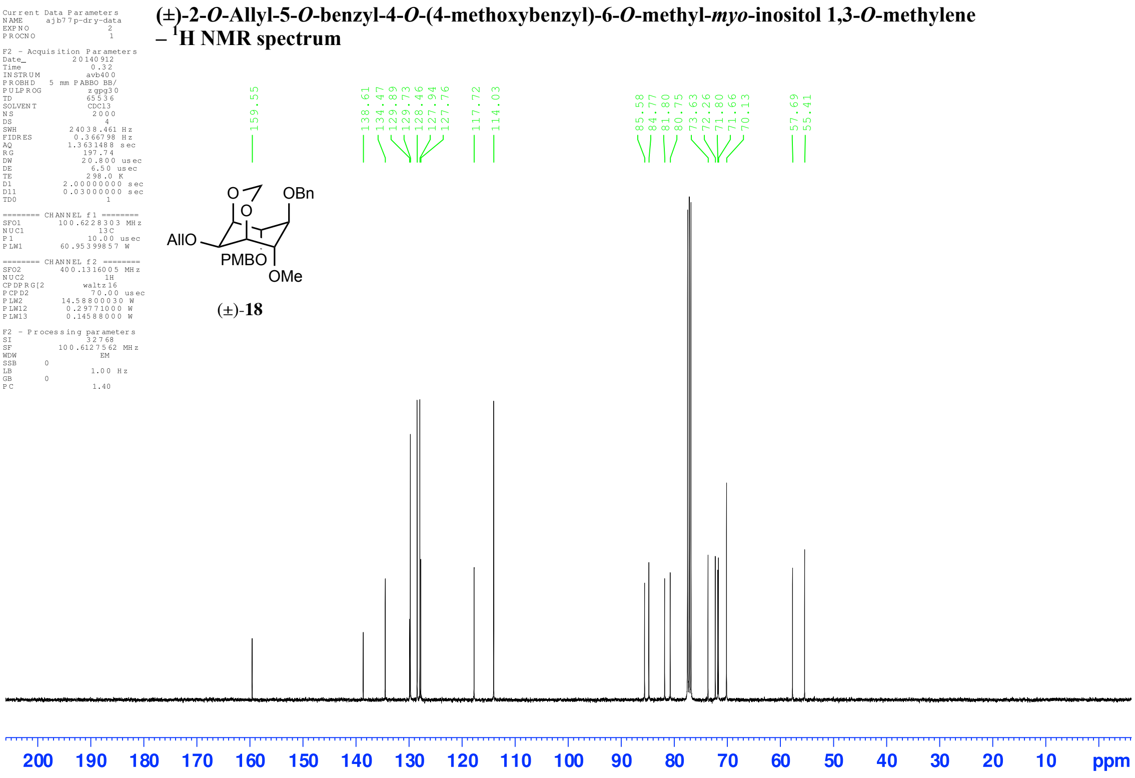

Supplementary Figure 26 – ^{13}C NMR (101 MHz; $CDCl_3$) of compound (±)-18

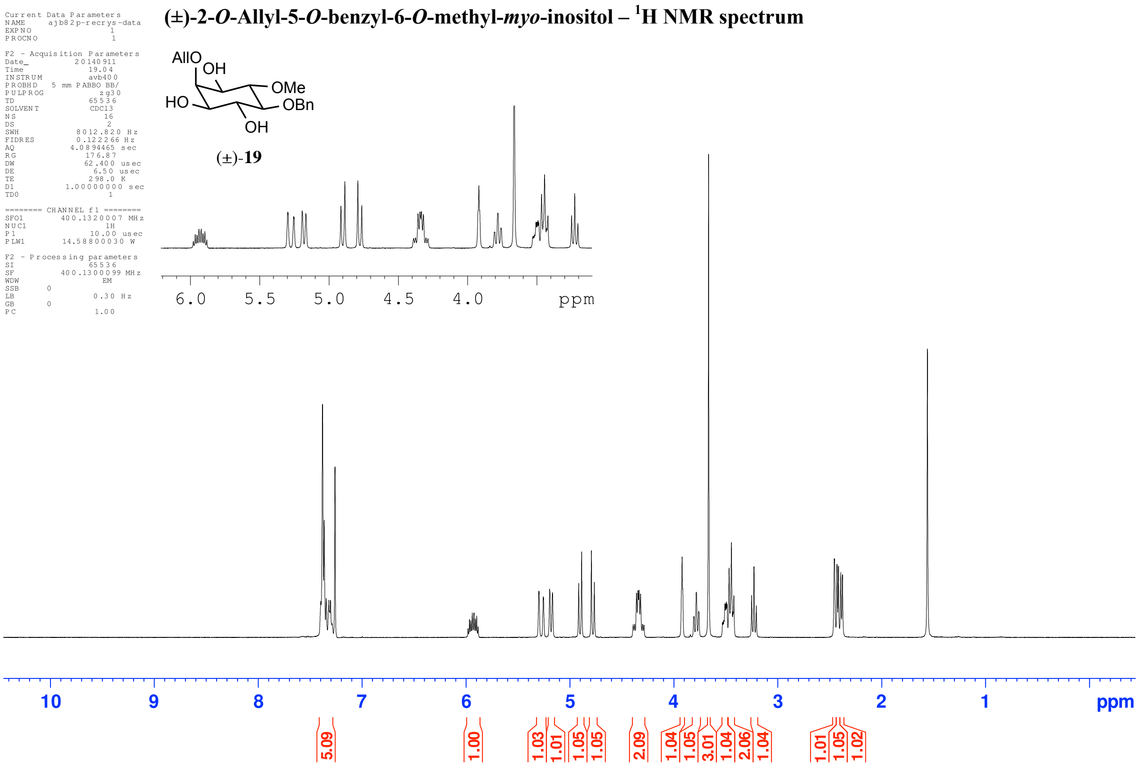

Supplementary Figure 27 – ^{1}H NMR (400 MHz; $CDCl_3$) of compound (±)-19

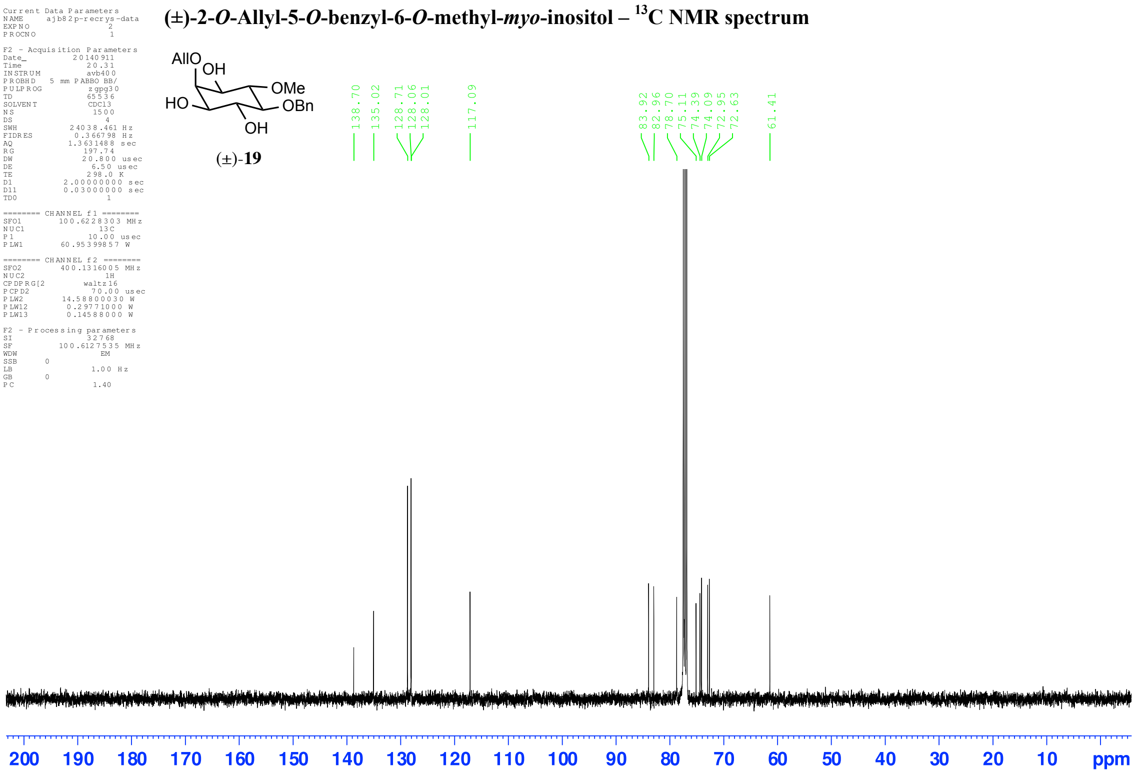

Supplementary Figure 28 – ^{13}C NMR (101 MHz; $CDCl_3$) of compound (±)-19

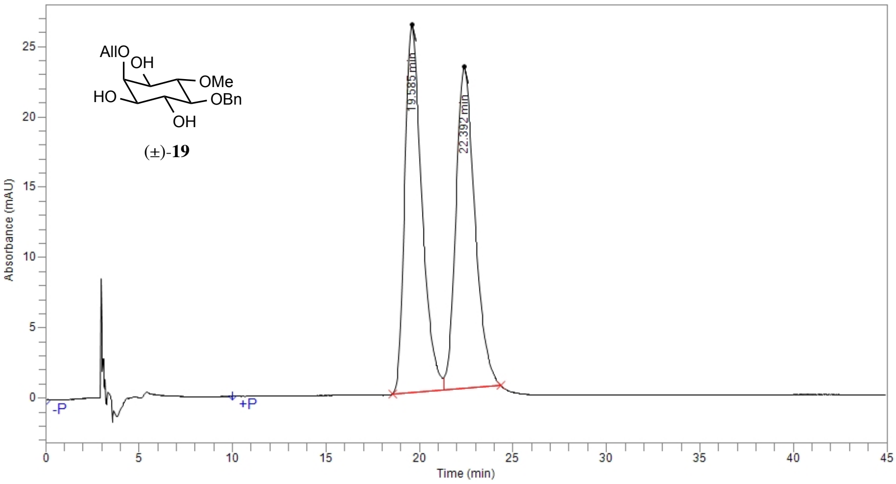

Supplementary Figure 29 – Chiral HPLC of compound (±)-19.
Heptane/IPA 90:10, 0.8 mL.min-1, 254 nm, t = 19. 585 min, 50.49%; t = 22.392, 49.51%.

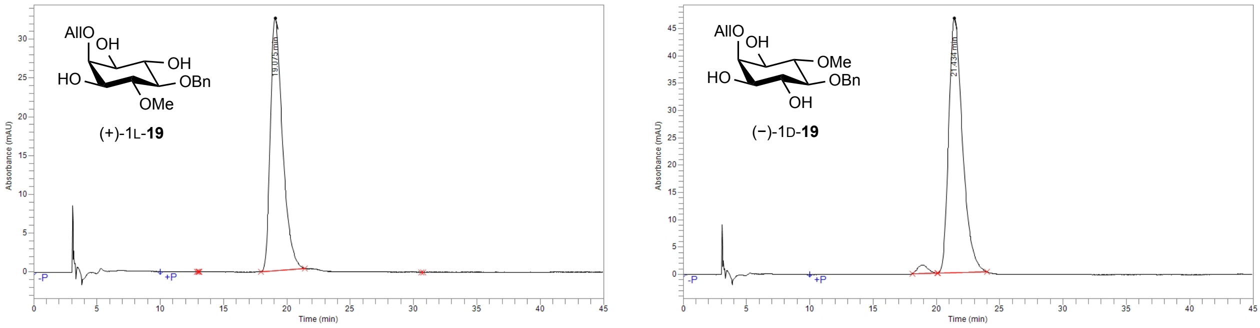

Supplementary Figure 30 – Chiral HPLC of compounds (+)-19 and (−)-19.
Heptane/IPA 90:10, 0.8 mL.min-1, 254 nm, t = 19. 585 min, 50.49%; t = 22.392, 49.51%.

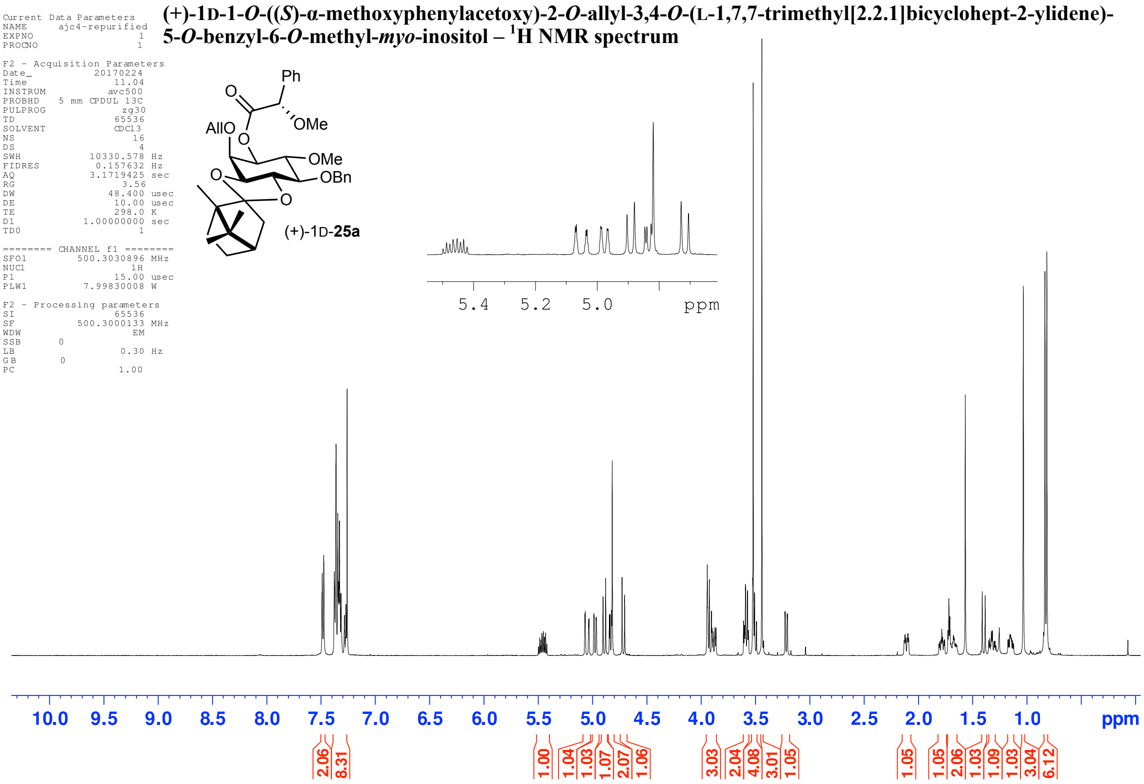

Supplementary Figure 31 – ^{1}H NMR (500 MHz; $CDCl_3$) of compound (+)-1D-25a

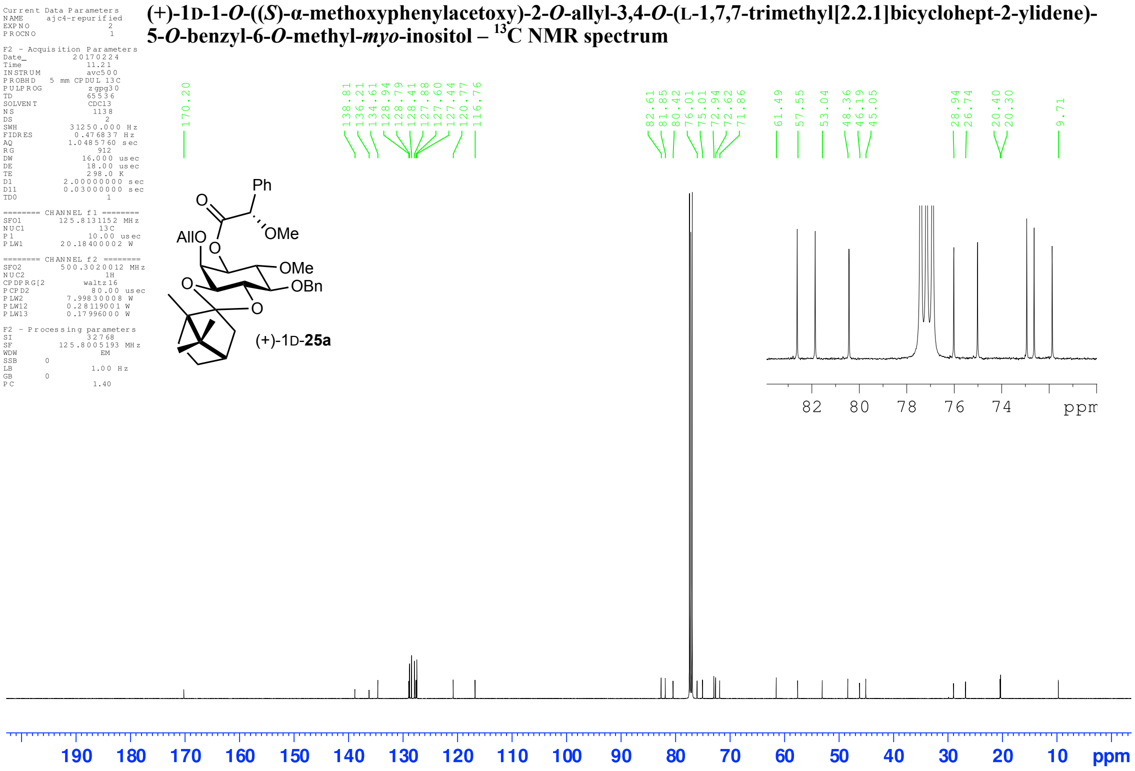

Supplementary Figure 32 – ^{13}C NMR (126 MHz; $CDCl_3$) of compound (+)-1D-25a

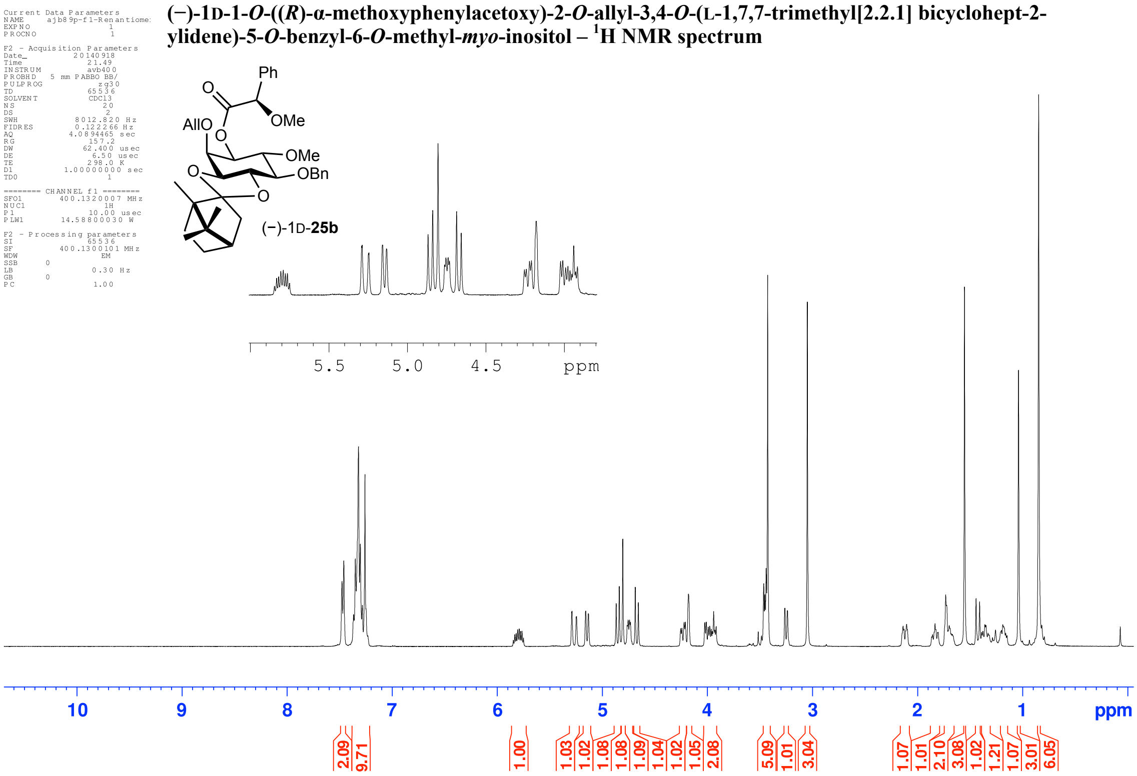

Supplementary Figure 33 – ^{1}H NMR (400 MHz; $CDCl_3$) of compound (−)-1D-25b

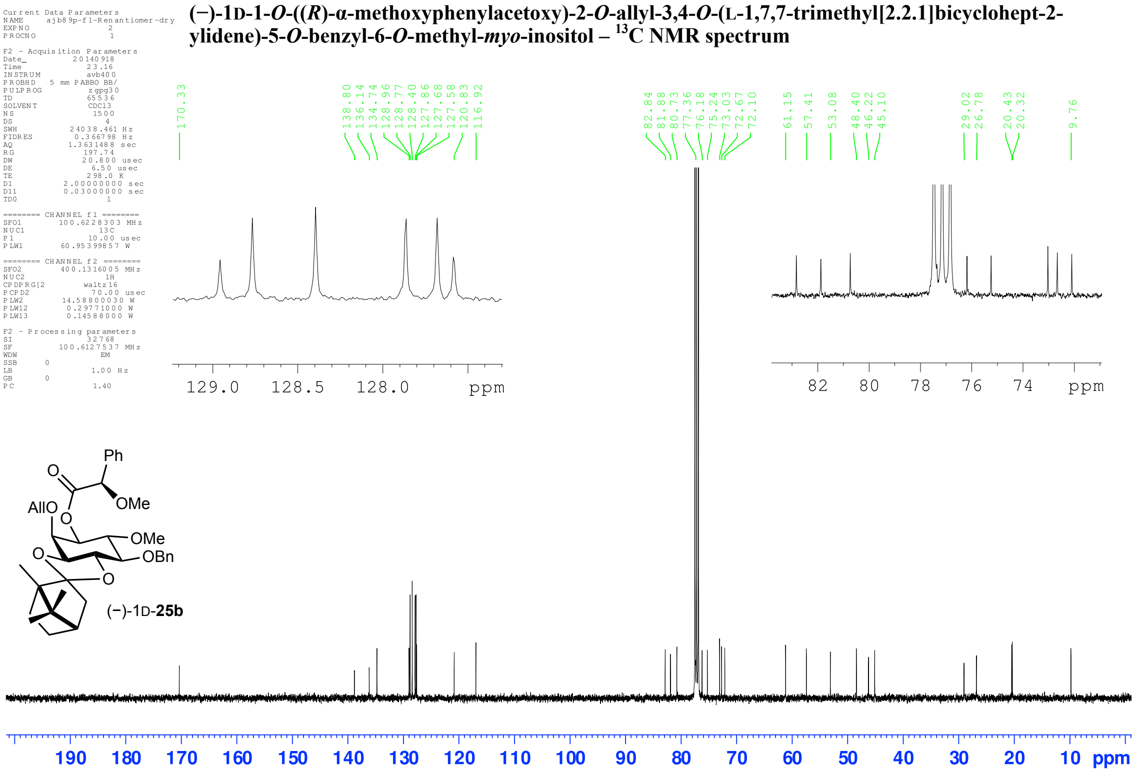

Supplementary Figure 34 – ^{13}C NMR (101 MHz; $CDCl_3$) of compound (−)-1D-25b

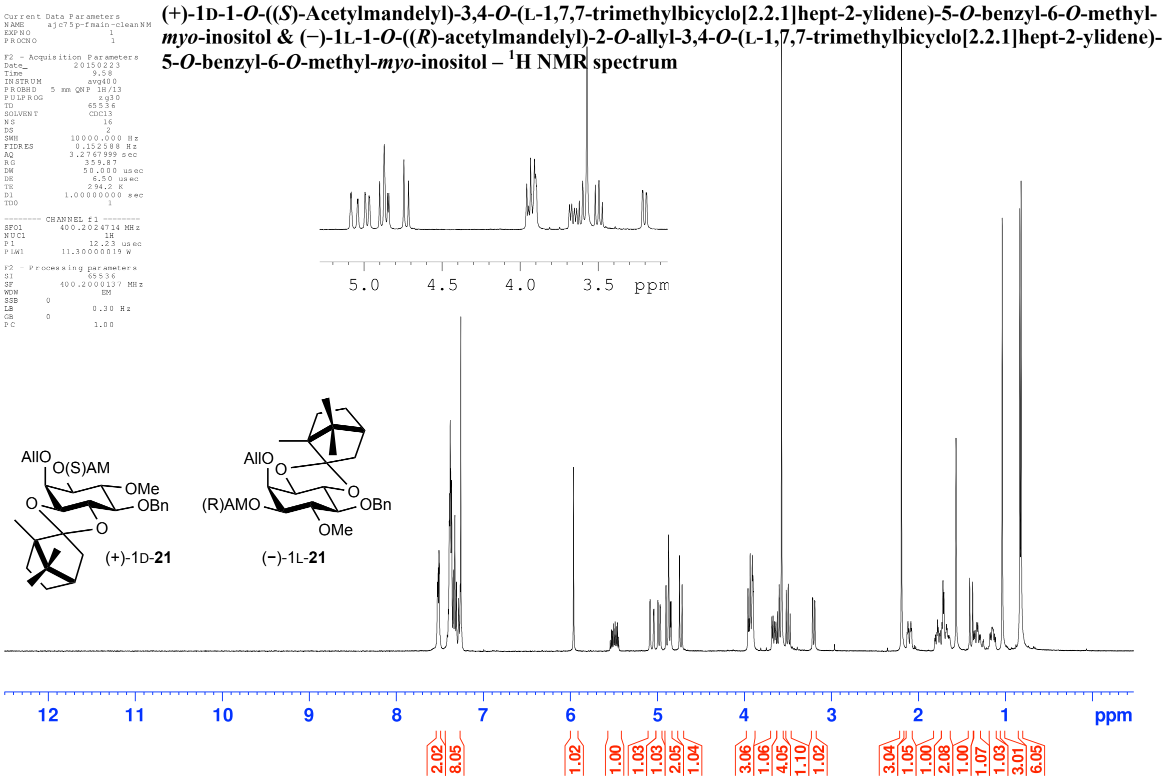

Supplementary Figure 35 – ^{1}H NMR (400 MHz; $CDCl_3$) of compounds (+)-1D-21 & (−)-1L-21

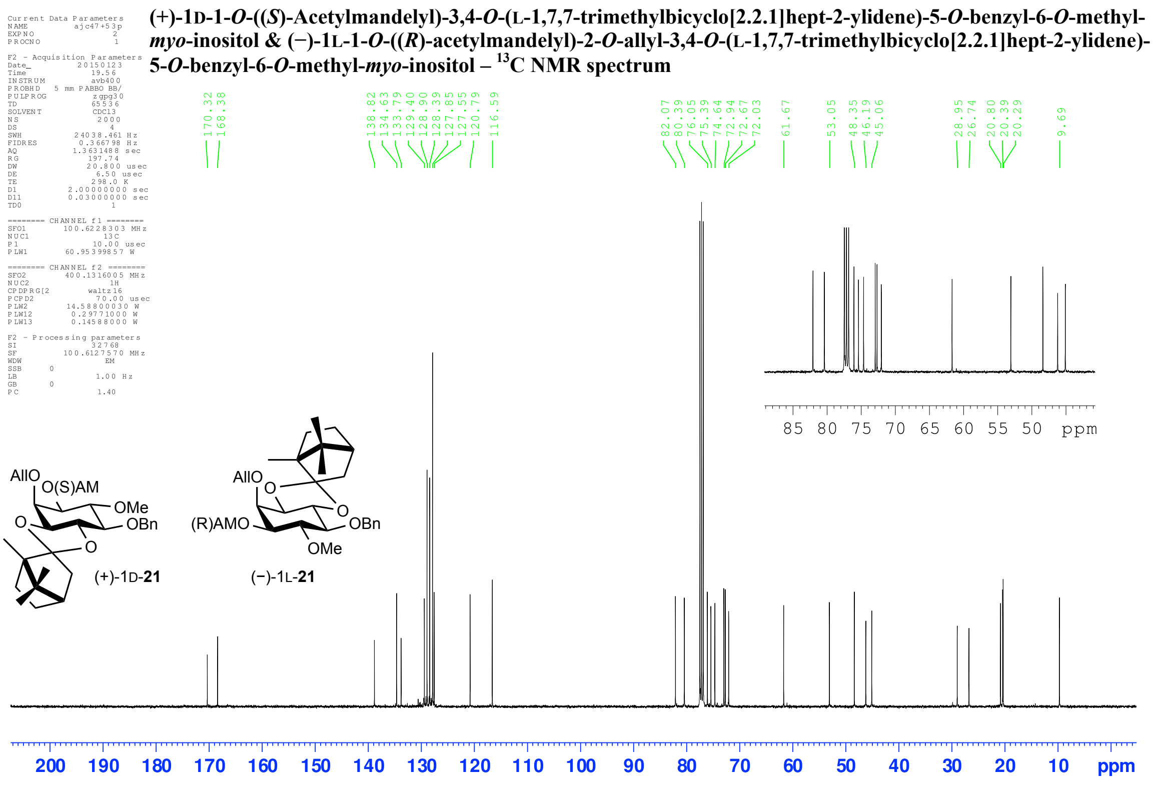

Supplementary Figure 36 – ^{13}C NMR (101 MHz; $CDCl_3$) of compounds (+)-1D-21 & (−)-1L-21

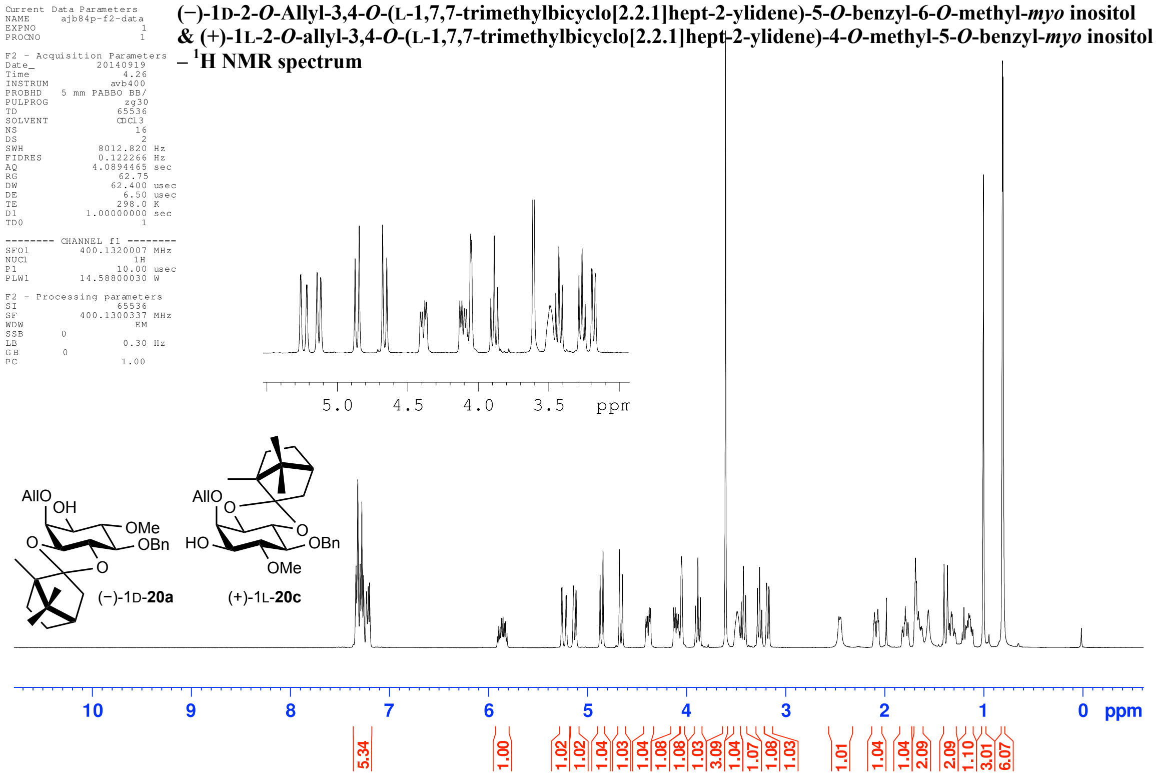

Supplementary Figure 37 – ^{1}H NMR (400 MHz; $CDCl_3$) of compounds (−)-1D-20a & (+)-1L-20c.

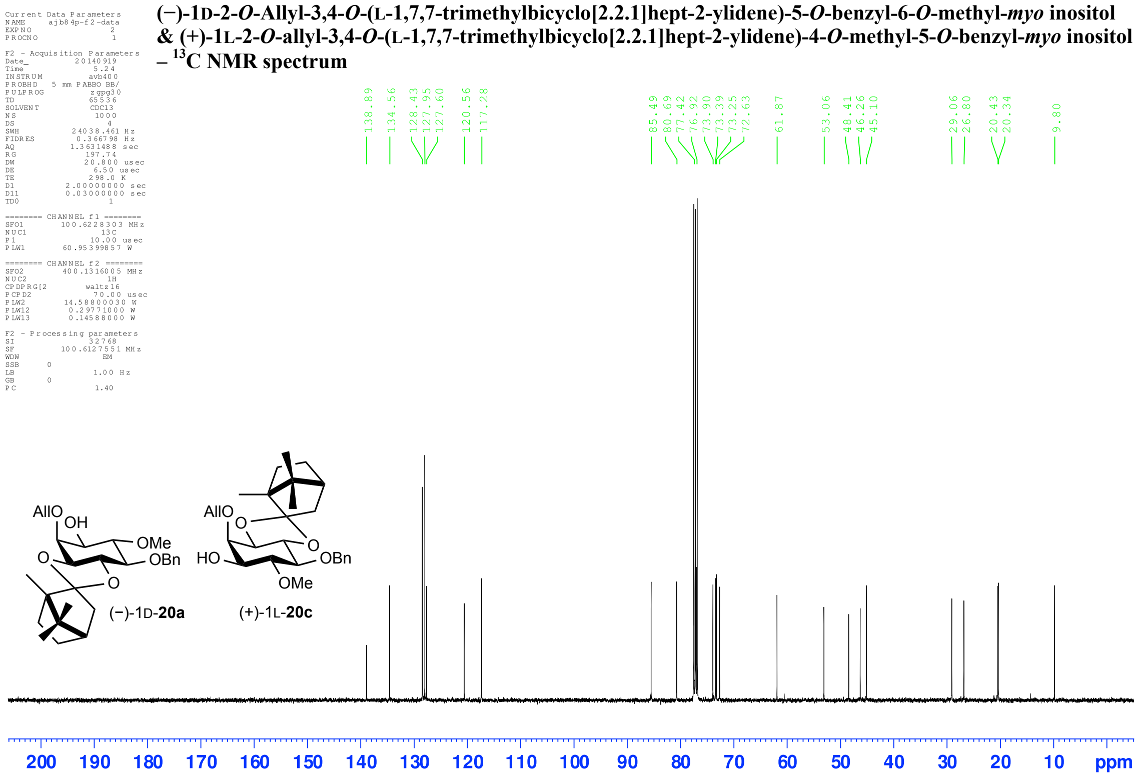

Supplementary Figure 38 – ^{13}C NMR (101 MHz; $CDCl_3$) of compounds (−)-1D-20a & (+)-1L-20c.

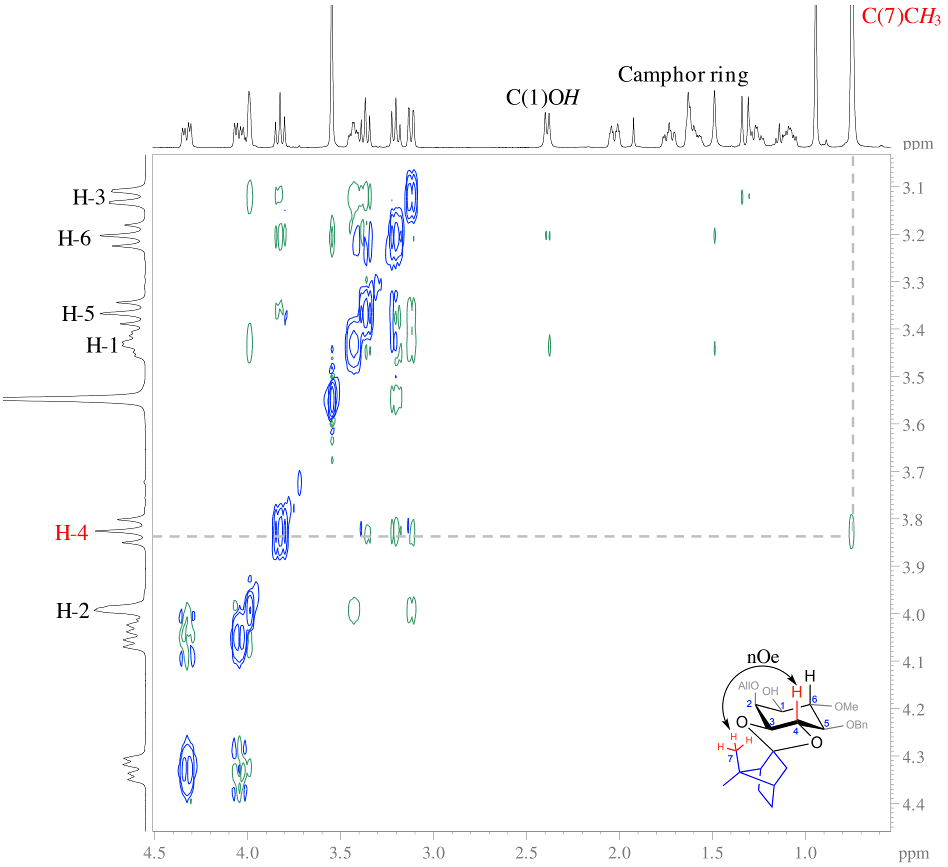

Supplementary Figure 39 – 2D Nuclear Overhauser Spectroscopy (NOESY) spectrum for compound (−)-1D-20a. The spectrum shows a through space correlation between the axial protein on the 4-position of the inositol ring and the proton on the camphor chiral auxiliary.

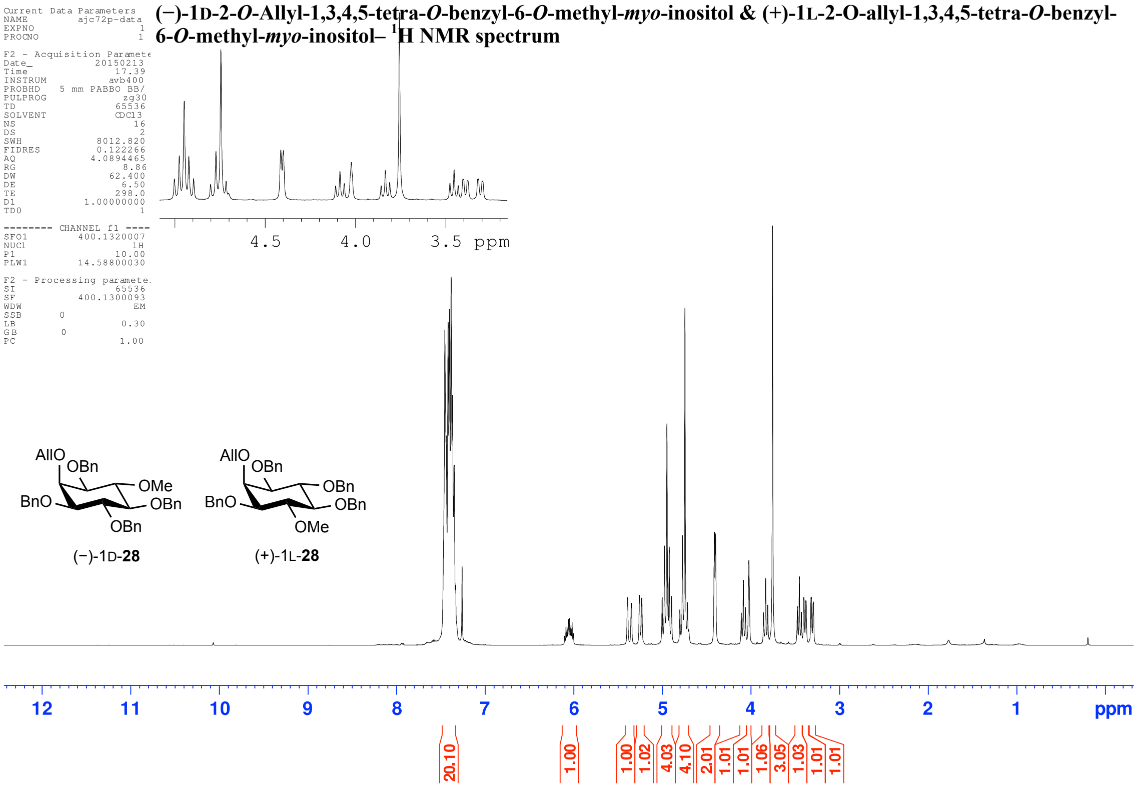

Supplementary Figure 40 – ^{1}H NMR (400 MHz; CDCl$_3$) of compounds (−)-1D-28 & (+)-1L-28

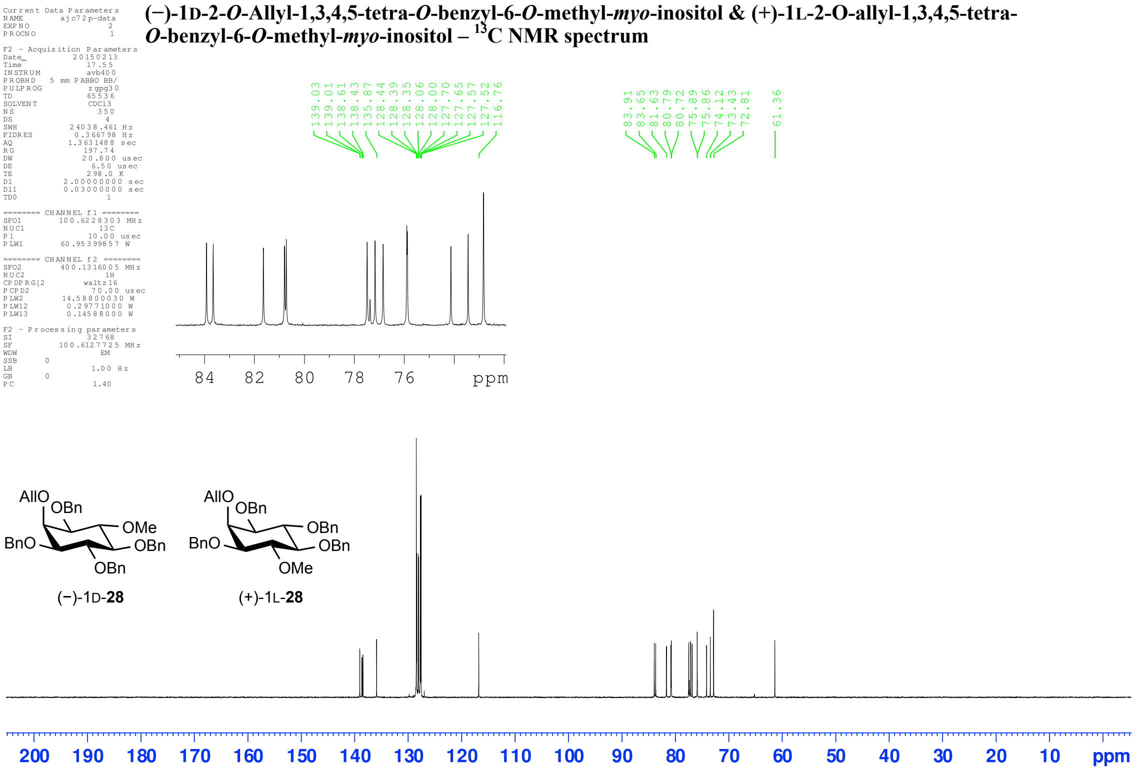

Supplementary Figure 41 – ^{13}C NMR (101 MHz; CDCl$_3$) of compounds (−)-1D-28 & (+)-1L-28

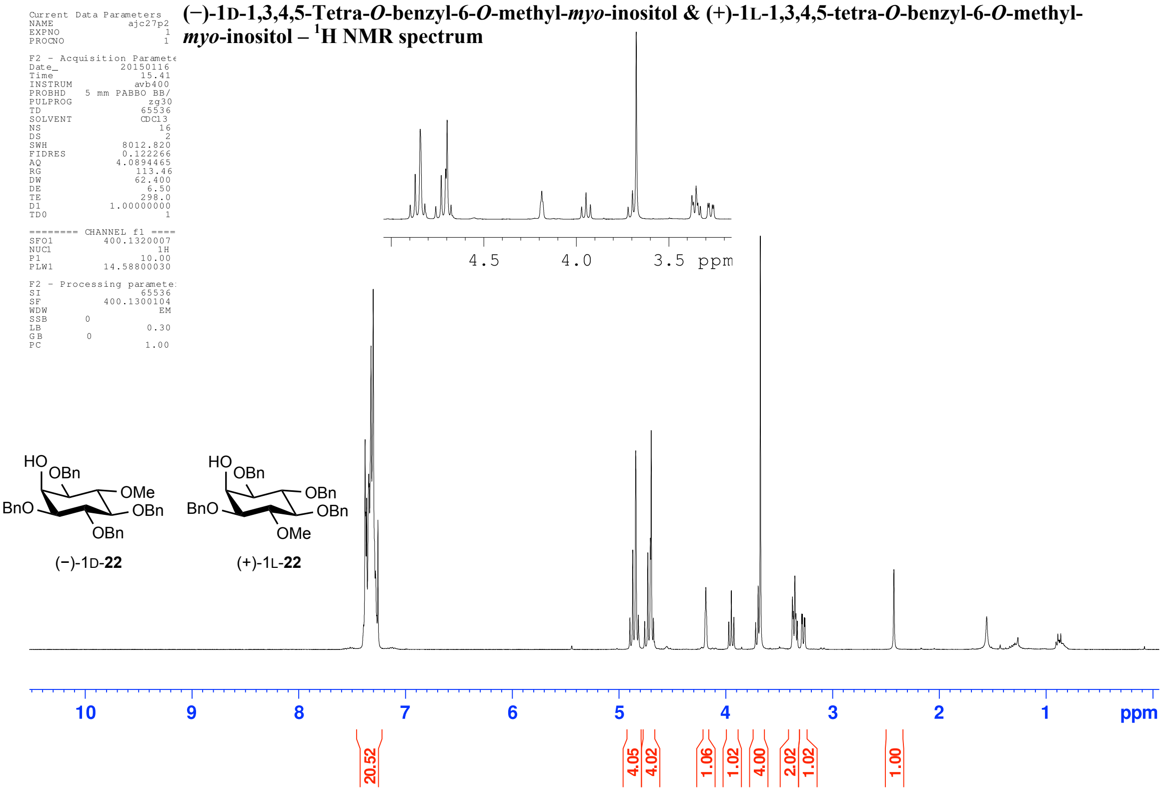

Supplementary Figure 42 – ¹H NMR (400 MHz; CDCl₃) of compounds (−)-1D-22 & (+)-1L-22

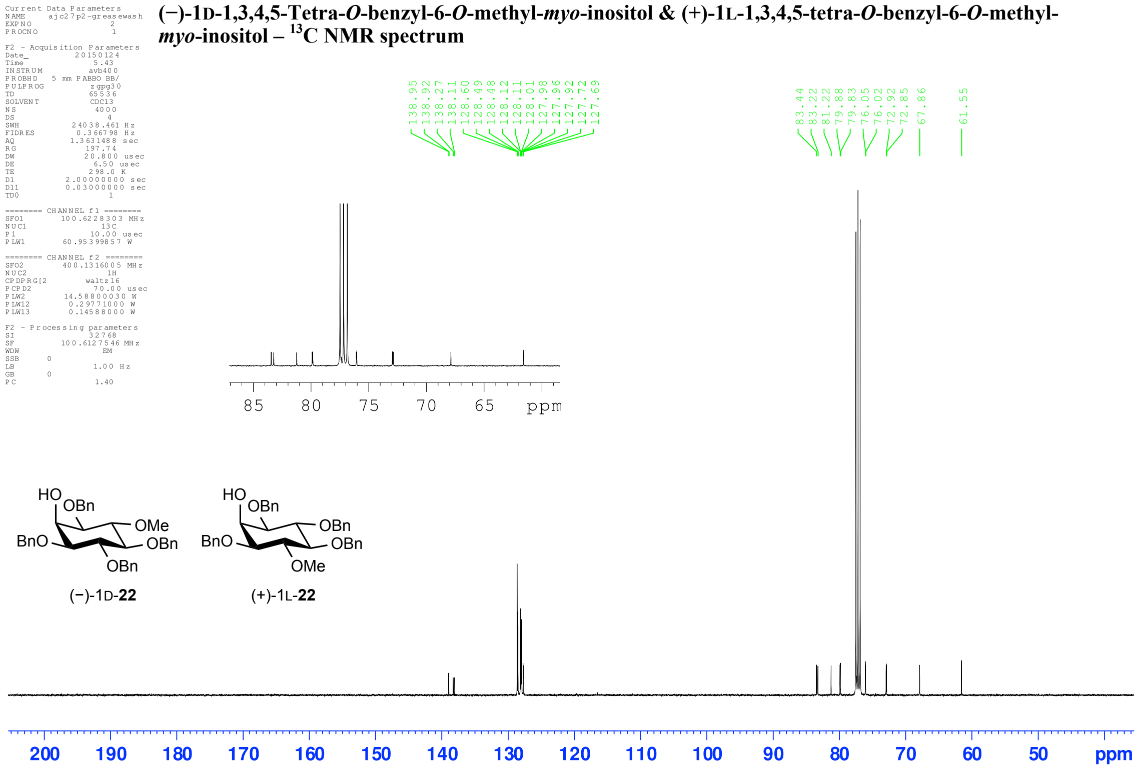

Supplementary Figure 43 – ¹³C NMR (101 MHz; CDCl₃) of compounds (−)-1D-22 & (+)-1L-22

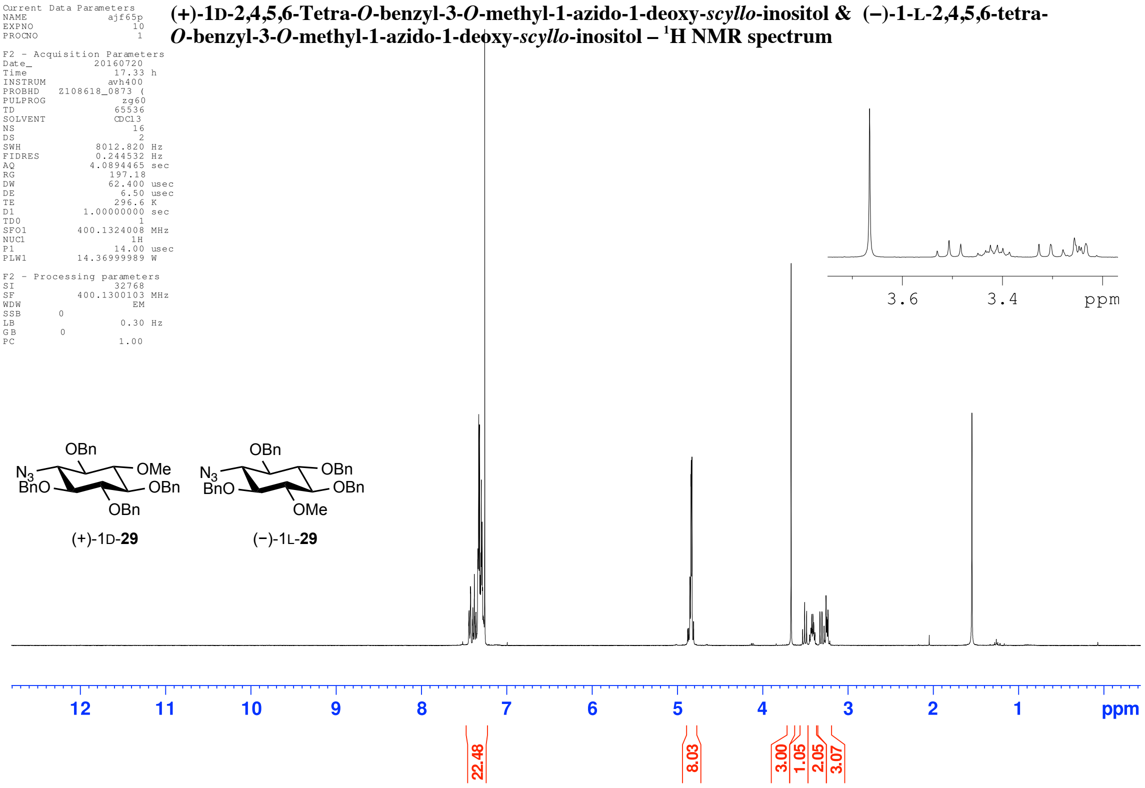

Supplementary Figure 44 – ^{1}H NMR (500 MHz; CDCl$_3$) of compounds (+)-1D-29 & (−)-1L-29

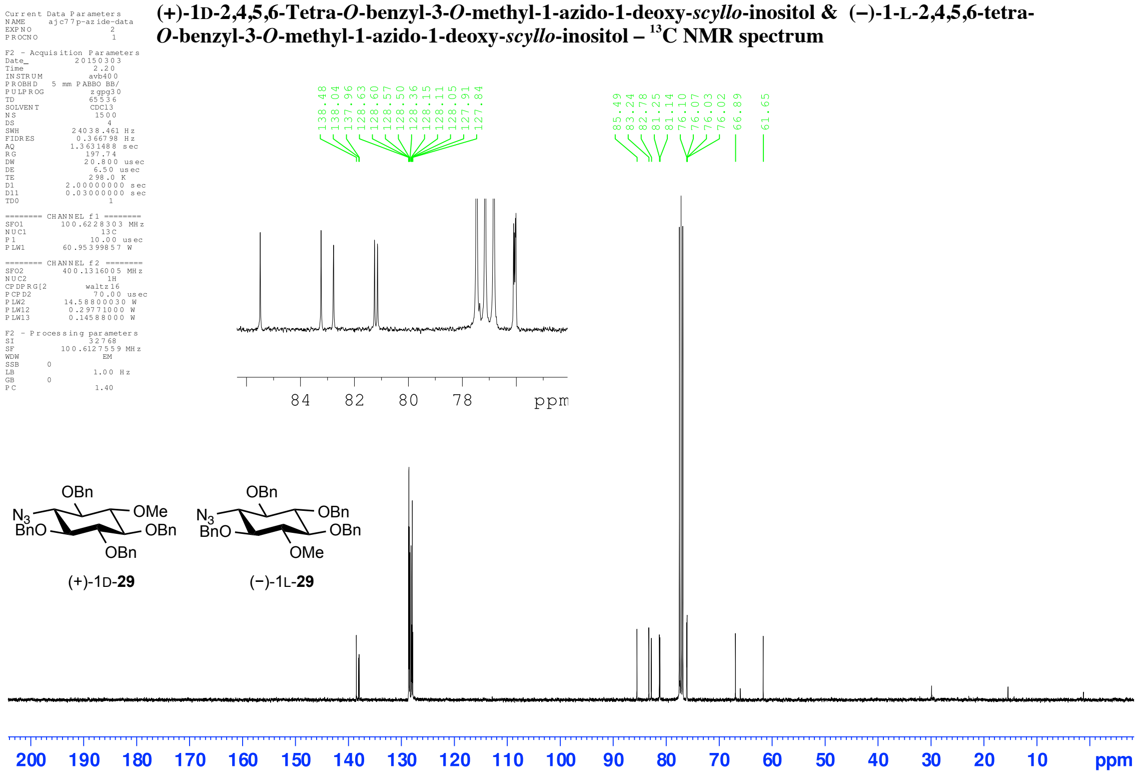

Supplementary Figure 45 – ^{13}C NMR (101 MHz; CDCl$_3$) of compounds (+)-1D-29 & (−)-1L-29

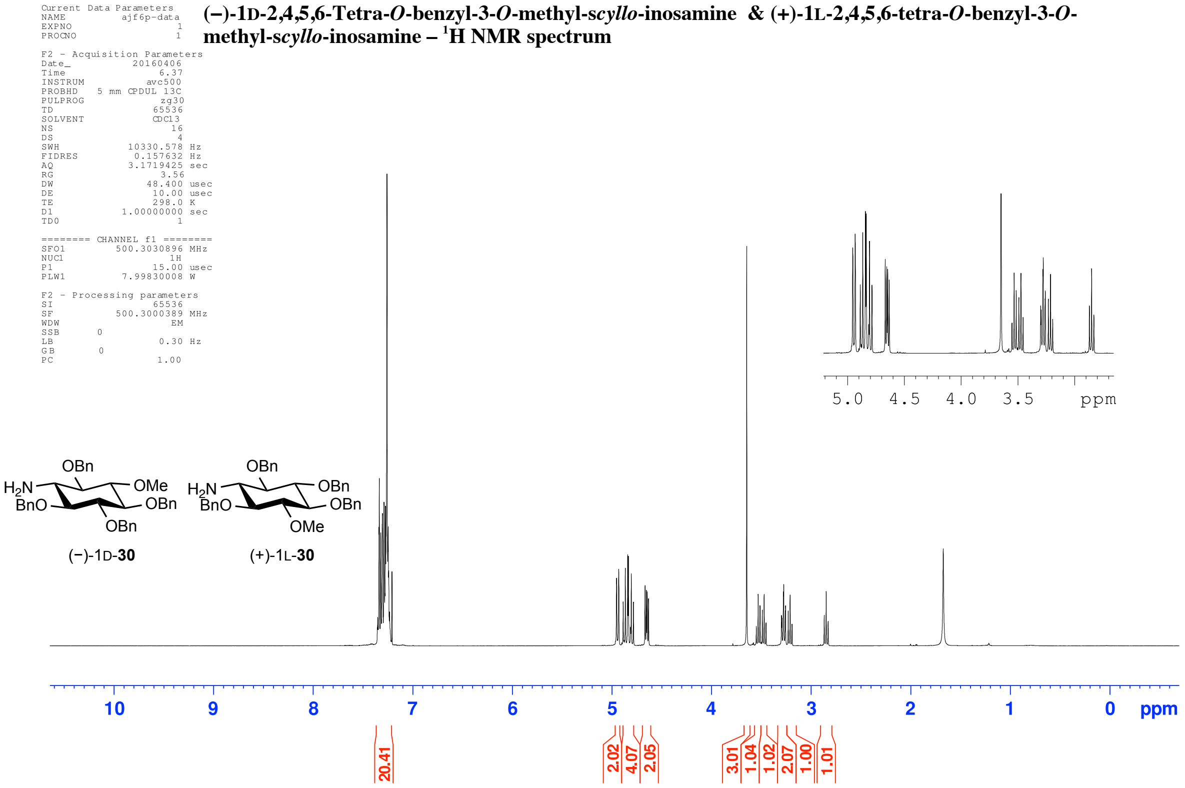

Supplementary Figure 46 – ¹H NMR (500 MHz; CDCl₃) of compounds (−)-1D-30 & (+)-1L-30

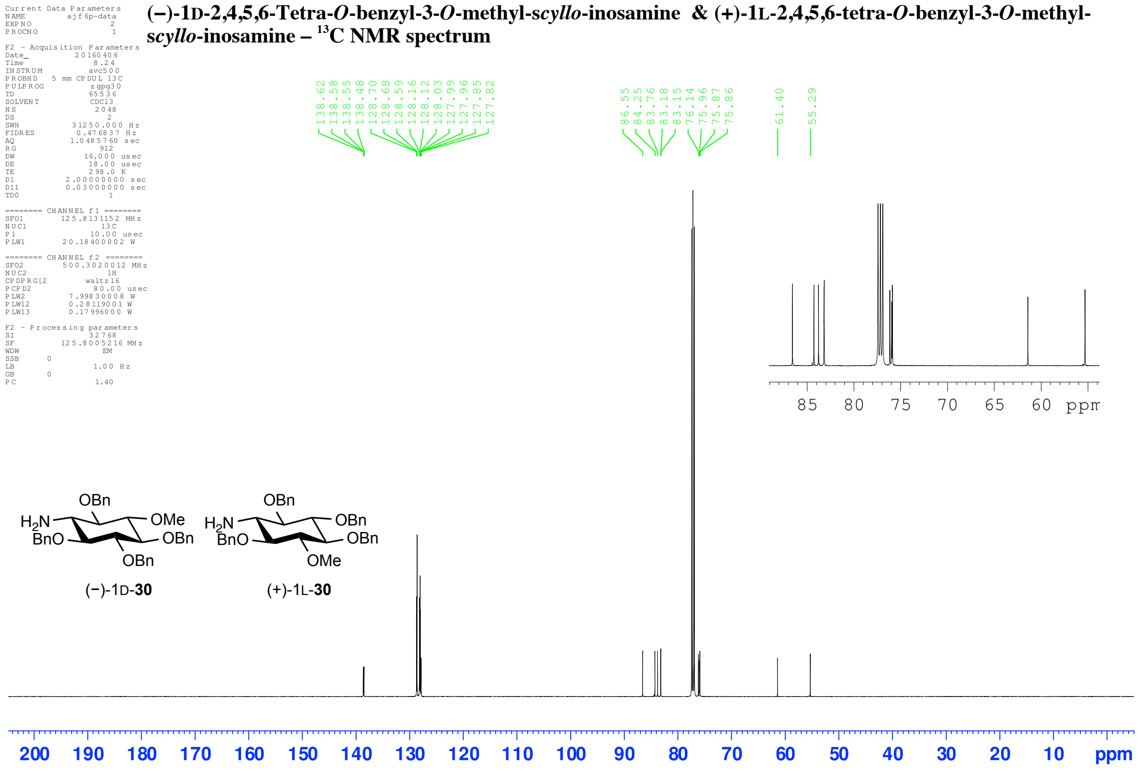

Supplementary Figure 47 – ¹³C NMR (126 MHz; CDCl₃) of compounds (−)-1D-30 & (+)-1L-30

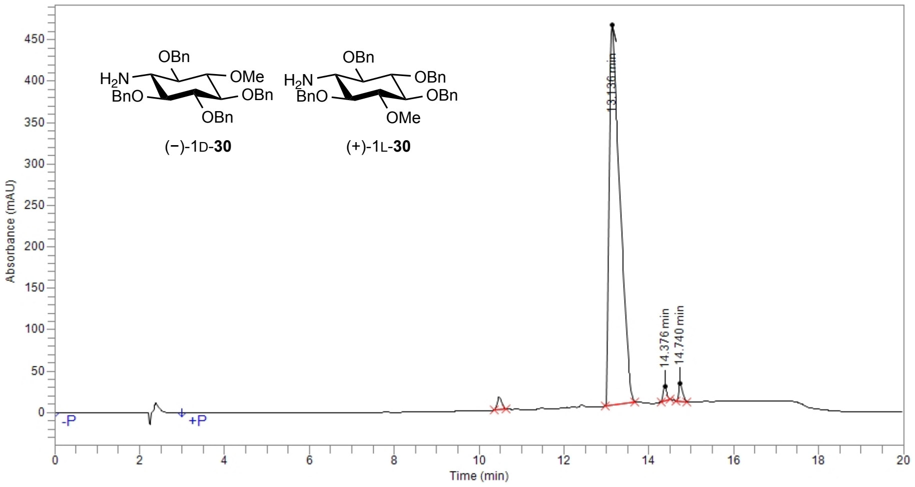

Supplementary Figure 48 – RP-HPLC trace of compounds (+)-1D-30 & (−)-1L-30. Method 1, t = 13.136 min, 95.81%

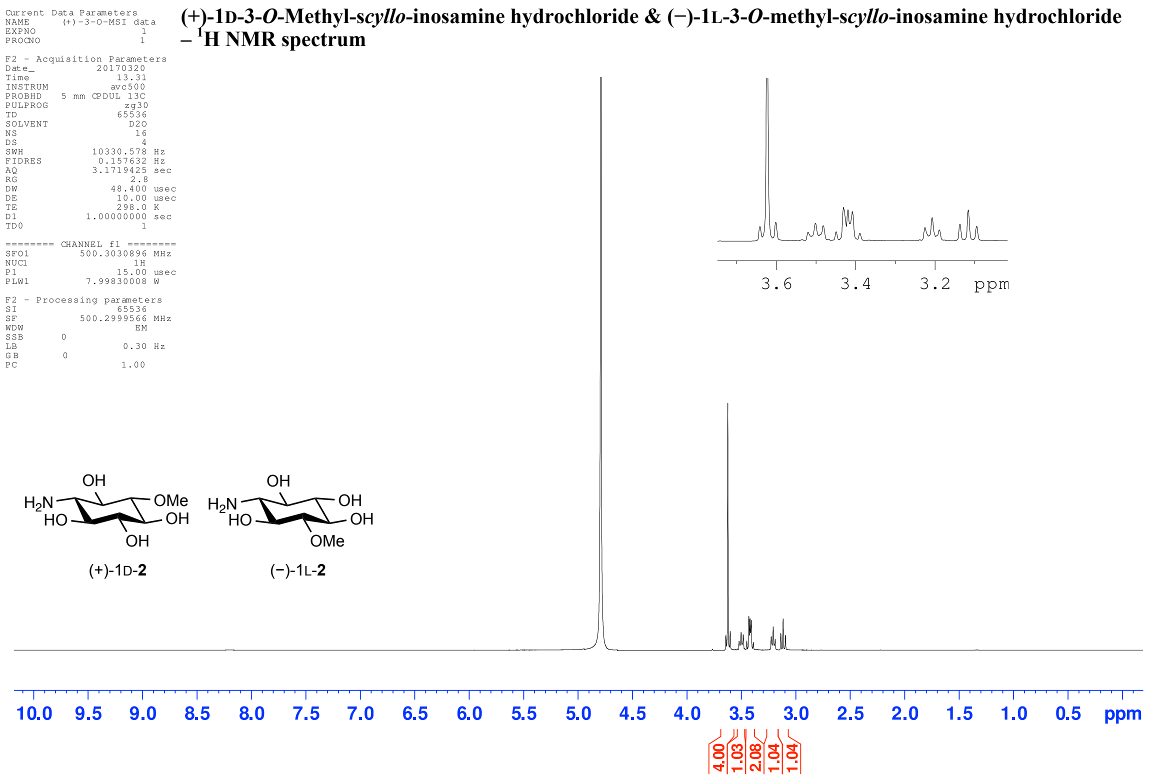

Supplementary Figure 49 – ¹H NMR (500 MHz; D_2O) of compounds (+)-1D-2 & (−)-1L-2

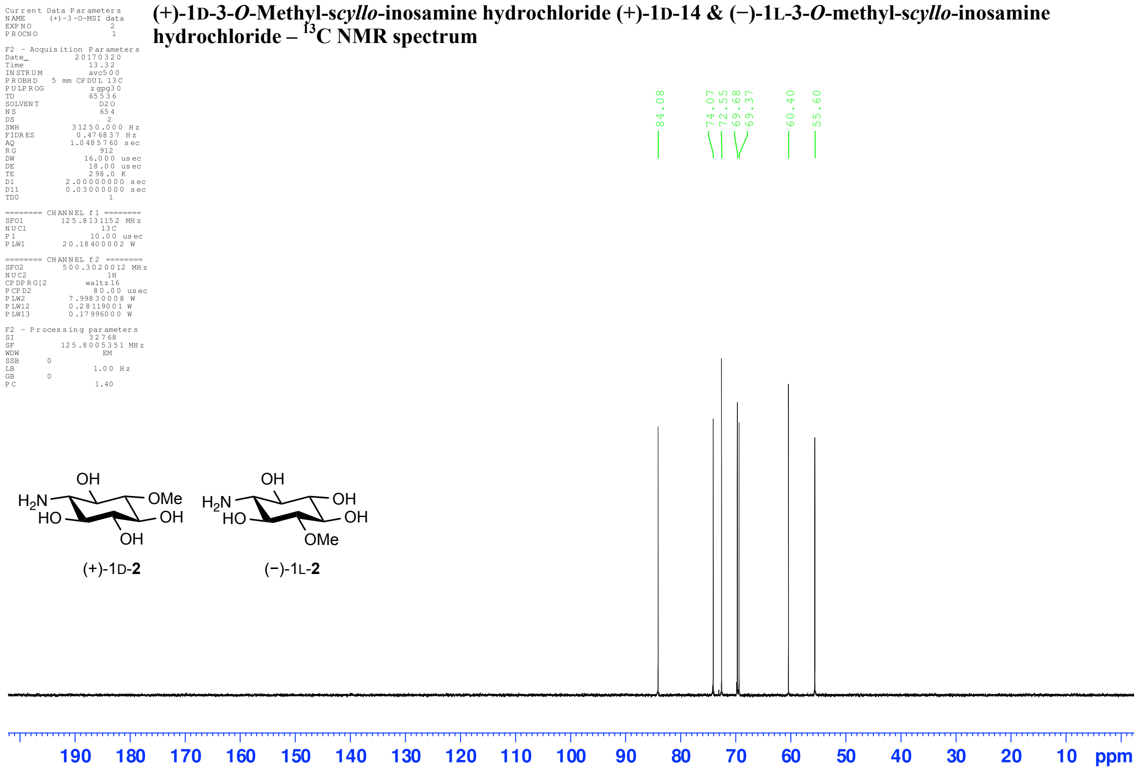

Supplementary Figure 50 – ^{13}C NMR (126 MHz; D_2O) of compounds (+)-1D-2 & (−)-1L-2

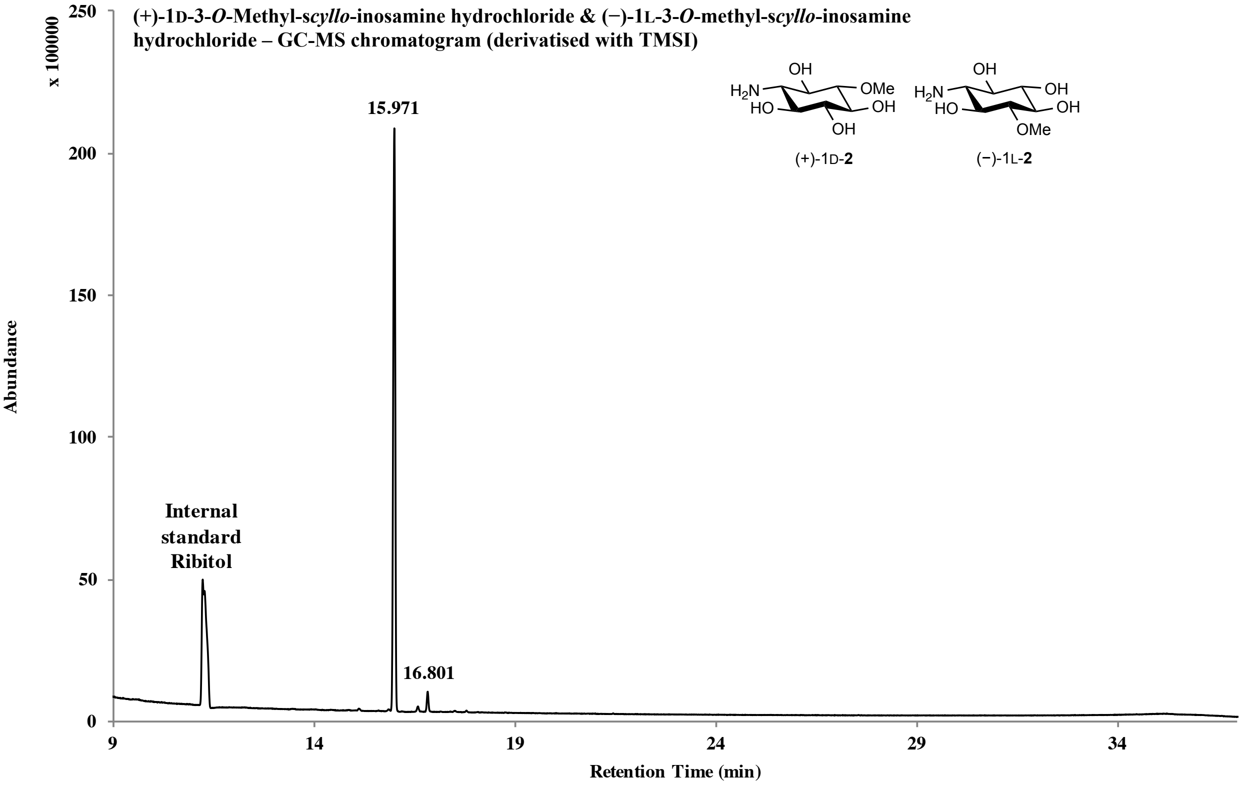

Supplementary Figure 51 – GS-MS chromatogram of compound 2. t = 15.971 min, 97.27%.

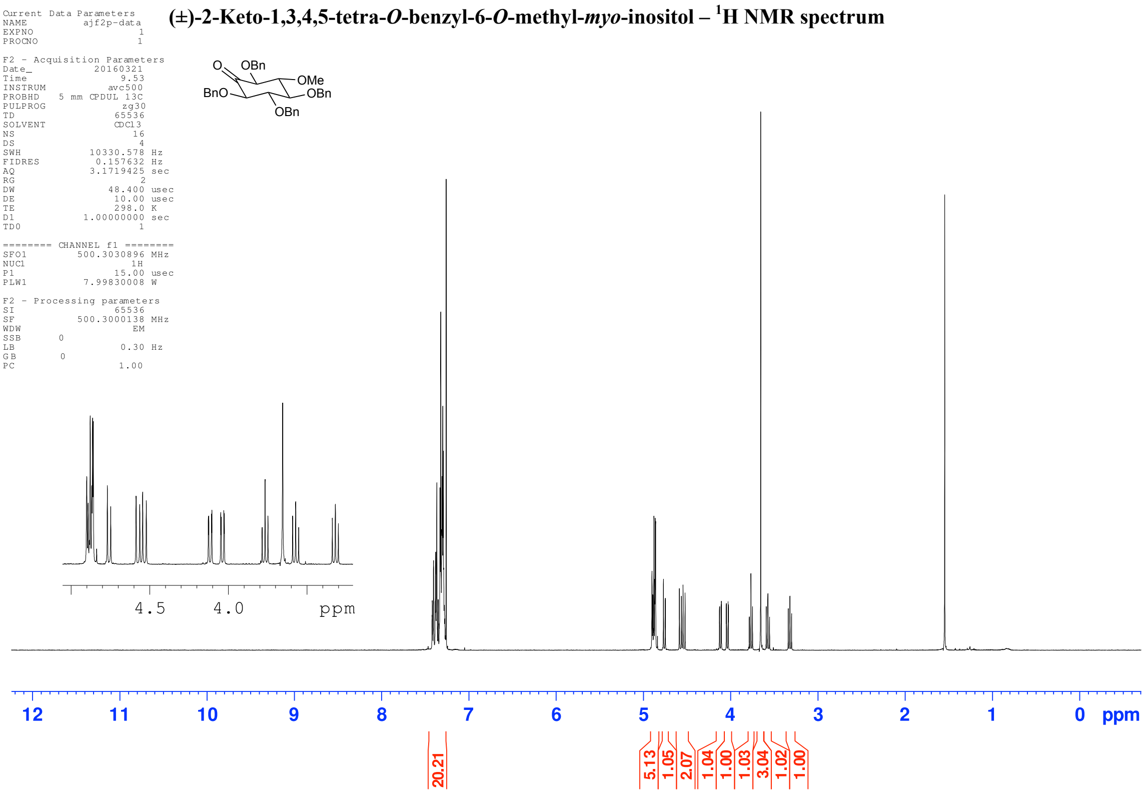

Supplementary Figure 52 – ^{1}H NMR (500 MHz; $CDCl_3$) of compounds (±)-23

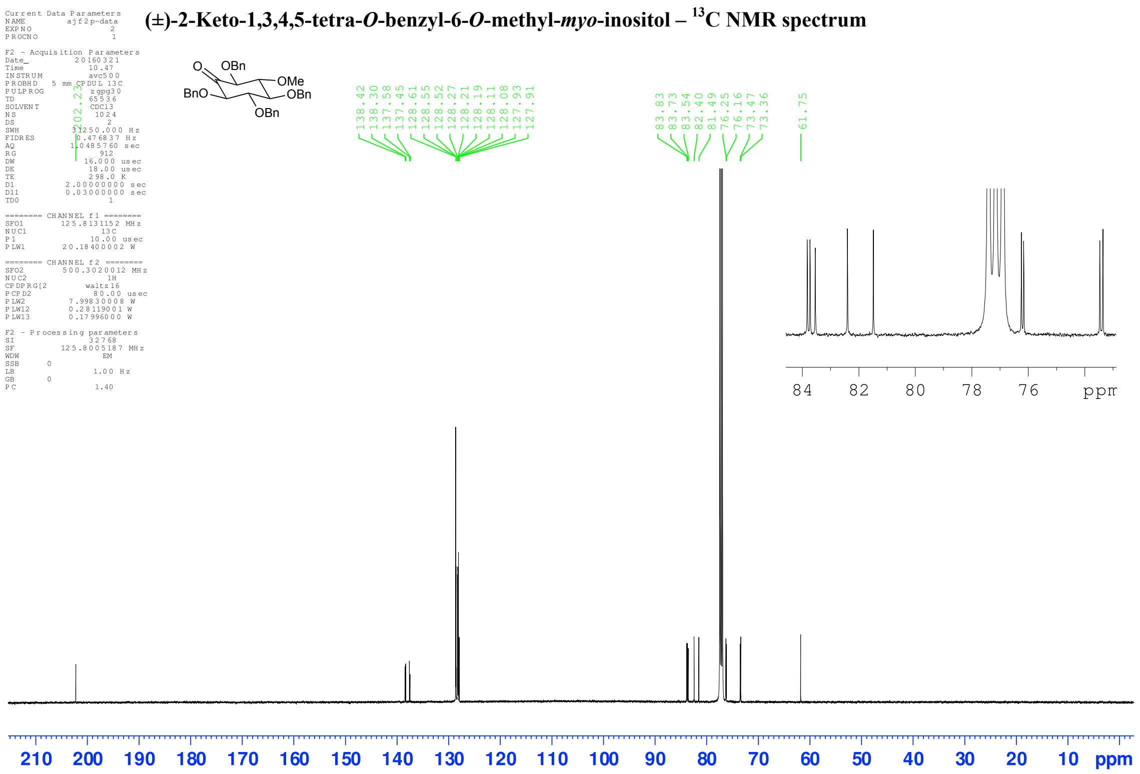

Supplementary Figure 53 – ^{13}C NMR (126 MHz; $CDCl_3$) of compounds (±)-23

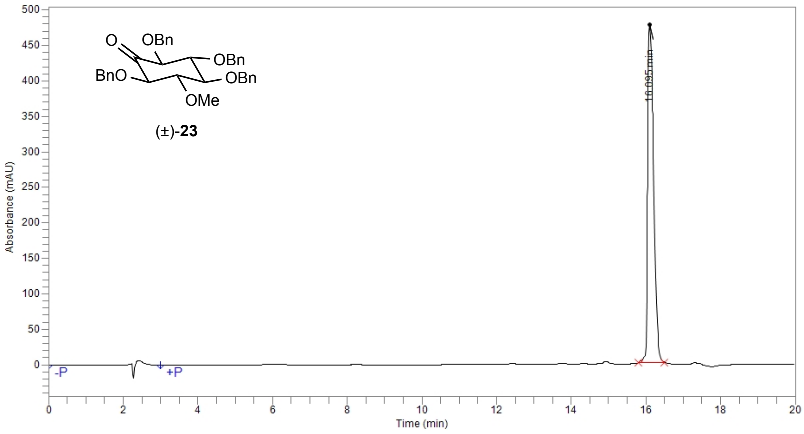

Supplementary Figure 54 – RP-HPLC trace of compound (±)-23. Method 1 – t = 16.095 min, 99.80%.

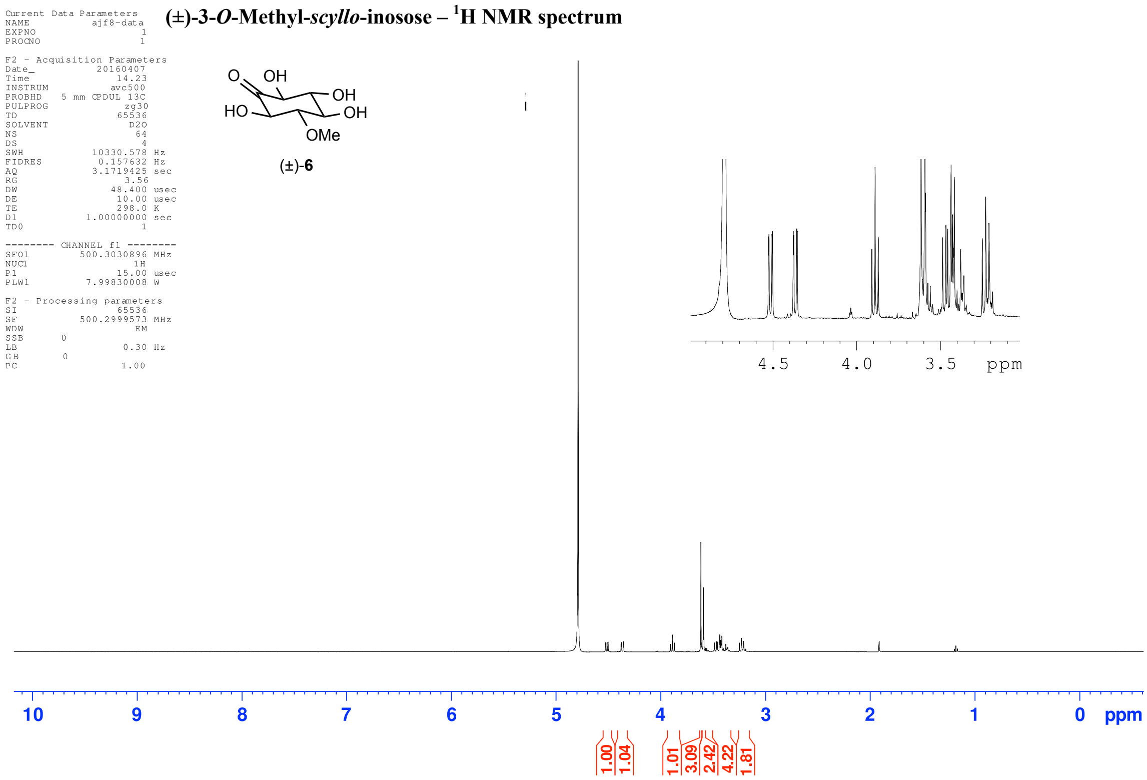

Supplementary Figure 55 – ¹H NMR (500 MHz; D_2O) of compounds (±)-6

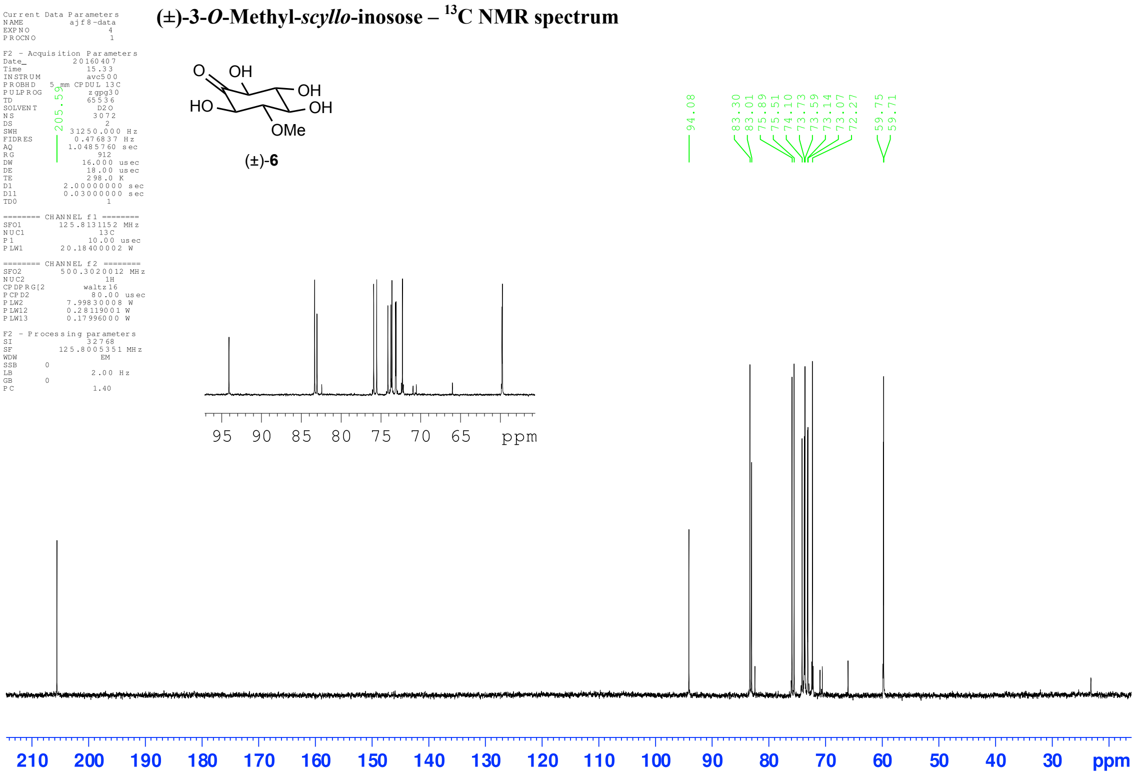

Supplementary Figure 56 – ^{13}C NMR (126 MHz; D_2O) of compounds (±)-6

Supplementary References

1. Fulmer, G. R. *et al.* NMR chemical shifts of trace impurities: common laboratory solvents, organics, and gases in deuterated solvents relevant to the organometallic chemist. *Organometallics* **29**, 2176–2179 (2010).

2. Armarego, W. L. E. & Chai, C. L. L. *Purification of Laboratory Chemicals*. (Butterworth-Heinemann, 2013).

3. Pangborn, A. B., Giardello, M. A., Grubbs, R. H., Rosen, R. K. & Timmers, F. J. Safe and convenient procedure for solvent purification. *Organometallics* **15**, 1518–1520 (1996).

4. Billington, D. C. *et al.* The total synthesis of *myo*-inositol phosphates via *myo*-inositol orthoformate. *J. Chem. Soc., Perkin Trans* **1**, 1423 (1989).

5. Lee, H. W. & Kishi, Y. Synthesis of mono-and unsymmetrical bis-orthoesters of *scyllo*-inositol. *J. Org. Chem.* **50**, 4402–4404 (1985).

6. Jagdhane, R. C. & Shashidhar, M. S. A formal synthesis of valiolamine from *myo*-inositol. *Tetrahedron* **67**, 7963–7970 (2011).

7. Billington, D. C. Recent developments in the synthesis of *myo*-inositol phosphates. *Chem. Soc. Rev.* **18**, 83–122 (1989).

8. Lauber, M. B., Daniliuc, C.-G. & Paradies, J. Desymmetrization of 4,6-diprotected *myo*-inositol. *Chem. Commun.* **49**, 7409–7411 (2013).

9. Mart, A. & Shashidhar, M. S. Elaboration of the ether cleaving ability and selectivity of the classical Pearlman's catalyst [$Pd(OH)_2/C$]: concise synthesis of a precursor for a myo-inositol pyrophosphate. *Tetrahedron* **68**, 9769–9776 (2012).

10. Florence, G., Aslam, T., Miller, G., Milne, G. & Conway, S. Thieme chemistry journal awardees - where are they Now? Synthesis of the marine glycolipid dioctadecanoyl discoside. *Synlett* **2009**, 3099–3102 (2009).

11. Koto, S. *et al.* Syntheses of penta-*O*-benzyl-*myo*-inositols, *O*-*β*-L-arabinosyl-(1→2)-*sn*-*myo*-

inositol, *O*-*α*-D-galactosyl-(1→3)-*sn*-*myo*-inositol, and *O*-*α*-D-galactosyl-(1→6)-*O*-*α*-D-galactosyl-(1→3)-*sn*-*myo*-inositol. *Bull. Chem. Soc. Jpn.* **73**, 2521–2529 (2000).

12. Nashed, M. A. & Anderson, L. Organotin derivatives and the selective acylation and alkylation of the equatorial hydroxy group in a vicinal, equatorial-axial pair. *Tetrahedron Lett.* **17**, 3503–3506 (1976).

13. Phenix, C. P., Nienaber, K., Tam, P. H., Delbaere, L. T. J. & Palmer, D. R. J. Structural, functional and calorimetric investigation of MosA, a dihydrodipicolinate synthase from *Sinorhizobium meliloti* L5–30, does not support involvement in rhizopine biosynthesis. *Chembiochem* **9**, 1591–1602 (2008).

14. Schoffers, E., Gurung, S. R., Kohler, P. R. A. & Rossbach, S. Chemical synthesis of *scyllo*-inosamine and catabolism studies in *Sinorhizobium meliloti*. *Bioorg. Med. Chem.* **16**, 7838–7842 (2008).

15. Palatinus, L. & Chapuis, G. SUPERFLIP–a computer program for the solution of crystal structures by charge flipping in arbitrary dimensions. *J. Appl. Crystallogr.* **40**, 786–790 (2007).

16. Betteridge, P. W., Carruthers, J. R., Prout, K. & Watkin, D. J. CRYSTALS version 12: software for guided crystal structure analysis. *J. Appl. Crystallogr.* **36**, 1487 (2003).

17. Thompson, A. L. & Watkin, D. J. CRYSTALS enhancements: absolute structure determination. *J. Appl. Crystallogr.* **44**, 1017–1022 (2011).

18. Post, G. G. & Anderson, L. Cyclitols and their methyl ethers. III. Catalytic air oxidation, the hydrogenolysis of inososes, and some pentol and tetrol methyl ethers 1-3. *J. Am. Chem. Soc.* **84**, 471–478 (1962).

19. Koncz, C. & Schell, J. The promoter of TL-DNA gene 5 controls the tissue-specific expression of chimaeric genes carried by a novel type of *Agrobacterium* binary vector. *MGG Mol. Gen. Genet.* **204**, 383–396 (1986).

20. Lazo, G. R., Stein, P. A. & Ludwig, R. A. A DNA transformation–competent *Arabidopsis* genomic library in *Agrobacterium*. *Nat. Biotechnol.* **9**, 963–967 (1991).

21. Stougaard, J., Abildsten, D. & Marcker, K. A. The *Agrobacterium rhizogenes* pRi TL-DNA segment as a gene vector system for transformation of plants. *MGG Mol. Gen. Genet.* **207**, 251–255 (1987).

22. Johnston, A. W. B. & Beringer, J. E. Identification of the Rhizobium strains in pea root nodules using genetic markers. *Microbiology* **87**, 343–350 (1975).

23. Rubio Sanz, L., Prieto Carbajo, R. I., Imperial Ródenas, J., Palacios Alberti, J. M. & Brito Lopez, M. B. Identification and functional characterization of *Rhizobium leguminosarum* bv. *viciae* genetic systems involved in nickel homeostasis. (2010).

24. Galardini, M. *et al.* Exploring the symbiotic pangenome of the nitrogen-fixing bacterium *Sinorhizobium meliloti*. *BMC Genomics* **12**, 1 (2011).

25. Galardini, M., Pini, F., Bazzicalupo, M., Biondi, E. G. & Mengoni, A. Replicon-dependent bacterial genome evolution: the case of *Sinorhizobium meliloti*. *Genome Biol. Evol.* **5**, 542–558 (2013).

26. Rosenberg, C., Boistard, P., Dénarié, J. & Casse-Delbart, F. Genes controlling early and late functions in symbiosis are located on a megaplasmid in *Rhizobium meliloti*. *Mol. Gen. Genet. MGG* **184**, 326–333 (1981).

27. Meade, H. M., Long, S. R., Ruvkun, G. B., Brown, S. E. & Ausubel, F. M. Physical and genetic characterization of symbiotic and auxotrophic mutants of *Rhizobium meliloti* induced by transposon Tn*5* mutagenesis. *J. Bacteriol.* **149**, 114–122 (1982).

28. Schneiker-Bekel, S. *et al.* The complete genome sequence of the dominant *Sinorhizobium meliloti* field isolate SM11 extends the *S. meliloti* pan-genome. *J. Biotechnol.* **155**, 20–33 (2011).

29. Shanks, R. M. Q., Kadouri, D. E., MacEachran, D. P. & O'Toole, G. A. New yeast

recombineering tools for bacteria. *Plasmid* **62**, 88–97 (2009).

30. Geddes, B. A., Mendoza-Suárez, M. A. & Poole, P. S. A bacterial expression vector archive (BEVA) for flexible modular assembly of Golden Gate-compatible vectors. *Front. Microbiol.* **9**, 3345 (2019).

31. Berrow, N. S., Alderton, D. & Owens, R. J. The precise engineering of expression vectors using high-throughput In-Fusion^{TM} PCR cloning. *High Throughput Protein Expr. Purif. Methods Protoc.* 75–90 (2009).

32. Frederix, M. *et al.* Mutation of *praR* in *Rhizobium leguminosarum* enhances root biofilms, improving nodulation competitiveness by increased expression of attachment proteins. *Mol. Microbiol.* **93**, 464–478 (2014).

33. Ferguson, G. P. *et al.* Similarity to peroxisomal-membrane protein family reveals that Sinorhizobium and Brucella BacA affect lipid-A fatty acids. *Proc Natl Acad Sci USA* **101**, 5012–5017 (2004).

34. Choi, K.-H. *et al.* Genetic tools for select-agent-compliant manipulation of *Burkholderia pseudomallei*. *Appl. Environ. Microbiol.* **74**, 1064–75 (2008).

35. Choi, K.-H. *et al.* A Tn*7*-based broad-range bacterial cloning and expression system. *Nat. Methods* **2**, 443–448 (2005).

36. Goodman, A. L. *et al.* Identifying genetic determinants needed to establish a human gut symbiont in its habitat. *Cell Host Microbe* **6**, 279–89 (2009).

37. Weber, E., Engler, C., Gruetzner, R., Werner, S. & Marillonnet, S. A modular cloning system for standardized assembly of multigene constructs. *PLoS One* **6**, e16765 (2011).

38. Trost, B. M. *et al.* On the use of the *O*-methylmandelate ester for establishment of absolute configuration of secondary alcohols. *J. Org. Chem.* **51**, 2370–2374 (1986).

39. Latypov, S. K., Seco, J. M., Quinoa, E. & Riguera, R. Conformational structure and dynamics of arylmethoxyacetates: DNMR spectroscopy and aromatic shielding effect. *J. Org. Chem.* **60**,

504–515 (1995).

40. Seco, J. M., Latypov, S. K., Quiñoá, E. & Riguera, R. Determining factors in the assignment of the absolute configuration of alcohols by NMR. The use of anisotropic effects on remote positions. *Tetrahedron* **53**, 8541–8564 (1997).

41. Poole, P. S., Blyth, A., Reid, C. J. & Walters, K. *myo*-Inositol catabolism and catabolite regulation in *Rhizobium leguminosarum* bv. *viciae*. *Microbiology* **140**, 2787–2795 (1994).
