# Supplementary material for: Engineering transkingdom signalling in plants to control gene expression in rhizosphere bacteria
Source: Nat Commun. 2019 Jul 31;10:3430. doi: 10.1038/s41467-019-10882-x (PMC6668481; doi:10.1038/s41467-019-10882-x)
Supplement: Supplementary file 4 — Description of Additional Supplementary Files [file 41467_2019_10882_MOESM4_ESM.docx]

**Description of Additional Supplementary Files**

File Name: **Supplementary Data 1**
Description: Quantification of rhizopine production in Medicago hairy roots

EIC (m/z 245) chromatograms of *Medicago truncatula* transgenic hairy root samples (N = 3) used to determine the peak area of rhizopine and quantify its production.

File Name: **Supplementary Data 2**
Description: Quantification of rhizopine production in Barley T0 roots

EIC (m/z 245) chromatograms of transgenic barley T0 plant root samples (n = 10) used to determine the peak area of rhizopine and quantify its production.

File Name: **Supplementary Data 3**
Description: Quantification of rhizopine production in Barley T1 roots Experiment 1

EIC (m/z 245) chromatograms of transgenic barley T1 plant root samples (Expt1, n = 10) used to determine the peak area of rhizopine and quantify its production.

File Name: **Supplementary Data 4**
Description: Quantification of rhizopine production in Barley T1 roots Experiment 2

EIC (m/z 245) chromatograms of transgenic barley T1 plant root samples (Expt2, n = 10) used to determine the peak area of rhizopine and quantify its production.
