# Supplementary material for: Engineering transkingdom signalling in plants to control gene expression in rhizosphere bacteria
Source: Nat Commun. 2019 Jul 31;10:3430. doi: 10.1038/s41467-019-10882-x (PMC6668481; doi:10.1038/s41467-019-10882-x)
Supplement: Supplementary file 2 — Peer Review [file 41467_2019_10882_MOESM2_ESM.pdf]

Reviewers' comments:

Reviewer #1 (Remarks to the Author):

In some Rhizobium-legume symbioses, compounds known as rhizopines are synthesized by bacteroids in nodules and are subsequently catabolized by free-living cells of the producing strain. It has been proposed long ago that plant-produced rhizopines, and Agrobacterium opines as well, may be used to promote the competitiveness of an opine-catabolizing strain in the rhizosphere.
Along the same line, Geddes and co-workers propose to use a plant-produced rhizopine to control gene expression in rhizospheric bacteria, for the delivery of new function to plants such as, for example, biological nitrogen fixation. In this long-term perspective, the authors have engineered the synthesis of a specific rhizopine (scyllo-inosamine) in hairy roots of Medicago sativa and primary transformants of barley and shown that the rhizopine is secreted in the rhizosphere, as assessed with a rhizopine-biosensor strain.

General comments:
1. Novelty:
The use of a rhizopine to control bacterial gene expression in the rhizophere is a novel extension of the opine concept. Here the authors show that a rhizopine synthesized in engineered plants is secreted into the rhizosphere. Whether this will indeed allow controlling bacterial gene expression for the benefit of the plant remains however to be demonstrated.
The transkingdom nature of the signalling is novel for a rhizopine. Yet this mimicks the situation in Agrobacterium in which bacterial opine-synthesizing genes are naturally transferred to the host cells.
A novel synthetic pathway for the production of the rhizopine scyllo-inosamine was engineered in plants, although the stability of the transgenes was not assessed at this stage.
As another piece of novelty, the rhizopine biosynthetic pathway was clarified and the role of new genes (mosDEF) elucidated in Sinorhizobium meliloti but this is not central to the paper.

2. Evidence for conclusions:
Better –and more- plant images (Fig 1b, Fig3cd) and additional controls are needed to strenghten the conclusions (see specific comments below).
The amount of rhizopine compounds produced by the plants and the bacterial bioluminescence should be quantified and variability assessed. It was not clear to me whether the level of rhizopine production as detected by GCMS in nodules or barley roots was compatible with the rhizopine concentration needed to activate the fusion.

3. Significance:
The main significance of the work is in a long-term biotech perspective.

Specific comments:
4. l. 67-76. Evidence that the rhizopine is secreted from nodules of M. sativa needs additional controls. Was bioluminescence only observed at the nodule surface? Could the pOPS0046 reporter plasmid be transferred between Rlv3841 and S. meliloti L5-30 in the rhizosphere? Has a non-nodulating mutant of S. meliloti L5-30 been used as a negative control?
5. The picture in Figure 1b should be improved (nodules are barely visible). Only 3 plants have been tested. Do all nodules respond the same?
6. Fig1bc: how does the bioluminescence in situ (Fig 1b) compare with the bioluminescence in fig 1c?
7. l. 77- Fig 1c: Was scyllo-inosose 7 (SO) tested for signaling activity? (see below)
8. Fig1d: rhizopines are best active on the reporter fusion in the 0.1 to 1mM range. At high concentrations, the molecule can be both a signal and a substrate. Could the reporter Rlv3841 strain use rhizopine for growth in these conditions, with an impact on the global bioluminescence?
9. Fig2: There is little difference in the spectra generated by the IDH and IDH-MosB constructs. Similarly, little SIA1 seems to be produced in Medicago hairy roots (Fig 3a) or transgenic barley roots (Fig 3b). Therefore to which extent plants produce SIA1 is not clear to me. Alternatively, could

bioluminescence be due to secreted SO?
10. Fig3ab: how many roots have been assayed? Which portion of the roots (eg in barley) has been extracted?
11. Fig3cd: In addition to more detailed pictures, some quantification of plant-associated variation is needed. Maximum bioluminescence seems to be restricted to a very small part of the root system in barley (yellow spot). Was the reason for this investigated?
12. l 400. P. sativum plants are mentioned but corresponding data are not shown.

Reviewer #2 (Remarks to the Author):

This ms describes incorporation of the rhizopine <i>scyllo<i>-inosamine biosynthesis pathway into <i>M. truncatula</i> and barley, and detection of the signal molecule in the rhizosphere with a novel biosensor. This paper packs a lot of discovery into a small package. The authors have demonstrated the function of MosDEF and MosB, and the use of the MocB and MocR into a lux reporter vector to allow luminescent detection by bacteria in the rhizosphere. I recommend its publication, after attention is paid to some details below,

Small complaints:

While the work on mosDEF and MosB is novel, the use of IolG is not. There is a great deal of literature on the structure and function of B. subtilis IolG in particular, the enzyme the authors chose to work with, but this recent (post-1979) work is not cited. I don't want to encourage over-referencing, but this seems odd, and misleading to the reader. The authors should also discuss if they considered working instead with MocA, which is predicted to be an NAD-dependent dehydrogenase very similar to IolG (and if not, why not).

Line 87 states the "clear preference of the biosensor for [L over D] indicates that [L] is the naturally active enantiomer, at least in this context". I think this overstates what the data indicates. I would rather see a statement like "1L-2 has previously been proposed to be the naturally occurring enantiomer, and the preference for this enantiomer observed in figure 1 supports this."

Figure 2: dehydrogenase reactions in parts d and g are incorrect. (<i>Not</i> FAD + 2 H+, NAD + H+)

Does reference 28 have an error?

In the file of Supporting Information I received, the structure of compound 11 is floating in the wrong place (line 186). It should be on line 193. There also looks to be some problem with the structure on lone 297, forcing a page break. See also line 304. It's a general problem. Maybe in-line-with-text would work better?

Reviewer #3 (Remarks to the Author):

Important note: in this report, I used my own abbreviations to describe the various compounds to which this study refers.

The paper by Geddes and co-workers describes the possible engineering of trans-kingdom signaling between plants and bacteria. There are indeed several interests in such engineering though I estimate that the road is still long to reach durable and reproducible agricultural applications.

The authors investigated the metabolism of scyllo-inosamine (SI) and 3-O-methyl-scyllo-inosamine (3MSI), two compounds known as a rhizopines. These molecules are found in nitrogen fixing nodules incited by a few rhizobia on a limited number of legume species. They are synthesized and degraded by the rhizobia, possibly as a way to attract "compatible" rhizobia to the plant roots.

The first reported experiment consisted in cloning the promotor of a gene involved in rhizopine production fused to a lux reporter gene in a Rhizobium leguminosarum background (the reporter). When co-inoculated with Sinorhizobium meliloti strain L5-30 on Medicago sativa, the reporter strain "sensed" the production of rhizopine in nodules incited by strain L5-30 but not in nodules incited by a mutant of L-30 unable to produce rhizopines. The results are convincing but the reasoning for the experiment is not clear. A priori, rhizopine detection should rather involve (promotor of) catabolic rather than anabolic genes… A few words to explain the rationale would therefore be beneficial.

The specificity of the reporter system was investigated next by exposing it to various molecules such as SI and 3MSI, and related compounds. Using synthetic and commercially available compounds, the authors convincingly demonstrated that the reporter is indeed specific for SI and 3MSI. The remarkable result here is that the reporter preferentially detects one of the two enantiomers of 3MSI, which is also the only enantiomer that promotes the growth of strain L5-30.

The next reported data dealt with the biosynthetic route of 3MSI and the related genetic determinants. By comparing sequences of rhizobial genomes, the authors noted that: (i), the mosA gene is not widely distributed amongst the sequences; (ii), the mosBCDEF genes are present in all strains synthesizing 3MSI; and (iii), the mosDEF genes could encode a membrane-associated dehydrogenase complex. The suggested involvement of mosDEF in 3MSI synthesis (rather than degradation) was demonstrated using a mosE mutant that does not produce rhizopines. Though I globally agree with the result and proposed pathway, I found the presentation of the data quite complex. One of the reasons is the lack of consistency of abbreviations through figures and text. For example ononitol appears sometimes as D-ononitol, (+)-1D-4, or OL! Regarding the experiment with the mutants, I noted the accumulation of an identical compound in the extracts obtained with either mosB and mosE (figure 2a). The authors indicate in the text (lines 122/127) that the compound should be the same as the one accumulated by a strain that does not produce 3MSI in which mosDEF genes have been transferred. I disagree with this claim considering that the retention time of the compound in the mutants is 15.4 min while it is 15.6 min in the later strain. My feeling - and this needs to be confirmed and shown in figure 2a - is that the peak at 15.6 is ononitol (OL) as stated in figure 2b, indeed. OL appeared in Fig 2a for both mosB and mosE just because the intermediate compound 3-O-methyl-scyllo-inosose (3MIO) is unstable and converted back to OL spontaneously. In agreement with my opinion, mosB should accumulate 3MIO while mosE should accumulate OL, unless 3MIO spontaneously yield OL. This feeling is confirmed by the observation of the 3MIO standard appears to contain large amounts of OL. In my opinion, the authors should clarify these points.

To evaluate the possibility to generate 3MSI-producing plants, the authors attempted to express transiently mosDEF and mosB in Nicotiana leaves. No production of 3-MSI was observed. To circumvent this bottleneck, the authors though to engineer SI (and not 3MSI) production in plants. They proposed a pathway that originates from myo-inositol, and involved the synthesis of scyllo-inosose (IO, the non methylated version of 3MIO) from this polyol, using the enzyme inositol dehydrogenase IolG from B. subtilis. The authors combined purified IolG and MosB enzymes and demonstrated the production of SI in vitro. I have one comment regarding this result. In figure 2e, I would have expected that the incubation of IolG would generate IO from myo-inositol. This is not the case. There is no comment on this issue in the text and I found that disturbing. A line to explain the result would be beneficial. Furthermore, the authors indicated that they expected this pathway to work into plants but did not report the results obtained with the two above enzymes. I suspect that this enzyme couple did not allow the production of SI in plants, possibly due to GC% or codon usage characteristics of the encoding genes. Here also a comment would be welcome.

The authors next used another inositol dehydrogenase, namely IdhA from R. leguminosarum. They transferred both idhA and mosB genes in plants root via Rhizobium rhizogenes (rather than Agrobacterium rhizogenes, line 167, and check elsewhere in the manuscript, e.g. in Methods section) to generate transgenic roots that produced SI. A very faint production of SI was observed in both the leaves (figure 2f) and roots (figure 3a) of Medicago truncatula, or in the roots of transgenic seedlings of Hordeum vulgare (barley; figure 3b). Though the amounts of SI produced seem quite low as compared to those of IO, they were sufficient to induce the expression of the lux gene in the above mentioned reporter strain. Though I am convinced by the reported results, some data on the quantification of the produced SI are missing. I also think that it is definitively critical to verify that SO does not induce the expression of the reporter mosB::lux gene at the concentration detected in the plant assays, a data that I may have missed in the manuscript. Without this verification, the paper would lost a lot of his interest.

Overall, the submitted paper report a sizeable set of data that represent large amounts of work. This study definitively brings useful information for the scientific community interested in plant-bacteria interactions. Though very well written, the manuscript reads sometimes with difficulty mostly because it aggregates diverse information that range from plant biology to enzymology and from microbiology to chemistry. These data are also presented in a very synthetic way (e.g. figure 2a-g), likely due to the journal instructions to authors. In relation, some additional details and explanation of results are missing. I pointed out these missing points in the upper paragraphs. These need to be addressed before a possible publication of this work.

Beyond, if a want to play the devil's advocate, I would say that I believe, from the literature cited, that the authors originally thought of creating a trophic relationship between the engineered plants and bacteria, as done with the Agrobacterium opines. Because only SI could be produced in plants, and because the technique only generated very low levels of rhizopine, the establishment of a this trophic relationship is most likely not feasible. Consequently, the presentation trick here is to show that this very low level suffice to induce engineered genes in the bacteria. I nevertheless believe that the paper report valuable data, as I stated above. The true novelty of the work mostly lies in the identification of the biosynthetic pathway for 3MSI, the resolution of the enantiomers, and the demonstration that the pathway can be transferred from bacteria to plants, though with a very low efficiency.

Reviewers' comments:

Reviewer #1 (Remarks to the Author):

In some Rhizobium-legume symbioses, compounds known as rhizopines are synthesized by bacteroids in nodules and are subsequently catabolized by free-living cells of the producing strain. It has been proposed long ago that plant-produced rhizopines, and Agrobacterium opines as well, may be used to promote the competitiveness of an opine-catabolizing strain in the rhizosphere.
Along the same line, Geddes and co-workers propose to use a plant-produced rhizopine to control gene expression in rhizospheric bacteria, for the delivery of new function to plants such as, for example, biological nitrogen fixation. In this long-term perspective, the authors have engineered the synthesis of a specific rhizopine (scyllo-inosamine) in hairy roots of Medicago sativa and primary transformants of barley and shown that the rhizopine is secreted in the rhizosphere, as assessed with a rhizopine-biosensor strain.

General comments:
1. Novelty:
The use of a rhizopine to control bacterial gene expression in the rhizophere is a novel extension of the opine concept. Here the authors show that a rhizopine synthesized in engineered plants is secreted into the rhizosphere. Whether this will indeed allow controlling bacterial gene expression for the benefit of the plant remains however to be demonstrated.
The transkingdom nature of the signalling is novel for a rhizopine. Yet this mimicks the situation in Agrobacterium in which bacterial opine-synthesizing genes are naturally transferred to the host cells.
A novel synthetic pathway for the production of the rhizopine scyllo-inosamine was engineered in plants, although the stability of the transgenes was not assessed at this stage.
As another piece of novelty, the rhizopine biosynthetic pathway was clarified and the role of new genes (mosDEF) elucidated in Sinorhizobium meliloti but this is not central to the paper.

2. Evidence for conclusions:
Better –and more- plant images (Fig 1b, Fig3cd) and additional controls are needed to strenghten the conclusions (see specific comments below).
The amount of rhizopine compounds produced by the plants and the bacterial bioluminescence should be quantified and variability assessed. It was not clear to me whether the level of rhizopine production as detected by GCMS in nodules or barley roots was compatible with the rhizopine concentration needed to activate the fusion.

- Magnified images of nodules of nodules in **Figure 1b** and included, and images of 5 different plants from the experiment with a representative image in **Figure 1b**, as well as close-ups of nodules from all of them have been included in **Supplemental Fig. 1.**
- The mean luminescence observed from transgenic roots of *M. truncatula* hairy roots and T_0 barley plants inoculated with rhizopine lux biosensor are 0.23 cps/mm^2 [SE = 0.07, N = 5 plants] and 1.68 cps/mm^2 [SE = 0.37, N = 7 plants] respectively.
- *M. truncatula* hairy roots and T_0 barley plants were found to produce 27.39 ng/mg dry weight [SE = 9.83; N = 3] and 6.55 ng/mg dry weight [SE = 0.43; n = 10] of rhizopine in their transgenic roots respectively. These data have been included in text in the manuscript.
- In addition, we performed confocal imaging to visualize rhizopine mediated plant to bacteria signalling on transgenic barley roots. Confocal images clearly revealed that rhizopine produced in engineered roots of T1 barley plants induce GFP fluorescence at a significant level in most of rhizopine GFP biosensor colonies visualized on the root surface (**Fig. 4a & 4b)**.
-

3. Significance:
The main significance of the work is in a long-term biotech perspective.

Specific comments:
4. l. 67-76. Evidence that the rhizopine is secreted from nodules of M. sativa needs additional controls. Was bioluminescence only observed at the nodule surface?

- *R. leguminosarum* does not enter *M. truncatula* nodules so the rhizopine biosensor in *R. leguminosarum* cannot report on the presence of rhizopine inside the nodule (only on the surface). We made a rhizopine biosensor coupled to GFP and put it into *S. meliloti* L5-30 (i.e. the same strain that nodulates and makes

rhizopine inside nodules) and we observed bright fluorescence inside the nodule. This is expected in nodules because rhizopine is made by bacteroids under the control of the master regulator of nitrogen fixation, NifA. The point of figure 1b is that it is the first demonstration that rhizopine, while made inside nodules, comes out of nodules into the rhizosphere. That this conclusion is undoubtedly correct is proven by **Figure 3C** where transgenic *M. truncatula* also secretes rhizopine into the rhizosphere i.e. there are no nodules because only the non-nodulating *R. leguminosarum* containing the biosensor is present. It is then also confirmed with **Figure 3D** where barley, which cannot nodulate also shows a clear positive response from our biosensor strain of *R. leguminosarum.*

Could the pOPS0046 reporter plasmid be transferred between Rlv3841 and S. meliloti L5-30 in the rhizosphere?

- The plasmid does not contain genes for transfer (Tra) and can only be mobilised when *tra* genes are provided in trans (these are not present in any of the two strains used in this experiment) so the plasmid cannot be transferred from *R. leguminosarum* to *S. meliloti* L530.

Has a non-nodulating mutant of S. meliloti L5-30 been used as a negative control?

- Since rhizopine is only produced in nodules by L530 (rhizopine production in L530 it is under the control of nodule activated NifA), a non-nodulating mutant of L530 cannot produce nodules and cannot produce rhizopine. As a nod mutant of L530 cannot serve as a control, instead we used a rhizopine mutant of *S. meliloti* L530 (i.e. it nodulates but cannot produce rhizopine) and as expected the rhizopine biosensor in *R. leguminosarum* produced no luminescence. Thus we show that the luminescence measured on the surface of nodules of *M. truncatula* by *R. leguminosarum* requires rhizopine production inside the nodule by *S. meliloti* L530.

5. The picture in Figure 1b should be improved (nodules are barely visible). Only 3 plants have been tested. Do all nodules respond the same?

- **Figure 1b** has now been improved with magnification of a portion of the image, and further images of 5 different plants all showing the same response, with close-ups of nodules has now been included in **Supplementary Fig. 1**

6. Fig1bc: how does the bioluminescence in situ (Fig 1b) compare with the bioluminescence in fig 1c?

- It is not possible to directly compare between in situ bioluminescence and bioluminescence in culture, as total bioluminescence observed is dependant on cell number (not readily assessable in situ) as well as the metabolic state of the bacteria (which will be very different in situ compared to in free-living culture where nutrients are not limiting) since light production is ATP dependant. (See Pini *et al.* Plant Physiology (2017) 174:1289).

7. l. 77- Fig 1c: Was scyllo-inosose 7 (SO) tested for signaling activity? (see below)

- We attempted to chemically synthesise *scyllo*-inosose, but we found, as others have (S. J. Angyal and D. Range, Carbohydr. Res., (1979) 76: 121–130 and G. G. Post and L. Anderson, J. Am. Chem. Soc., (1962) 84, 471–478), that it is unstable in aqueous solution. Thus we were unable to measure SO signalling in bacterial culture. However, we were able to observe *scyllo*-inosose accumulation in transgenic plants when *idhA* was expressed (**Fig. 2f, 3a & 3b, Supplemental Fig. 8**). When the rhizopine lux biosensor was inoculated on the transgenic roots constitutively expressing *idhA* alone (No MosB for SIA accumulation), no luminesce was observed. These data are now included in **Supplementary Fig. 12**.

8. Fig1d: rhizopines are best active on the reporter fusion in the 0.1 to 1mM range. At high concentrations, the molecule can be both a signal and a substrate. Could the reporter Rlv3841 strain use rhizopine for growth in these conditions, with an impact on the global bioluminescence?

- Rlv3841 is not able to use rhizopines as a substrate- it does not contain the appropriate catabolic genes, and was unable to grow using rhizopine as a substrate when we tested it in the laboratory.

9. Fig2: There is little difference in the spectra generated by the IDH and IDH-MosB constructs. Similarly, little SIA1 seems to be produced in Medicago hairy roots (Fig 3a) or transgenic barley roots (Fig 3b). Therefore to which extent plants produce SIA1 is not clear to me. Alternatively, could bioluminescence be due to secreted SO?

- EIC (m/z 245) chromatograms of extracts prepared from tobacco leaves agro-infiltrated with either empty vector (control) or IDH or MosB or IDH-MosB together (now included in **Supplementary Fig. 10**) show more clearly that rhizopine peak was seen only when IDH-MosB are expressed together.
- The amount of rhizopine produced in engineered plant samples has been quantified and included in text (**line 215**):

  M. truncatula hairy roots - 27.39 ng/mg dry weight [SE = 9.83; N = 3]
  Barley T_0 roots - 6.55 ng/mg dry weight [SE = 0.43; n = 10]
  Barley T_1 roots - 7.00 ng/mg dry weight [SE = 0.17; n = 10, N = 2]
- No luminescence was observed when the rhizopine lux biosensor was inoculated on the transgenic roots constitutively expressing *idhA* alone (now included in **Supplementary Fig. 12**).

10. Fig3ab: how many roots have been assayed? Which portion of the roots (eg in barley) has been extracted?

- *M. truncatula* hairy roots - Four weeks after transformation, transgenic roots from composite *M. truncatula* plants grown in ModFP agar petridishes were harvested for GC MS analysis. Whole transgenic root from approximately ten composite plants were harvested and pooled together for analysis.
- Barley T_0 roots - Twelve weeks after transformation, transgenic roots from barley T_0 plantlets grown in ModFP agar petridishes (approximately seven days post transfer) were harvested for GC MS analysis. Whole root from each T_0 plantlets were harvested individually for analysis.
- Barley T_1 roots - Transgenic roots from approximately two weeks old barley T_1 plants grown in ModFP agar petridishes were harvested for GC MS analysis. Whole root from each T_1 plants were harvested individually for analysis.

11. Fig3cd: In addition to more detailed pictures, some quantification of plant-associated variation is needed. Maximum bioluminescence seems to be restricted to a very small part of the root system in barley (yellow spot). Was the reason for this investigated

- The mean luminescence observed from transgenic roots of *M. truncatula* hairy roots and T_0 barley plants with rhizopine lux biosensor are 0.23 cps/mm^2 [SE = 0.07, N = 5 plants] and 1.68 cps/mm^2 [SE = 0.37, N = 7 plants] respectively.
- Secretion from plant roots is highly zonal and depends on the compound (see Pini *et al.* Plant Physiology (2017) 174:1289). The main site of secretion, best observed in barley, is the mature root so luminescence appears in the middle of the root systems, not in root elongation or root differentiation zones.

12. l 400. P. sativum plants are mentioned but corresponding data are not shown.

- *P. sativum* plants were tested for rhizopine production with *R. leguminosarum* strains predicted to produce rhizopine but these data were not important to the core message of the manuscript and are in the supplemental information (**Supplemental Figure 5**)

Reviewer #2 (Remarks to the Author):

This ms describes incorporation of the rhizopine *scyllo-inosamine biosynthesis pathway into M. truncatula and barley, and detection of the signal molecule in the rhizosphere with a novel biosensor. This paper packs a lot of discovery into a small package. The authors have demonstrated the function of MosDEF and MosB, and the use of the MocB and MocR into a lux reporter vector to allow luminescent detection by bacteria in the rhizosphere. I recommend its publication, after attention is paid to some details below,*

Small complaints:

While the work on mosDEF and MosB is novel, the use of IolG is not. There is a great deal of literature on the structure and function of B. subtilis IolG in particular, the enzyme the authors chose to work with, but this recent (post-1979) work is not cited. I don't want to encourage over-referencing, but this seems odd, and misleading to the reader. The authors should also discuss if they considered working instead with MocA, which is predicted to be an NAD-dependent dehydrogenase very similar to IolG (and if not, why not).

- We have added additional recent citations for IolG and added mentioned this recent work in text (**line 165).** MocA was not considered as it is expected to perform an alternate function than inositol oxidation (oxidation of a rhizopine catabolic intermediate) during rhizopine catabolism (See Rossbach *et al.* 1994 Mol Gen Genet 245:11-24)

Line 87 states the "clear preference of the biosensor for [L over D] indicates that [L] is the naturally active enantiomer, at least in this context". I think this overstates what the data indicates. I would rather see a statement like "1L-2 has previously been proposed to be the naturally occurring enantiomer, and the preference for this enantiomer observed in figure 1 supports this."

- We have adjusted the wording to the suggested in text.

Figure 2: dehydrogenase reactions in parts d and g are incorrect. (Not FAD + 2 H+, NAD + H+)

- The reactions have been corrected in **Figure 2d** and **g**

Does reference 28 have an error?

- Reference removed while revising

In the file of Supporting Information I received, the structure of compound 11 is floating in the wrong place (line 186). It should be on line 193. There also looks to be some problem with the structure on lone 297, forcing a page break. See also line 304. It's a general problem. Maybe in-line-with-text would work better?

- These errors have been resolved in the SI

Reviewer #3 (Remarks to the Author):

Important note: in this report, I used my own abbreviations to describe the various compounds to which this study refers.

The paper by Geddes and co-workers describes the possible engineering of trans-kingdom signaling between plants and bacteria. There are indeed several interests in such engineering though I estimate that the road is still long to reach durable and reproducible agricultural applications.

The authors investigated the metabolism of scyllo-inosamine (SI) and 3-O-methyl-scyllo-inosamine (3MSI), two compounds known as a rhizopines. These molecules are found in nitrogen fixing nodules incited by a few rhizobia on a limited number of legume species. They are synthesized and degraded by the rhizobia, possibly as a way to attract "compatible" rhizobia to the plant roots.

The first reported experiment consisted in cloning the promotor of a gene involved in rhizopine production fused to a lux reporter gene in a Rhizobium leguminosarum background (the reporter). When co-inoculated with Sinorhizobium meliloti strain L5-30 on Medicago sativa, the reporter strain "sensed" the production of rhizopine in nodules incited by strain L5-30 but not in nodules incited by a mutant of L-30 unable to produce rhizopines. The results are convincing but the reasoning for the experiment is not clear. A priori, rhizopine detection should rather involve (promotor of) catabolic rather than anabolic genes… A few words to explain the rationale would therefore be beneficial.

- Following this rational, the rhizopine biosensor used a promoter from *mocB* which is a rhizopine catabolic gene, not an anabolic gene. We have adjusted the wording to help clarify this to the reader

The specificity of the reporter system was investigated next by exposing it to various molecules such as SI and 3MSI, and related compounds. Using synthetic and commercially available compounds, the authors convincingly demonstrated that the reporter is indeed specific for SI and 3MSI. The remarkable result here is that the reporter preferentially detects one of the two enantiomers of 3MSI, which is also the only enantiomer that promotes the growth of strain L5-30.

The next reported data dealt with the biosynthetic route of 3MSI and the related genetic determinants. By comparing sequences of rhizobial genomes, the authors noted that: (i), the mosA gene is not widely distributed amongst the sequences; (ii), the mosBCDEF genes are present in all strains synthesizing 3MSI; and (iii), the mosDEF genes could encode a membrane-associated dehydrogenase complex. The suggested involvement of mosDEF in 3MSI synthesis (rather than degradation) was demonstrated using a mosE mutant that does not produce rhizopines. Though I globally agree with the result and proposed pathway, I

found the presentation of the data quite complex. One of the reasons is the lack of consistency of abbreviations through figures and text. For example ononitol appears sometimes as D-ononitol, (+)-1D-4, or OL!

- We have updated all figures of GCMS data to be consistent in their compound naming with the main text of the manuscript (no abbreviations) to help with clarity during data interpretation

Regarding the experiment with the mutants, I noted the accumulation of an identical compound in the extracts obtained with either mosB and mosE (figure 2a). The authors indicate in the text (lines 122/127) that the compound should be the same as the one accumulated by a strain that does not produce 3MSI in which mosDEF genes have been transferred. I disagree with this claim considering that the retention time of the compound in the mutants is 15.4 min while it is 15.6 min in the later strain. My feeling - and this needs to be confirmed and shown in figure 2a - is that the peak at 15.6 is ononitol (OL) as stated in figure 2b, indeed.

- We have clarified with an added ononitol control in **Figure 2a** that the peak at 15.6 is ononitol. We intended to convey that the compound at 15.4 min observed when *mosDEF* genes were expressed in a non-rhizopine producing strain was identical to a third compound identified in nodules from wild-type plants – in addition to ononitol and 3-OMSI (at 15.4 in **Fig 2a**) that we expect is keto-ononitol, but this compound was not observed in mutants- in the case of the mutants we observed ononitol accumulating (at 15.6) (see next response). Some words have been adjusted to clarify this.

OL appeared in Fig 2a for both mosB and mosE just because the intermediate compound 3-O-methyl-scyllo-inosose (3MIO) is unstable and converted back to OL spontaneously. In agreement with my opinion, mosB should accumulate 3MIO while mosE should accumulate OL, unless 3MIO spontaneously yield OL. This feeling is confirmed by the observation of the 3MIO standard appears to contain large amounts of OL. In my opinion, the authors should clarify these points.

- The reviewer is correct- ononitol was observed to accumulate in the *mosB* and *mosE* mutants, not keto-ononitol. It may be the case as he suggests that spontaneous conversion to ononitol as observed in the chemical standard may be occurring, alternatively the *mosB* mutant is polar on *mosDEF* (prevents their transcription so *mosDEF* are not expressed in a *mosB* mutant). A line has been added in text to clarify this for the reader (**line 140-143**).

To evaluate the possibility to generate 3MSI-producing plants, the authors attempted to express transiently mosDEF and mosB in Nicotiana leaves. No production of 3-MSI was observed. To circumvent this bottleneck, the authors though to engineer SI (and not 3MSI) production in plants. They proposed a pathway that originates from myo-inositol, and involved the synthesis of scyllo-inosose (IO, the non methylated version of 3MIO) from this polyol, using the enzyme inositol dehydrogenase IolG from B. subtilis. The authors combined purified IolG and MosB enzymes and demonstrated the production of SI in vitro. I have one comment regarding this result. In figure 2e, I would have expected that the incubation of IolG would generate IO from myo-inositol. This is not the case. There is no comment on this issue in the text and I found that disturbing. A line to explain the result would be beneficial.

- In aqueous solution, chemically synthesised *scyllo*-inosose, was found by us, and by others (S. J. Angyal and D. Range, Carbohydr. Res., 1979, 76, 121–130 and G. G. Post and L. Anderson, J. Am. Chem. Soc., 1962, 84, 471–478) to be unstable in aqueous solutions. We expect this is the reason we did not observe it in GC-MS reactions that included the inositol dehydrogenase IolG. Nevertheless, in plants expressing the inositol dehydrogenase *idhA* alone we did observe *scyllo*-inosose production and have now included this data in **Supplemental Figure 8**. We've added a line in text addressing the absence of SI in the in vitro assay with IolG (**line 172-176**).

Furthermore, the authors indicated that they expected this pathway to work into plants but did not report the results obtained with the two above enzymes. I suspect that this enzyme couple did not allow the production of SI in plants, possibly due to GC% or codon usage characteristics of the encoding genes. Here also a comment would be welcome.

- Both inositol deydrogenases from *Bacillus subtilis* (IolG) and *R. leguminosaurm* (IdhA) were tested in plants during pilot studies (now included in **Supplementary Fig 8**.) The chromatogram intensity and mass spectrum of the *scyllo*-inosose produced in tobacco leaves agro-infiltrated with either IdhA or IolG were similar to each other showing both dehydrogenases work, and plant work proceeded with IdhA. Concurrently, both dehydrogenases were also purified and tested *in vitro*, but purified IolG showed superior kinetics *in vitro* (now included in **Supplementary Fig. 9**) and so was used in the *in vitro* assays we performed. Lines in text have been added to help guide the reader through this logic (**line 167-172**).

The authors next used another inositol dehydrogenase, namely IdhA from R. leguminosarum. They transferred both idhA and mosB genes in plants root via Rhizobium rhizogenes (rather than Agrobacterium rhizogenes, line 167, and check elsewhere in the manuscript, e.g. in Methods section) to generate transgenic roots that produced SI. A very faint production of SI was observed in both the leaves (figure 2f) and roots (figure 3a) of Medicago truncatula, or in the roots of transgenic seedlings of Hordeum vulgare (barley; figure 3b). Though the amounts of SI produced seem quite low as compared to those of IO, they were sufficient to induce the expression of the lux gene in the above mentioned reporter strain. Though I am convinced by the reported results, some data on the quantification of the produced SI are missing.

- Amounts produced have been quantified and included in text (**line 199 and 215**). The amount of rhizopine produced in engineered plant samples were:
  M. truncatula hairy roots - 27.39 ng/mg dry weight [SE = 9.83; N = 3]
  Barley T_0 roots - 6.55 ng/mg dry weight [SE = 0.43; n = 10]
  Barley T_1 roots - 7.00 ng/mg dry weight [SE = 0.17; n = 10, N = 2]

I also think that it is definitively critical to verify that SO does not induce the expression of the reporter mosB::lux gene at the concentration detected in the plant assays, a data that I may have missed in the manuscript. Without this verification, the paper would lost a lot of his interest.

- While we were unable to investigate the induction by SO in bacterial culture due to the instability of synthesised SO, we did observe SO production in transgenic plants expressing IdhA (**Fig. 2f, 3a & 3b**).
- We inoculated the rhizopine lux biosensor on transgenic roots constitutively expressing *idhA* alone (no MosB for SIA accumulation) and observed no luminescence (now included in **Supplementary Fig. 12**), demonstrating that SO does not induce the lux reporter in the biosensor strain.
-

Overall, the submitted paper report a sizeable set of data that represent large amounts of work. This study definitively brings useful information for the scientific community interested in plant-bacteria interactions. Though very well written, the manuscript reads sometimes with difficulty mostly because it aggregates diverse information that range from plant biology to enzymology and from microbiology to chemistry. These data are also presented in a very synthetic way (e.g. figure 2a-g), likely due to the journal instructions to authors. In relation, some additional details and explanation of results are missing. I pointed out these missing points in the upper paragraphs. These need to be addressed before a possible publication of this work.

Beyond, if a want to play the devil's advocate, I would say that I believe, from the literature cited, that the authors originally thought of creating a trophic relationship between the engineered plants and bacteria, as done with the Agrobacterium opines. Because only SI could be produced in plants, and because the technique only generated very low levels of rhizopine, the establishment of a this trophic relationship is most likely not feasible. Consequently, the presentation trick here is to show that this very low level suffice to induce engineered genes in the bacteria. I nevertheless believe that the paper report valuable data, as I stated above. The true novelty of the work mostly lies in the identification of the biosynthetic pathway for 3MSI, the resolution of the enantiomers, and the demonstration that the pathway can be transferred from bacteria to plants, though with a very low efficiency.

- Our aim was not to create a trophic relationship such as seen with opines. Production of large amounts of rhizopine, which is a potential carbon and nitrogen source, would lead to strong selection pressure for other bacteria to acquire the ability to degrade it. We therefore believe it is an advantage for rhizopine to be a signal controlling expression but not to be produced in large amounts. Furthermore, substantial production of rhizopine would also have a potential yield penalty for the plant.

Reviewers' comments:

Reviewer #1 (Remarks to the Author):

The manuscript has significantly been improved with more and better figures, more quantification and more SI supplied.
A couple of issues emerged from the revision that may require clarification/comments
A new rhizopine synthesizing pathway has been engineered in both Medicago and barley roots, yet with different efficiencies. Medicago hairy roots produce ca 4 fold more rhizopine than To barley roots (27.4 ng/mg dry weight vs 6.55 ng/mg). Could the authors comment on this?
Despite Medicago roots producing more rhizopine than barley, the fluorescence in the barley rhizosphere is ca 6 times higher than in the Medicago's (1.68 vs 0.23 vcps/mm2). Is bioluminescence a faithful way to investigate rhizopine exudation in the rhizophere then? Was the rhizopine concentration directly (eg by GCMS) quantified in the rhizosphere of the two plants?

Miscellaneous:
Fig 1b: I was simply asking for experimental controls, not arguments, to ensure that rhizopines were indeed secreted from nodules. The mosB mutant of S. meliloti L5-30 controls that the reporter gene expression depends on the rhizopine but does not prove rhizopine is secreted. Yet I agree this is most likely.
Legend of Fig.1c: the sentence starting with "A magnified portion…" refers to fig1b and duplicates information.
Fig 1cd: Fluorescence values are expressed in rlu (relative luminescence units) for which I did not find the definition in the SI material. How does that compare to cps/mm2?
Fig 3 c: more focused images are needed.
l. 201 "robustly" might be too strong. Only half of the Medicago plants show a low level of fluorescence.
Fig 4: Was the fluorescence in the Medicago rhizosphere detectable by fluorescence microscopy? If not, this should be acknowledged.
l.65 check sentence.

Reviewer #2 (Remarks to the Author):

All points raised in my initial review have been addressed appropriately.

Reviewer #3 (Remarks to the Author):

I have read with interest the revised version of the manuscript by Geddes and co-workers and found that most if not all the comments I made in my previous review were taken into account satisfactorily. I allow myself to congratulate the authors for this very interesting work on engineered plant-microbe interactions.

>The second and third referees were happy with the manuscript so we only reply to the first referee. Our replies follow >.

The manuscript has significantly been improved with more and better figures, more quantification and more SI supplied.
A couple of issues emerged from the revision that may require clarification/comments
A new rhizopine synthesizing pathway has been engineered in both Medicago and barley roots, yet with different efficiencies. Medicago hairy roots produce ca 4 fold more rhizopine than To barley roots (27.4 ng/mg dry weight vs 6.55 ng/mg). Could the authors comment on this?
Despite Medicago roots producing more rhizopine than barley, the fluorescence in the barley rhizosphere is ca 6 times higher than in the Medicago's (1.68 vs 0.23 vcps/mm2). Is bioluminescence a faithful way to investigate rhizopine exudation in the rhizophere then? Was the rhizopine concentration directly (eg by GCMS) quantified in the rhizosphere of the two plants?

>The values for rhizopine are corrected per unit weight of each plant. Barley roots have a much greater mass so it is not really possible to make a direct comparison between root systems. However, it is the greater mass of the barley root that brings the luminescence per unit root weight down. The Barley roots show a stronger signal where rhizopine is being released, which is concentrated in the central part of the barley root and not elsewhere. This also explains the lower value for fluorescence per unit mass i.e. substantial parts of the lower younger root are not yet secreting rhizopine. Rhizopine has been quantified in total roots not in the rhizosphere which would be technically extremely challenging.

Miscellaneous:
Fig 1b: I was simply asking for experimental controls, not arguments, to ensure that rhizopines were indeed secreted from nodules. The mosB mutant of S. meliloti L5-30 controls that the reporter gene expression depends on the rhizopine but does not prove rhizopine is secreted. Yet I agree this is most likely.

> The luminescent reporter fusion is in Rhizobium leguminosarum, which cannot enter the nodules of Medicago truncatula, so it must report on the concentration in the rhizosphere. Likewise, barley does not have nodules so again Rhizobium leguminosarum can only be reporting on the concentration outside the root. This conclusion is also supported by the confocal imaging showing colonies fluorescing on the surface of barley roots.

Legend of Fig.1c: the sentence starting with “A magnified portion…” refers to fig1b and duplicates information.

>duplication removed

Fig 1cd: Fluorescence values are expressed in rlu (relative luminescence units) for which I did not find the definition in the SI material. How does that compare to cps/mm2?

>rlu is total luminescence per unit OD590nm of bacterial culture measured in a luminescence microplate reader and has now been mentioned in methods. Plate readers typically just give a total luminescence reading, which is different from the NightOwl system, which is a photon counting camera that records photons counted per unit time so counts per second and is given per square mm of the image. Descriptions of this have been added to the methods.

Fig 3 c: more focused images are needed.

>This is technically almost impossible as luminescence measurements with a photon counting camera always give a zoomed-out image to show the whole root. This technical limitation is why in the revised manusript we also provided close up confocal images (figure 4). Surely figures 3 and 4 should be considered in tandem? However, to help the reader we have now also added a magnified image of *Medicago truncatula* roots in Fig 3C.

l. 201 “robustly” might be too strong. Only half of the Medicago plants show a low level of fluorescence.

>We have removed robustly.

Fig 4: Was the fluorescence in the Medicago rhizosphere detectable by fluorescence microscopy? If not, this should be acknowledged.

>Fluorescence was only measured in the barley rhizosphere, not in the *Medicago* rhizosphere. This is because we have stable lines of barley not of *Medicago* (SI lines of which need to be generated by hairy root transformation). Ultimately our goal is to develop signalling in cereals so barley is the logical choice for these experiments. We feel that the lux luminescence images are sufficient to show production and secretion of SI in *Medicago.*

l.65 check sentence.

>corrected to “Rhizobia are able…”

REVIEWERS' COMMENTS:

Reviewer #1 (Remarks to the Author):

Thank you for clarifying the issue regarding the differences between barley and medicago. May be this could be clarified in the MS too.

I hope my comments have been useful.
